# Corrigendum to: Bipartite graphs in systems biology and medicine: a survey of methods and applications

**DOI:** 10.1093/gigascience/giz130

**Published:** 2020-01-20

Georgios A Pavlopoulos, Panagiota I Kontou, Athanasia Pavlopoulou, Costas Bouyioukos, Evripides Markou, Pantelis G Bagos *GigaScience*, Volume 7, Issue 4, 1 April 2018, giy014, https://doi.org/10.1093/gigascience/giy014.

The authors have revised the review article Pavlopoulos, GA, *et al*. *Bipartite graphs in systems biology and medicine: a survey of methods and applications*. GigaScience, 2018, 7:4, to address several issues in the published version.

The authors did not properly cite information throughout the original version. In particular, they borrowed text in the “Projection” section without sufficient attribution from the article: *The backbone of bipartite projections: Inferring relationships from co-authorship, co.-sponsorship, co-attendance and other co-behaviors* Soc Netw [Internet]. 2014 [cited 2018 Dec 17]; 39:84–97. by Zachary Neal. (Available from: https://linkinghub.elsevier.com/retrieve/pii/S0378873314000343).

In addition, reference #77 was changed. Klecka J, The role of a water bug, *Sigara striata*, in freshwater food webs. *Peer J* 2014;2: e389 has been replaced with Ings TC et al. Ecological networks–beyond food webs. *The Journal of animal ecology* 2009, 78(1):253–269.

Finally, the following two citations have been added, as they were mistakenly deleted during article revision:

- Hamaneh MB, Yu YK. Relating diseases by integrating gene associations and information flow through protein interaction network. *PLoS One* 2014, 9(10): e110936.
- Lee S, Lee KH, Song M, Lee D: Building the process-drug-side effect network to discover the relationship between biological processes and side effects. *BMC Bioinformatics* 2011, 12 Suppl 2: S2.

The order of subsequent references has changed as a result.

