# Supplementary material for: Bipartite graphs in systems biology and medicine: a survey of methods and applications
Source: Gigascience. 2018 Feb 19;7(4):giy014. doi: 10.1093/gigascience/giy014 (PMC6333914; doi:10.1093/gigascience/giy014)
Supplement: GIGA-D-17-00170_Revision_2.pdf [file giy014_giga-d-17-00170_revision_2.pdf]

GigaScience

Bipartite Graphs in Systems Biology and Medicine: a survey of methods and applications

--Manuscript Draft--

**Manuscript Number:**	GIGA-D-17-00170R2
**Full Title:**	Bipartite Graphs in Systems Biology and Medicine: a survey of methods and applications
**Article Type:**	Review
**Funding Information:**	U.S. Department of Energy (US) (DE-AC02-05CH11231) — Dr Georgios A Pavlopoulos
**Abstract:**	The latest advances of high-throughput techniques during the past decade allowed the Systems Biology field to expand significantly. Nowadays, the focus of biologists has shifted from the study of individual biological components to the study of complex biological systems and their dynamics at a larger scale. Through the discovery of novel bioentity relationships, researchers reveal new information about biological functions and processes. Graphs are widely used to represent bioentities like proteins, genes, small molecules, ligands and others as nodes and their connections as edges within a network. In this review, special focus is given on the usability of bipartite graphs and their impact in the field of Network Biology and Medicine. Furthermore, their topological properties and how these can be applied to certain biological case studies are discussed. Finally, available methodologies and software are presented and useful insights on how bipartite graphs can shape the path towards the solution of challenging biological problems are provided.
**Corresponding Author:**	Pantelis G Bagos, PhD<br>University of Thessaly<br>Lamia, GREECE
**Corresponding Author Secondary Information:**	
**Corresponding Author's Institution:**	University of Thessaly
**Corresponding Author's Secondary Institution:**	
**First Author:**	Georgios A Pavlopoulos, PhD
**First Author Secondary Information:**	
**Order of Authors:**	Georgios A Pavlopoulos, PhD
	Panagiota I Kontou, PhD
	Athanasia Pavlopoulou, PhD
	Costas Bouyioukos, PhD
	Evripides Markou, PhD
	Pantelis G Bagos, PhD
**Order of Authors Secondary Information:**	
**Response to Reviewers:**	Dear Editor,<br>We have updated the manuscript in order to meet your comments. We added the URLs in the references, we tried to improve the language, and we added the "availability of data" along with the supplementary data.<br>Thank you in advance for your help and efforts<br>Regards<br>Pantelis Bagos
**Additional Information:**	
**Question**	**Response**

Powered by Editorial Manager® and ProduXion Manager® from Aries Systems Corporation

Are you submitting this manuscript to a special series or article collection?	No
**Experimental design and statistics**<br><br>Full details of the experimental design and statistical methods used should be given in the Methods section, as detailed in our Minimum Standards Reporting Checklist. Information essential to interpreting the data presented should be made available in the figure legends.<br><br>Have you included all the information requested in your manuscript?	Yes
**Resources**<br><br>A description of all resources used, including antibodies, cell lines, animals and software tools, with enough information to allow them to be uniquely identified, should be included in the Methods section. Authors are strongly encouraged to cite Research Resource Identifiers (RRIDs) for antibodies, model organisms and tools, where possible.<br><br>Have you included the information requested as detailed in our Minimum Standards Reporting Checklist?	Yes
**Availability of data and materials**<br><br>All datasets and code on which the conclusions of the paper rely must be either included in your submission or deposited in publicly available repositories (where available and ethically appropriate), referencing such data using a unique identifier in the references and in the "Availability of Data and Materials" section of your manuscript.<br><br>Have you have met the above requirement as detailed in our Minimum Standards Reporting Checklist?	Yes

Manuscript
Click here to download Manuscript GigaScience_final_version12-2.docx

Bipartite Graphs in Systems Biology and Medicine: a survey of methods and applications

Georgios A. Pavlopoulos[1,*,±], Panagiota I. Kontou[2,±], Athanasia Pavlopoulou[3], Costas Bouyioukos[4], Evripides Markou[2], Pantelis G. Bagos[2,*]

1. Lawrence Berkeley Labs, DOE Joint Genome Institute, 2800 Mitchell Drive, Walnut Creek, CA 94598, USA
2. University of Thessaly, Department of Computer Science and Biomedical Informatics, Papasiopoulou 2-4, Lamia, 35100, Greece
3. Izmir International Biomedicine and Genome Institute (iBG-Izmir), Dokuz Eylül University, 35340, Turkey
4. Université Paris Diderot, Sorbonne Paris Cité, Epigenetics and Cell Fate, UMR7216, CNRS, France

GAP: g.pavlopoulos@lbl.gov
PK: pkontou@compgen.org
AP: athanasia.pavlopoulou@deu.edu.tr
CB: costas.bouyioukos@univ-paris-diderot.fr
EM: emarkou@dib.uth.gr
PGB: pbagos@compgen.org

*Corresponding authors
±These authors contributed equally to the work

Abstract

The latest advances of high-throughput techniques during the past decade allowed the Systems Biology field to expand significantly. Nowadays, the focus of biologists has shifted from the study of individual biological components to the study of complex biological systems and their dynamics at a larger scale. Through the discovery of novel bioentity relationships, researchers reveal new information about biological functions and processes. Graphs are widely used to represent bioentities like proteins, genes, small molecules, ligands and others as nodes and their connections as edges within a network. In this review, special focus is given on the usability of bipartite graphs and their impact in the field of Network Biology and Medicine. Furthermore, their topological properties and how these can be applied to certain biological case studies are discussed. Finally, available methodologies and software are presented and useful insights on how bipartite graphs can shape the path towards the solution of challenging biological problems are provided.

Keywords

Biological Networks, Graph Theory, Systems Biology, Bipartite Graphs, Ecological networks, Network Medicine

Background

Nowadays, in the big-data and OMICS- era, established high-throughput technological advances, Integrative Biology and Bioinformatics have significantly changed our view on how to tackle difficult biological problems towards the understanding of more complex biological systems. For example, yeast-two-hybrid [1] and protein chips [2] have enabled biologists to experimentally detect the complete protein interactome or protein-protein interactions (PPIs) for certain organisms [3-7]. Microarrays and RNA-seq [8] have accelerated the discovery of differentially expressed genes across different conditions (i.e. disease vs. control) and the study of developmental processes, as well as pharmacogenomic responses and evolution of gene regulation in different species. In this way, through the generation of gene regulation networks, new knowledge about gene behavior and unknown functions can be unraveled. Furthermore, latest drug screening and mass spectrometry techniques allow for a massively parallel protein-compound interaction identification and exploration, whereas genome sequencing technologies [9] have exponentially increased the number of newly sequenced genomes. Therefore, exploration and discovery of new genes, new lineages of life, identification of single nucleotide polymorphisms (SNPs) or variations causative for genetic disorders [10], population genetics, characterization of the genetic material recovered from environmental metagenomic samples [11] and direct interspecies genome comparisons have opened new research fields while, simultaneously, have changed the landscape of bioentity associations known till today.

Protein-protein interactions (PPIs), gene expression, gene regulation, literature co-occurrences, evolutionary relationships, signal transduction, metabolic pathways and others are often captured in network representations, where a node represents a bioentity and an edge the relationship between them. PPIs, for example, are represented as simple undirected graphs, whereas gene signal transduction and regulation networks as directed graphs (digraphs). Additionally, gene expression networks can be found as weighted graphs, pathways as petri-nets and gene regulation together with literature co-occurrences as semantic graphs. Finally, multi-edged networks can hold information about nodes which are connected in multiple ways. For example, two proteins might co-occur in biomedical literature, share common domains, have a certain degree of sequence similarity, be evolutionary related and interact physically. For a better understanding of the definitions of the aforementioned networks, as defined by graph theory, more detailed descriptions are available elsewhere [12, 13].

In this review, we thoroughly discuss the potential and the usability of bipartite graphs for analyzing biological networks. To our knowledge, this is the first extensive investigation on bipartite graphs, given that other studies have focused on generic graph analysis. A bipartite graph, also referred to as “bigraph”, comprises a set of graph vertices decomposed into two

disjoint sets such that no two graph vertices within the same set are adjacent. As discussed in [14, 15], applications of such bipartite graphs can range from the representation of enzyme-reaction links in metabolic pathways to gene-disease associations or an ecological network. While network analyses have focused mainly on unipartite (one-mode) networks, considerably less attention has been paid to the deeper study of bipartite networks and their potential in biological sciences.

Many non-biological real-world networks may be naturally viewed and modeled by a bipartite graph structure. Perhaps the oldest example of such bipartite network originates from the analysis of Deep South data, also known as the ‘Southern Women’ data, collected in 1941, representing a set of women attending social events over a period of nine months [16]. Other notable examples studied extensively in the literature include for instance for the actors–movies network, where each actor was linked to the movies he/she appeared in [17, 18], the scientists-papers networks, where the scientists were linked to the paper they authored [18-20], the board-directors networks, where the members of the board of directors are linked to the companies they lead [21, 22], the peer-to peer exchange networks in which peers are linked to the data they provide [23], the world cities hosting branches of multinational firms [24], the supreme court justices joining majority opinions [25] and the legislators sponsoring bills [26]. Moreover, during the last years the bipartite graph has been used extensively in internet technology and applications since it has been used to model the relationship between queries and URLs in query logs [27], between video shots and tags [28], for entities and co-lists in Web pages [29], for users and items in recommendation [30], for behavior analysis of internet traffic [31] and for detecting network traffic anomalies [32].

The current review is structured as follows: a) we provide a mathematical definition of a bipartite graph, b) we comment on its topological properties, c) we summarize several projection strategies to generate two unipartite networks from a bipartite one; d) we discuss the theoretical properties and the importance of the projections, as well as the potential biological applications of them, e) we describe several real-life network types and how these can be analyzed using the graph theory related to bipartite graphs, f) we describe models and algorithms for bipartite graphs and finally, g) we comment on the advantages of available software dedicated to analyze bipartite networks.

Bipartite-graphs

Definition

A graph *G*=(*U*, *V*, *E*) is called *bipartite* (or *bigraph* or *two-mode network*) if its vertices can be divided into two disjoint sets, *U* and *V*, such that every edge (*E*) connects a vertex in *U* to one in *V* (Figure 1A,B). Vertex sets *U* and *V* are usually termed as the parts of the graph. Equivalently, a graph that does not contain any odd-length cycles is by definition a bipartite graph, whereas bipartite graphs are also equivalent to two colorable graphs. Among the various types of graphs, trees, acyclic graphs and circular graphs with an even number of vertices, are by definition

bipartite. A bipartite graph represents a special case of a *k*-partite graph with *k*=2. If a bipartite graph is not connected, it may have more than one bipartition; in this case, the (*U*, *V*, *E*) notation is helpful in specifying one particular bipartition that may be of importance in an application. If |*U*| = |*V*|, that is, if the two subsets have equal cardinality, then *G* is called a balanced bipartite graph. If all vertices on the same side of the bipartition have the same degree, then *G* is called biregular.

Bipartite graphs can be efficiently represented by biadjacency matrices (Figure 1C,1D). The biadjacency matrix *B* that describes a bipartite graph *G*=(*U, V, E*) is a (0,1)-matrix of size |U| × |V|, where B_{ik}=1 provided there is an edge between *i* and *k* or B_{ik}=0, otherwise. Biadjacency matrices can be used to describe equivalences between bipartite graphs, hypergraphs, and directed graphs. In most of the cases, biadjacency matrices are (0,1)-matrices and the networks are, therefore, unweighted. However, in some applications, as in the case of ecological networks, matrices with B_{ik}>1 are also used to represent a weighted bipartite network.

Properties of bipartite graphs

Bipartite graphs, as opposed to generic networks which have their own topological characteristics, comprise a distinct category, with their very own unique properties. Given that network metrics for unipartite networks have been studied extensively, herein attention is given to network metrics used specifically for bipartite graphs. A short commentary on such topological features is provided below [12, 33, 34]. Of note, there are dozens of specialized metrics for bipartite ecological networks, some of which are discussed in-depth in Dormann et al. [35]. Notably, known tools dedicated to automated topological analysis for generic networks are the Network Analysis Profiler (NAP) [36], Cytoscape's Network Analyzer [37], the Stanford Network Analysis Platform (SNAP) [38] and the igraph library (http://igraph.org/c). Although, these are not bipartite graph-specific, they do offer a wide spectrum of functions and modules related to topological analyses.

Degree

In a simple undirected graph, the degree or degree centrality is defined as the number of edges incident upon a node. Nodes with the highest degree (i.e.,connected to more nodes) are considered as "hubs". In a directed graph, the degree can be calculated as the sum of the in-degree (number of incoming edges) and the out-degree (number of outcoming edges). As opposed to a fully connected graph *G=(V,E)* which can have a maximum of $|V|(|V|-1)/2$ connections, in a bipartite graph, the maximum degree of a node can be equal to the number of nodes from the opposing set (*max[deg(u)]*=|*V*| or *max[deg(V)]*=|*u*|). Furthermore, the sum of the degrees of the first part is equal to the sum of the degrees of the opposing part, and both are equal to the cardinality of the edge set:

$$\sum_{v \in V} \mathbf{deg}(v) = \sum_{u \in U} \mathbf{deg}(u) = |E|$$

Closeness Centrality

It is a measure to determine whether a node can communicate with other nodes within the network readily and through short paths. Hence, the more central a node is, the closer is to all other nodes. Closeness centrality is inversely proportional to the shortest path length between two nodes. In a

bipartite graph, a node can have a minimum distance ‘1’ from vertices of the opposing set and ‘2’ from vertices of the same set. Moreover, due to the bipartite structure, all paths between nodes of the same set are of even length, a property that rather complicates the calculation of several measures.

Betweenness Centrality

The nodes with high betweenness centrality are the ones that serve as bridges between two highly connected communities. An all-against-all shortest path calculation is often required in order to estimate reliably betweenness centrality. Hence, each node increases its centrality score every time it is involved in a shortest path. The nodes with very high betweenness centrality scores are the ones that serve as mediators between two or more neighborhoods. In a bipartite graph, paths can originate and terminate at a node of each vertex set.

Eigenvector Centrality

It is a measure to identify the nodes that are connected to ‘important nodes’, such as hubs, within a network. The eigenvector centrality of a node is proportional to the sum of centralities of the nodes it is adjacent to. Bipartite eigenvector centrality is further reviewed in Daugulis [39].

Clustering coefficient

The global clustering coefficient indicates the tendency of a network to form tight clusters. Similarly, the local clustering coefficient shows the tendency of a node to belong to a cluster. While this is a useful measure for a generic network, applying the two clustering coefficients directly to a bipartite network is meaningless. Handling a two-mode network as an one-mode one is not recommended, as projected two-mode networks tend to have more and larger fully-connected cliques [40]. Moreover, the conventional clustering coefficient cannot be used in bipartite networks, where cycles of size three are absent. Instead, other coefficients based on the fraction of cycles with size four have been proposed, with similar clustering properties [41]. To overcome such problems, a number of clustering coefficients for two-mode networks have been proposed elsewhere [33, 41-44].

Nestedness

Nestedness is an important property of ecological networks. It is usually defined as a pattern of interactions in which ‘specialists’ (for example, pollinators that visit few plants) interact with subsets of the species with which ‘generalists’ (for example, pollinators that visit many plants) interact. Nestedness is not a metric in itself but a concept which, at least up to date, has not been formally defined through mathematical relationships. This probably explains the fact that there are several distinct metrics by which it can be measured. In mathematical terms, nestedness can be defined as a property of the previously mentioned biadjacency matrix *B*. If *B* is a perfectly nested binary matrix, then there exists a permutation of rows and columns such that the set of edges in each row *i* contains the edges in row *i*+1, while the set of edges in each column *j* contains those in column *j*+1. In particular, the rows and columns of *B* can be sorted (with $B_{1,j}>0\ \forall j$ and $B_{i,1}>0\ \forall i$) such that $B_{i,j} \leq \min(B_{i,j-1}, B_{i-1,j})$, a property that can be extended to quantitative matrices as well [45]. Thus, in general, a nested structure corresponds to a systematic arrangement of non-zero entries in the binary matrix often used to represent a network. However, measuring the

nestedness of a given network is not always straightforward and there are several detection methods for identifying nested patterns among other possible matrix arrangements [46]. A schematic representation is shown in Figure 2.

The most widely used metric of nestedness is the nestedness temperature, T=1-N, which quantifies whether the observed arrangement of 1's and 0's deviates from the arrangement given by an isocline that describes a perfect nestedness benchmark. Contributions of unexpected absences and presences in the upper-left and bottom-right sides, respectively, are weighted by their squared Euclidian distances from the isocline [47]. Similar metrics were presented by Araujo and coworkers [48], whereas fast algorithms and software for calculating T were presented by Guimarães and Guimaraes [49].

An additional metric, C, is based on the concept of "species richness" and, unlike T, quantifies nestedness exclusively between rows [50].

NODF (Nestedness metric based on Overlap and Decreasing Fill) was developed later in order to overcome two major disadvantages of previous methods: i) marginal totals may differ among columns and/or rows, and ii) the presences (1's) in less-filled columns and rows may coincide with those found in the more-filled columns and rows, respectively. Therefore, *NODF* has some important features that distinguish it from the preceding metrics, that is, it calculates nestedness independently among rows and columns, which allows the evaluation of nestedness only among sites (i.e. species composition) or among species (i.e. species occupancy), whereas it is able to evaluate how nested is one or more column (or row) in relation to other ones [51].

A modified version of *NODF*, termed *WNODF* (where 'W' stands for 'Weighted'), was also developed later to handle quantitative matrices [52] Other approaches have also been developed for the same task, including methods that rely on the eigenvalues and the spectral radius of the matrix [45, 53].

Modularity

Modularity is another feature usually found in ecological networks. Modularity occurs when certain groups of nodes (usually species) within a network are much more highly connected to each other than they are to other nodes of the network, with weak interactions among different modules (**Figure 3**). Modularity measures the tendency of a network to divide into modules (also called groups, clusters or communities). In networks with high modularity, the nodes within modules are densely connected, but sparsely connected in different modules. The most widely used measure of modularity is calculated from the (symmetric) adjacency matrix A by:

$$Q(\mathbf{A}) = \frac{1}{W}\sum_{C\in P}\sum_{i,j\in C}\left[A_{ij} - \frac{k_i k_j}{W}\right]$$

Where $W=\sum_{i,j}Aij$ and $k_i=\sum_j A_{ij}$ is the degree of node i. The indices i, j run over the nodes of the graph, whereas C runs over the communities (modules) of the partition. Since the adjacency matrix is not symmetric, $A=(O,B/B^T,O)$ can be applied. Hence, modularity enables the detection of modules (or clusters) in the first place , and it can be further optimized by particular optimization algorithms to detect community structure in networks (see below) [54].
Of particular note, networks can be both highly nested and highly modular [55] (Figure 4).

Internal links and pairs

A usual approach for the analysis of bipartite graphs consists of deriving unipartite graphs (projections) from the underlying bipartite structure (Figure 1C and 1D), which however is associated with important loss of information and data storage issues (below is provided a detailed description of projection).

Allali and coworkers [56] introduced the *internal links* and *pairs* as metrics useful for analyzing a bipartite graph, thereby providing an understanding of the projection of the bipartite graph. Specifically, in a bipartite graph $G=(U,V,E)$, (u,v) is a U-internal ($\perp$-) pair of G only if by adding the new link (u,v) to G does not change its U-projection; it is a U-internal link provided that the removal of the link (u,v) from G does not change its U-projection. The number of U-internal links of a node is called ‘U-internal degree’. The authors illustrated the relevance of these concepts in several real-world bipartite networks, highlighting their discriminative ability when benchmarked against random graphs. Internal links and pairs can be useful metrics for both modeling complex networks and storing them in a compact format [56].

Bipartivity

Many biological systems are naturally modeled as bipartite networks. However, there are also networks that although they are not naturally bipartite, they appear to be closer to bipartite compared to what can be expected by a completely random network; for instance, networks formed by two types of nodes which have a preference for interactions with nodes of the other type, such as networks of sexual relationships. It is possible to test whether a graph is bipartite and to return either a two-color graph (if it is bipartite) or an odd cycle graph (if it is not) in linear time, by using a depth-first search algorithm. The main idea is to assign to each vertex a color different from the color of its parent in a depth-first search tree in a preorder traversal of the tree. In this way, a two-color spanning tree consisting of edges connecting vertices to their parents is generated, although some of the non-tree edges may not be properly colored.

Bipartivity is a measure that quantifies how close a given network is to being bipartite. Two such measures were provided first by Holme and coworkers [57]. The first measure is based on the optimal two-coloring of the network [57]. The exact value of this quantity is NP-complete and, therefore, an optimal calculation is not possible. They proposed instead an approximate solution by a simulated annealing approach. The latter is based on the count of odd circuits that, in most cases, can be calculated in polynomial time. Later, Estrada and Velasquez provided a different measure, $\beta(G)$, based on the spectral decomposition of the biadjacency matrix [58]. This measure is easy to compute and allows the calculation of individual node contributions to global bipartivity, which is based on the concept of closed walks. Pisanski and Randic have taken into consideration the so-called Szeged index (Sz) and the revised Szeged index ($Sz*$), both of which can be considered generalizations of the Wiener number to cyclic structures. They found that the quotient of the two indices, termed $\sigma(G)$, can be used as a novel measure for characterizing the degree of bipartivity of networks, because the two indices assume the same values for bipartite graphs but different values for non-bipartite graphs. Thus, they proposed $\sigma(G)=Sz/Sz*$ as a measure of bipartivity and they also provided empirical evidence that it is in good agreement with $\beta(G)$ [59].

Ecological indices

In this section, some metrics that are routinely being used in ecological bipartite network analysis are mentioned. The symbol L indicates the number of realized links, whereas |U| and |V| denote

the number of species of each party in bipartite networks (e.g., hosts (U) vs. parasites (V)). *Connectance* (*C*) is the fraction of all possible links that are realized, $C=L/(|U|*|V|)$, which represents a standard measure of food web complexity. The related *linkage density* is defined as $D=L/(|U|+|V|)$.

In a food web of $|U|$ consumers and $|V|$ prey species, the mean number of prey species (links) per consumer is termed *generality*, given by $G = L/|U|$, and the mean links per prey *vulnerability*, given by $V = L/|V|$. The *web-asymmetry* defines the balance between numbers in the two levels and it is given by $W=(|V|-|U|)/(|U|+|V|)$, where positive numbers indicate more low-trophic level species and negative more high-trophic level species. Most of these metrics have also a weighted counterpart, whereas there are also several other metrics especially designed for quantitative interactions, such as *Shannon's evenness* (for measuring interactions), H_2 (a network-level measure of specialization based on the deviation of a species' realized number of interactions and that expected from each species' total number of interactions) and *niche overlap* (the mean similarity in the interaction patterns between species of the same trophic level). The reader can refer to key publications for more information on the topic of ecological indices [35, 60].

Projection

In a bipartite network, the nodes are divided into two disjoint sets (U, V), and the edges (E) connect nodes that belong to different sets. From a bipartite network, it is possible to derive two projected networks, where each one is composed of only one set of nodes. This approach for analyzing bipartite networks is termed 'projection', that is, deducing relationships between nodes of the same type. In other words, in order to study the relationships among a particular set of nodes, the bipartite network has to be compressed by one-mode projection.

The U one-mode projection ('U-projection' for short) is composed of a network containing only U-nodes, where two U-nodes are connected when they have at least one common neighboring V-node. Conversely, the V-projection is a network of V-nodes, in which two V-nodes are connected when they have at least one common neighboring U-node. Some authors argue that bipartite projections are easier to analyze as compared to their original bipartite network because they are one-mode networks, and hence there is no need to develop new techniques to analyze the bipartite networks. However, because bipartite projections are usually weighted networks, their analysis is not so straightforward. Projecting a bipartite network into a one-mode network merely transforms the problem of the analysis of a bipartite structure into the problem of analyzing a weighted one, not an easy task. Indeed, the projection transformation is associated with loss of information, including the specific identity of the V-nodes responsible for the linkages between U-nodes. Nonetheless, bipartite projection constitutes an important methodological tool in network science and its use is recommended in case where processing a natively one-mode network is impossible or impractical.

In particular, a $|U|$ x $|V|$ biadjacency matrix B, defining a bipartite network $G=(U, V, E)$, can be projected onto an $|U|$ x $|U|$ unipartite (U-projected) or one-mode network, denoted by P_U, as BB^T (the projection on V, denoted as P_V, is similarly obtained by B^TB). The ability to construct unipartite networks from bipartite ones in this way leads also to the question whether the

mathematical properties of the projected networks can be inferred only by knowing the bipartite structure. Several authors have studied the mathematical properties of such projected networks in relation to the properties of the bipartite network. One important feature of the edge weights constructed this way in a projected network is their constrained range of possible values. The range of weight values of an edge between nodes i and j in a bipartite projection (W_{ij}) can be expressed as a function of these nodes' degrees (i.e. k_i and k_j) and the total number of nodes of the other partition ($|U|$):

$$\min\left(k_i,\ k_j\right)\text{-}\left(|U| - \max\left(k_i,\ k_j\right)\right) \ \leq W_{ij} \ \leq \ \min\left(k_i,\ k_j\right)$$

However, in general, higher-degree nodes tend to have stronger edges compared to lower-degree nodes. Additionally, it is widely known that the degree distribution of the nodes in a partition of a bipartite network influences the degree distribution of its one-mode projection on that partition. Moreover, Mukherjee and coworkers have shown that in a projected network the degree distribution of the other partition (V) has also a very strong influence on the degree distribution of the one-mode projection on U [61]. They also showed that if partition U corresponds to a peaked distribution, then it is possible to derive closed-form expressions for the one-mode degree distribution. Other authors went a few steps further in order to calculate the degree distribution analytically [62, 63]. The most complete treatment was given by Nasher and Akutsu [63] who studied the case of scale-free distributions for both sets of nodes (denoted by S-S), and that of scale-free and exponential degree distribution (denoted by S-E) for the two set of nodes. They presented a mathematical analysis demonstrating that it is possible to infer the degree distributions of projected networks given the information contained in the original bipartite network, thereby deriving some simple relationships. For instance, a bipartite network with two sets of nodes with degree distributions $P_U(k) \propto k^{-\gamma 1}$ and $P_V(k) \propto k^{-\gamma 2}$ exhibit a V-projection that follows a power-law $k^{\max(-\gamma 1+1, -\gamma 2)}$ for node degree, where $\gamma 1$ and $\gamma 2$ indicate the power law exponents of the distribution of U and V nodes, respectively, in the bipartite network. On the other hand, a bipartite network with two sets of nodes with degree distributions $P_U(k) \propto k^{-\gamma 1}$ and $P_V(k) \propto \exp(-\lambda k)$ leads to a V-projection, defined by a power-law $k^{-\gamma 1+1}$ node degree distribution. The analytical results were confirmed by computer simulations performed using artificially constructed networks [63].

Various methods of bipartite network projection have been proposed in the literature [17, 33, 64-69] and they all involve the use of a threshold and, in most cases, they yield weighted unipartite networks. Usually, edges the weights of which exceed the threshold value are retained, while those the weights of which are below the threshold value are omitted. The methods greatly vary however on the way threshold values are identified. The simplest and most widespread approach for extracting the backbone of bipartite projections is through the application of an unconditional (or global) threshold. In particular, a single weight threshold is selected and applied to all edges in the bipartite projection and edges are retained in the backbone network only if their weight in the bipartite projection exceeds this predefined threshold. The most commonly used weight threshold of zero preserves all edges with a non-zero weight, whereas others have used different thresholds, including these sets at the percentage of the maximum observed edge weight or at the mean observed edge weight. The unconditional threshold approach, although widely used, suffers from several shortcomings. In general, if the presence of any shared connections to V-nodes is considered adequate for inferring that an edge exists between two U-nodes, then an

unconditional threshold should be used for backbone network extraction. If, however, an instance of shared V-nodes is not sufficient to infer that an edge exists between two U-nodes, then unconditional threshold backbones may be problematic. The structure of a backbone extracted by employing an unconditional threshold depends heavily on the selected threshold value; moreover, certain structural features of unconditional threshold backbones of bipartite networks are systematically biased. Thus, this approach in which a universal threshold is applied indiscriminately to all edge weights can yield a one-mode projection with several undesirable properties [65].

Several methods with thresholds conditioned on the U-nodes' degree are available, and include in the backbone edges the weights of which exceed weight values expected in a null model. All methods begin with a standard projection and then use a statistical model to assess the significance of the weights [70, 71]. Some methods involve normalization of the edge weights in the bipartite projection in a way that adjusts U-nodes' varying numbers of interactions with V-nodes and transforms the edge weights into measures that assess the tendencies or revealed preferences to co-occur. To this end, Bonacich suggested normalization [72], Borgatti used the Pearson correlation coefficient [73], whereas other methods relied on the hypergeometric distribution to perform a test for the statistical significance of edge weights, conditioned on each U-nodes' number of interacting V-nodes (i.e. row marginals in the bipartite network) [65, 74, 75].

Although the aforementioned methods are used for the improvement of unconditional thresholds, they have also been criticized because they implicitly treat V-nodes interchangeably. In such cases, those methods are not suitable for inferring U-nodes' relationships because they fail to consider V-nodes' differing degrees. To overcome the limitations of unconditional and U-nodes' degree conditioned threshold approaches a null model is required to identify the distribution of expected edge weights that would be observed if U-nodes were linked to V-nodes randomly; this linking process is conditioned on (or constrained by) both the U-nodes' and V-nodes' degrees. The most widely used model is the Fixed Degree Sequence Model (FDSM) which compares the observed projection edge weights to the distribution of possible edge weights that might be observed if all U-nodes' and all V-nodes' degrees were fixed at their values in the empirical data. For instance, Zweig and Kauffman presented a systematic approach that evaluates the significance of the *co-occurrence* for each pair of nodes [69]. In principle, the FDSM yields a distribution of expected edge weights that is conditioned on both U-nodes' and V-nodes' degrees; however, in practice, FDSM risks over-conditioning or imposing too many assumptions on the null model. Finally, to address this problem, Neal proposed the Stochastic Degree Sequence Model (SDSM) method that uses a Monte Carlo approach to assess the statistical significance of edge weights against a null model that is conditioned on each U-nodes' number of interacting V-nodes and each V-nodes' number of interacting U-nodes (i.e. both row and column marginals in the bipartite network) [66].

Bipartite biological networks

In this section, a brief description of the most important classes of biological networks that possess a native bipartite structure, as well as the data and the methods pertinent to bipartite biological

networks used is provided. The objectives of the analysis in each case and the specific outcomes obtained from such analyses are outlined. The bipartite networks described below were arbitrarily classified by the authors into four broad categories, namely ecological networks, molecular networks, biomedical networks and epidemiological networks.

Ecological networks

Ecological networks (Figure 5A) are representations of the biotic interactions in ecosystems, in which species are indicated by nodes which are connected by pairwise interactions that can be either trophic or symbiotic. Ecological networks are used to describe and compare the structures of real-world ecosystems. These network models are used to investigate the effects of network structure on properties such as ecosystem stability. A fundamental goal of ecological research is to unravel the mechanisms that influence the stability of fragile ecosystems. Thus, the relationships between ecosystem complexity and stability is a major topic of interest in ecology. The use of ecological networks makes it possible to analyze the effects of the network properties described above on the stability of an ecosystem. Ecological networks can be further subdivided into three broad types: i) food webs (FW), ii) mutualistic webs (MW) and iii) host–parasitoid webs (HPW). Although all three types of network contain trophic interactions, studies of FWs, according to the most strict definition, typically focus on predator–prey interactions where consumers that are usually bigger than their resources are involved [76].

Traditional *food webs* (FW) originate from the population biology school of thought and they focus on trophic links among organisms, particularly predator–prey and primary consumer-basal resource feeding relationships. Historically, research in ecological networks begun from descriptions of trophic relationships in aquatic food webs; however, recent work has explored food webs, as well as webs of mutualists, and, in this way, several important properties of ecological networks have been identified. The energy flux through the web and the relationships between mass and numerical abundance of each species are common themes investigated in food webs. In general, food webs have high complexity, measured as connectance (i.e., the proportion of all possible links that are realized in a network), and smaller size as compared to other biological networks [77]. Of note, food webs can have a native bipartite structure only when two layers are involved (i.e. plants and herbivores), but quite often may consist of ambiguously defined trophic levels connected by a number of links of intraguild predation and thus cannot be viewed as a single bipartite graph. In such cases, in order to perform analyses that rely on the bipartite structure (such as for nestedness), one needs to extract and analyze the bipartite subwebs embedded in them [78].

Host–parasitoid webs also originate from the population biology school of thought but they concentrate on a special type of predator–prey relationships, namely between parasitoids and their hosts [79]. The term "parasitoid" is used to describe insects (usually parasitic wasps) that develop as larvae on the tissues of other arthropods (usually terrestrial insects), which they eventually kill. These networks are particularly well suited for a quantitative analysis because the number of hosts killed and the number of parasitoid individuals produced can be observed directly. Another advantage of these networks is that they are usually resolved at the level of species, avoiding potential problems with the use of ‘trophic species’ of food webs, in which species that share predators and prey are clustered together. An obvious disadvantage of these

networks is that they, by definition, focus on a small subset of the ecological community and are therefore not well suited for studying energy fluxes through the ecosystem [80].

Mutualistic webs are used to study ecosystem properties relevant to pollination and seed dispersal, rather than population dynamics or energy fluxes. Among the various MWs studied in the literature, a significant portion is devoted to pollination networks, which depict the interactions between plants and their animal pollinators [81], frugivore networks, which contain the interactions between plants and their animal seed dispersers, and ant–plant networks, which examine the interactions between plants that provide food and/or shelter for ants, which in turn provide protection for the plants [82]. Specialism tends to be a common feature in most MWs, at least compared to FWs, and this is probably even more the case for endosymbiotic systems. Most networks of plant–animal mutualisms involve a small number of species. An analysis of 52 mutualistic networks showed that their nestedness is high. This pattern suggests a scale-free network. Thus, in mutualistic networks the edges are not placed randomly. Furthermore, species communities with higher complexity (greater number of interactions) nestedness increases with the complexity (number of interactions) of the network, since for a given number of are markedly more nested [83].

Nestedness, as we have already discussed, is considered to be an important topic in the study of ecological networks. A bipartite network, such as the one between plants and their mutualistic animals, is nested if specialists interact with species that form well-defined subsets of the species that generalists also interact with. A nested structure usually implies that there is a core of generalist species interacting among themselves, and a tail of specialists interacting with most of the generalist species [83]. Within FWs, especially in aquatic systems, nestedness appears to be related to body size, because the diets of smaller predators tend to be nested subsets of those of larger predators. There seem to be two extremes, these are freshwater FWs which tend to have many generalists and HPWs which tend to have many specialized parasitoids. The nested structure of mutualistic networks is suggested to play a role in network stability. Additionally, recent analyses have shown that ecological networks are also modular and the modularity co-occurred with nestedness [84]. Moreover, the correlation between nestedness and modularity depends on network connectance [55]. Although mathematical and computational analysis has suggested that nestedness increases species richness as well, an empirical analysis of 59 data sets representing mutualistic plant–pollinator networks showed that this statement may be incorrect. A simpler metric, the number of mutualistic partners of a species, has been found to be a much better predictor of species survival and, hence, community persistence. These results suggested that nestedness is, at best, a secondary factor rather than a causative one for biodiversity in mutualistic communities [85].

The degree distribution of ecological networks is also debatable among scientists. The type of degree distribution (exponential or power-law form) is typically considered indicative of the overall architecture of the network. Early works suggested that the distribution of connections, $P(k)$, is skewed with long tails indicative of power-law scaling. Such features suggest that communities might be self-organized in a non-random fashion that might have important consequences in their resistance to perturbations (such as species removal) [86]. Others have indicated deviations from the "scale-free" topologies, a fact that is thought to result from non-matching biological attributes of species that prevent the occurrence of certain interactions (the so-called "forbidden links"). Large scale analysis for topological patterns in 29 plant–pollinator and

24 plant–frugivore networks showed that most of the plant–animal mutualistic networks show species connectivity distributions following a truncated power-law (broad-scale networks) and only few show scale-free properties. It is suggested that plant–animal mutualistic networks follow a build-up process based on the preferential attachment of species [87]. The skewed degree distributions of bipartite mutualistic and antagonistic networks are usually assumed to show that ecological or co-evolutionary processes constrain the relative numbers of specialists and generalists in the network. Such constraints in adding links, including morphological mismatching between mutualistic partners, restrict the number of interactions established, thereby resulting to deviations from scale-invariance. Other simpler models that do not require the existence of non-matching species traits have also been proposed [88]. Finally, differences have been found between the degree distributions of mutualistic and antagonistic networks, suggesting that different processes are restricting these two classes of networks, especially the largest mutualistic networks. Probably, spatial and temporal heterogeneity largely affect the structure of the larger networks [89].

Of particular note, early ecological networks (e.g., FWs), were binary networks that merely depicted the presence or absence of feeding interactions and not the quantity of those interactions. This lack of quantification has been long recognized as a weakness in ecological network research, since not all species and interactions are equally important, because not all are equally abundant. The availability of quantified webs highlighted the importance of link strength establishing the notion that the strength of the interaction plays an important role in stability, with many weak and few strong links leading to stable but potentially complex webs. More recently, the focus has shifted again from exploring the magnitude of complexity and the strength of interactions to approaches for understanding the specific configuration of complexity (e.g., clustering, the importance of loops or motifs and so on) [76]. All of the above are also verified by the analysis of the trends in establishing new metrics and algorithms suitable for quantitative networks, as well as, the development of new methods for community detection or evaluation of the dynamic properties of the system (see below) [90].

Biomedical networks

Contrary to ecological networks, *biomedical bipartite networks* (Figure 5B) are more abstract since the one partition of the network is usually composed of molecular components found in cells and the other of various indicators of human diseases. In particular, the one partition is usually composed of genes (or their protein products), drugs or environmental exposures; the opposing partition is usually comprised of diseases, symptoms, or drug adverse effects. Thus, biomedical networks have introduced the network analysis techniques into the classical biomedical literature, since they use methods of network analysis in order to model factors that influence human diseases, traditionally analyzed with standard statistical methods. Therefore, this network-based approach in medicine offers a platform to explore not only the molecular complexity of a single disease but also the molecular relationships among distinct pathophenotypes, identify new disease susceptibility genes, uncover the biological significance of disease-associated mutations and identify drug targets and biomarkers for complex diseases [91].

The *gene-disease network* (*diseasome*), the archetype of this type of networks, is a bipartite graph in which the first set of nodes consists of diseases and the opposing one of

disease-associated genes [92]. A disease and a gene are connected by a link only if the gene is implicated in the particular disease. Given a bipartite network, one can construct by projection the human disease network (HDN), the network of human diseases, where diseases with common genetic components are connected, or the human disease gene network (HDGN), the network of human genes, where genes participating in common human disorders are connected. The first diseasome was created based on the list of human disorders, disease genes and associations between them obtained from the OMIM database by Goh and coworkers [93]. However, although OMIM is one of the major repositories holding genetic association data for Mendelian diseases, it mainly archives rare disorders of high penetrance [94]. This parameter is of importance, since multigenic diseases of low penetrance may have different properties which have to be taken into account. Other subsequent studies, such as the ones conducted by Barrenas *et al* [95] and Liu *et al* [96], partially overcome this issue by integrating gene-disease association data from multiple resources. Of importance, in the study conducted by Goh *et al*, neither the disease concepts not the gene terms were standardized, whereas, in the studies conducted by Barrenas *et al* [95] and Liu *et al* [96], an effort was made to homogenize the disease concepts but not the gene terms. When data from GWAS are used, one is also able to construct a *gene-phenotype network* linking genetic polymorphisms to intermediate phenotypes such as cholesterol levels or blood pressure [97]. Such approaches can be useful, especially in the context of identifying the causal pathways linking genetic variation, intermediate phenotypes and diseases (Mendelian Randomization), but the data on phenotypes are rather sparse. Recently, Kontou *et al*. [15], performed a similar analysis by combining data from OMIM and from two other primary resources containing information of gene-disease associations: The NIH’s Genetic Association Database (GAD) [98], which contains data from genetic association studies which mostly target multigenic diseases of low penetrance, and the National Human Genome Research Institute (NHGRI) Catalog of Published Genome-Wide Association Studies (GWAS) [99], which includes a manually curated collection of published GWAS, with more than 100,000 assayed SNPs and SNP-disease associations. GAD and GWAS are, therefore, complementary to OMIM. Moreover, since disease name heterogeneity and ambiguity in all three repositories would not allow for a direct data comparison, the naming conventions described in the International Classification of Diseases (ICD) were used. Finally, in order to maintain a uniform nomenclature, all genes from the three databases were converted to the official HGNC (HUGO Gene Nomenclature Committee) [100] gene symbols.

Such network-based approaches for the discovery of gene-disease associations have enabled biomedical researchers to not only investigate the genetic complexity of a particular disease, but also the relatedness among apparently discrete disease phenotypes [91, 101]. Diseases are found to be highly connected genetically, displaying many connections between both individual disorders and disorder classes. In other words, it seems to be a widespread genetic relatedness across many diverse domains of human disorders, transcending traditional disease categorization. Moreover, disease networks can provide the foundation for predicting causative genes, thereby unravelling a disease’s underlying molecular mechanisms and enabling the design of new therapeutic strategies [91, 101]. Genes associated with similar disease phenotypes have a higher propensity to interact physically with each other, forming distinct disease-specific functional modules [102, 103]. Connections between disorders are also not completely random. Disorders rather tend to form clusters on the basis of similar pathophysiology.

Conversely, diseases with similar phenotypes have an increased tendency to share genes [93]. To achieve this global connectivity, complex diseases, such as diabetes and obesity, play the role of ‘connectors’, bridging in this way disorders from different classes. Finally, of particular note, some genes are associated with only few diseases, whereas others are implicated in numerous diseases, and likewise, some diseases are influenced by only 1-2 genes, whereas others are caused by dozens of genes [92].

Gene-disease networks have also been constructed for various classes of related diseases, including autoimmune diseases [104], neurological diseases [105],cardiovascular diseases [106] and others. Tissue specificity is also considered in gene-disease networks, since clinical manifestations of diseases are usually restricted to specific tissues. Although some disease-associated genes are expressed only in certain tissues, the expression patterns of disease genes alone cannot explain the observed tissue specificity of diseases. By extending the diseasome, a network-based approach was used by Hayasaka and colleagues to investigate how different brain areas are associated to genetic disorders and genes. In particular, the authors constructed a tripartite network with genes, diseases, and the affected brain areas. In the resulting network, a disproportionately large number of gene-disease and disease-brain associations were attributed to a small subset of genes, diseases, and brain areas. Furthermore, a small number of brain areas were found to be associated with a large number of the same genes and diseases. These core brain regions encompassed the areas identified by previous genome-wide association studies, and suggest potential areas of focus for the future imaging genetics research [107]. These ideas were implemented in the so-called *disease-tissue network*, which is an obvious extension of the diseasome, in order to include information regarding tissue specificity. The primary hypothesis here is that for a disease to manifest itself in a particular tissue, a whole functional subnetwork of genes (disease module) needs to be expressed in that tissue. The expression patterns of disease genes were combined with the human interactome and the results indicated that genes expressed in a specific tissue tend to be localized in the same neighborhood of the interactome. On the contrary, genes expressed in different tissues are segregated in distinct network neighborhoods. Most importantly, Kitsak *et al*. showed that it is the integrity and the completeness of the expression of the disease module that determines disease manifestation in selected tissues. This approach led to the construction of a *disease-tissue network* that offers a predictive map of the statistically significant disease-tissue associations. This approach allowed the researchers to examine known disease-tissue relationships and predict newly definable disease-tissue associations [108].

Further extending diseasome, a large-scale biomedical literature database (including PubMed and NCBI’s MeSH terms) was used to construct a *symptoms-disease network* (Human Symptoms Disease Network, HSDN) and investigate the connection between clinical manifestations of diseases and their underlying molecular interactions [109]. In the projected network, the link weight of two diseases quantifies the degree of similarity of their respective symptoms. The authors integrated disease–gene association and PPI data, and found that the symptom-based similarity of two diseases correlates strongly with the number of shared genetic associations and the extent to which their associated proteins interact. Moreover, the diversity of the clinical manifestations of a given disease can be related to the connectivity patterns of the underlying PPI network. Such approaches could be useful in the identification of unexpected associations between diseases, in the disease etiology research or in drug design.

Another important extension of the diseasome is based on the identification of environmental factors that influence diseases. The majority of diseases (especially the polygenic ones) are, in part, caused or influenced by the human interaction with harmful substances of the environment. Traditionally, epidemiological studies have been investigating such exposures, whereas the identification of gene-environment interactions represents an important area of genetic epidemiology [110]. The *exposure-disease network* was compiled using a global repository of CDC (Center for Disease Control and Prevention), which contains literature surveys on matching environmental chemical substances exposure with human disorders. The bipartite network contained links from 60 substances to over 150 disease phenotypes. The analysis of the bipartite network and the projected networks identified mercury, lead and cadmium to be associated with the largest number of disorders. On the other hand, breast cancer, fetal abnormalities and non-Hodgkin's lymphoma were found to be associated with most of the environmental chemicals. Moreover, tobacco smoke compounds, parabens and heavy metals tend to be connected, implying common disease causing factors; however, this is not the case for fungicides and phyto estrogens [111].

Furthermore, the diseasome was extended to include drugs. The *drugs-target network* (*drugome*) consists of a bipartite graph that links approved drugs with the their target proteins (the gene products) [112]. The network produced in this way connects most drugs into a highly interlinked giant component, with strong local clustering of similar drugs. Topological analyses of this network quantitatively showed an overabundance of drugs that target already targeted proteins, confirming the prevalence of the so-called 'me-too' drugs on the market. To analyze the relationships between drug targets and disease gene products, the shortest distance between both sets of proteins was measured in models of the human interactome network. Although an enrichment for etiological drugs, which directly target the disease-causing component, was clearly observed, still a majority of existing drugs target components as far away from the disease-causing genes as a random target would do, suggesting a predominance of palliative-acting drugs. Finally, a significant shift towards the closer-to-target drugs approved after 1996 from those approved before 1996 was observed, supporting a recent trend towards rational drug design [112]. The diseasome can be further supplemented by drugome. Traditionally, new targets for drugs have been predicted on the basis of molecular or cellular features, by exploiting, for example, similarity in drug chemical structure or activity across cell lines. A network-based inference method based on the drug-target bipartite network topology similarity enabled, however, the prediction of new targets for existing drugs, thereby outperforming both drug-based similarity inference and target-based similarity inference methods. By using this method, five old drugs (montelukast, diclofenac, simvastatin, ketoconazole, and itraconazole) were found to have polypharmacological effects on human estrogen receptors or dipeptidyl peptidase-IV, whereas simvastatin and ketoconazole showed potent antiproliferative activities on breast cancer cell lines [113].

In a fashion similar to the drug-disease network, the *vaccine-disease* and the *vaccine-gene* networks were constructed by Zhang and coworkers [114]. From these networks, those genes that interact with many vaccines and, conversely, those vaccines associated with many genes were identified as hubs. These findings correlate with existing knowledge and generated new hypotheses in the fundamental interaction mechanisms involving vaccines, diseases and genes. Similar approaches were based on phenotypic side-effect similarities (the *drug-side effects*

network), in order to infer whether two drugs share a target. Campillos and colleagues [115] tested several of such unexpected drug-drug relationships on 746 marketed drugs, validated the implied drug-target relations by *in vitro* binding assays, and found 11 drugs that exhibited significant activity. Nine of those were tested and confirmed in cell assays, documenting the feasibility of using phenotypic information to infer molecular interactions and hinting at new uses of marketed drugs [115]. Going one step further, a multi-level network (the *process-drug-side effect network*) was built by merging the *drug-biological process network* and the *drug-side effect network*. By analyzing the process-drug-side effect network, meaningful relationships between biological processes and side effects were inferred in an efficient manner [116].

Biomolecular networks

Bipartite graphs provide an appropriate abstraction to represent relationships and associations between different classes of biological molecules and therefore have been extensively used for studying and modelling interactions between biomolecules (Figure 5C). Unlike biomedical networks, which represent relationships between abstract terms such as diseases or phenotypes, molecular networks illustrate interactions that occur physically between biomolecules and take place inside all various cell compartments. These interactions are reconstructed by employing computational and mathematic methods of analysis applied on multi-omics data generated from high-throughput experiments.

Data from high-throughput proteomics experiments (i.e. Y2H, IP-MS and TAP-MS) are extensively modeled using bipartite graphs [117-119]. Bipartite graph models are utilized in different levels of analysis of PPI data, including assignment of individual peptides to proteins, as well as analysis and detection of protein complexes.

Peptide-to-protein assignment modelling by bipartite graphs. Any type of high-throughput proteomics experiment that employs mass spectrometry (MS) reports a list of all the detected peptides and a measure of their abundance. The subsequent analysis requires the assignment of each identified peptide to the corresponding protein and an estimate of its abundance. Therefore, as many peptides are assigned to a single protein and many proteins share the same peptides, a bipartite graph between peptides and proteins is constructed to carry out the analysis. This network between peptides and proteins is then processed by employing a series of algorithms, which operate on this bipartite structure to find the most appropriate protein assignment for each peptide. Inferring the correct proteins from these complex bipartite graphs is a difficult problem and, therefore, methods based on empirical Bayesian analysis, reverse database search and calculation of expectation values have been developed. Even though protein identification is the most widely used method for the analyses of MS-based proteomics data, the available software tools for identifying proteins are still not perfect [120]. [121] A detailed review of available protein identification methods is provided by Nesvizhskii [122].

Protein complexes in protein-protein interaction (PPI). Modeling protein complexes as networks plays the most important role in advancing our understanding of protein functions and elucidating the dynamics of cellular super-molecular organization. However, protein interaction data generated by high-throughput experiments such as yeast-two-hybrid (Y2H) and tandem affinity-purification/mass-spectrometry (TAP-MS) are challenged by the presence of high number of false positives and high false discovery rates [123]. Similar to the peptide-protein bipartite

network, co-complex relations of proteins participating in different complexes are modelled as bipartite graphs in TAP-MS experiments. Here, each individual protein is included in one set of nodes and the set of complexes in which it participates in comprises the second set of nodes. In recent years there has been a growing number of efforts to incorporate inter-domain knowledge to support large-scale analysis of PPI networks. A representative study incorporates GO semantic terms and topological features of the baits and prey proteins to calculate pairwise similarities of baits and generate “seeds” of clusters. Then each seed cluster is extended to recruit prey proteins that are significantly associated with the same GO terms. Then network clique and motifs algorithms are applied to identify the protein complexes [117]. An additional typical technique, employs network community structure detection algorithms together with two well established machine learning algorithms to predict the protein-complex bipartite network in *Saccharomyces cerevisiae*. Communities were detected by a modularity detection algorithm and the community assisted method has outperformed a neighboring assisting method [124]. A more recent approach [125] involves a method inspired from spectral analysis, where the network power graph analysis is applied for the identification of complete bi-clique motifs. These motifs corresponded well to protein complexes and a revisit of a characteristic study led to the prediction of the catalytic and regulatory subunits of the casein kinase II complex, as well as the untangling and identification of new protein interactions in the nucleosome.

Gene regulatory networks and *gene co-expression networks* realized by the physical interaction (binding) of TFs to the regulatory regions of target genes, can readily be modelled as bipartite graphs, where one layer of nodes represents the regulatory genes and a second layer of nodes represents target genes. Consequently, every edge in the graph represents a regulatory relation in the form of binding of each regulatory gene product (encoded by the regulatory gene) to the regulatory region of the target gene. Moreover, the respective weights associated with each regulatory edge may represent the influence or the interaction strength between a TF and the regulated gene. An important property of network connectivity, that is, versatility, emerges when bipartite graphs are used to describe data derived from transcriptomics experiments. In the process of discovering the simplest (sparser) bipartite network able to describe the data [126], a biologically meaningful distinction between versatile and non-versatile networks was made. Versatile networks can describe any type of data and thus are indistinguishable from one another, whereas non-versatile ones require a limited set of data due to constrains imposed the data described. This limitation however can be utilized to the reconstruction of network topologies and regulatory signals and get a glimpse into the biological meaning of the regulatory interactions.

Modeling gene transcription regulation by bipartite graphs facilitated the development of network reconstruction methods involving the decomposition of the gene expression matrix. Typically, a matrix of dimensionality *NxM* (*N* genes and *M* samples) is broken down to regulatory signals and regulatory strengths. Established matrix decomposition methods, such as principal component analysis (PCA), independent component analysis (ICA) and singular value decomposition (SVD) have been applied to reduce the dimensionality of the gene expression matrix and, therefore, reconstruct regulatory interactions. However, all PCA, ICA and SVD based methods are using statistical assumptions such as orthogonality and statistical independence, and perform decompositions which are difficult to be interpreted in bio-molecular systems. Nevertheless, the bipartite network representation of GRNs permitted the development of a family of methods termed Network Component Analysis (NCA), first introduced in [127]. NCA-based

methods are able, under certain constrains, to find scaled reconstructions of the gene expression matrix in two matrices |*A*| and |*P*|, where |*A*| (an *NxL* matrix) contains the regulatory strengths of *L* regulatory genes on the *N* regulated genes and |*P*| (an *LxM* matrix) contains the regulatory signals of *L* regulators in *M* conditions. The criteria that have to be met in order for NCA to be able to perform matrix decomposition include: full rank of the matrix |*A*| (full column rank of matrix |*A*| must also be maintained even after the removal of a regulatory node, which implies that each column of |*A*| must have at least *L-1* zeros) and full row rank of matrix |*P*|. Modeling of GRNs as bipartite graphs and their decomposition with NCA has been extensively applied, as NCA's criteria are easily fulfilled by a broad spectrum of biological systems. For a comprehensive review on the different algorithmic approaches and the different biological applications of the NCA-based methods on biological systems the reader can further refer to [128].

In an effort to extend NCA, Ye and coworkers incorporated genetic variation data in the form of single nucleotide polymorphisms (SNPs), together with gene expression and ChIP-Chip data for the concentrations and binding site affinities of TFs, in a framework that predicted accurately trans- and cis- acting SNPs. Here the trans-acting SNPs correspond to the products of regulatory genes and cis-acting ones to the binding sites of these products in the target genes of the bipartite network. [129].

Furthermore, a recent method named CONDOR [130] uses the modular structure of the bipartite graph to associate SNPs with genes' functions. In brief, this method utilizes the community structure of the bipartite graph (i.e., hubs and local clusters) in order to associate expression quantitative traits loci (eQTLs) with the genomic context. The context here is defined not only in terms of genes in the immediate proximity of significant genetic variants, but also in terms of the functionally implicated genes through the bipartite network structure analysis. The method exploits genome wide eQTL analysis in a way that is not restricted to the immediately neighbors of the eQTL-SNP gene.

Furthermore, bipartite network analysis of transcription regulation has been applied to comparative studies of GRNs [131]. In this study, the projected networks of transcription factors and regulated genes (RGs) from *E. coli* and *S. cerevisiae* have been compared to find common characteristics and differences. The connectivity patterns of these two networks were found to be very similar. To better understand the differences, randomized versions of the original networks have been constructed. The difference of the TFs to RGs ratios among species has been found to be the most significant, highlighting a major organizational difference in transcription regulation between prokaryotes and eukaryotes.

Other gene expression regulation networks

The increasing availability and decreasing price of the high-throughput experiments resulted to the generation of an overgrowing number of datasets that involve different types of biological entities including TFs, mRNAs, proteins, regulatory sequences (enhancers, repressors etc.). Bipartite graphs provide a suitable structure to model and analyze this wealth of data. In a network topology analysis work, the SICORE algorithm has been proposed [132] for the identification of regulation as well as co-regulation effects using protein arrays, miRNAs and gene expression. In a similar multi-data integrative method, data from the human protein interaction network were combined with those from the transcription regulatory network to characterize co-

regulatory modules [133]. The method entailed a probabilistic statistical model which evaluated whether a cluster of co-regulated proteins is likely to form a transcriptional regulatory module in an integrated network.

A novel class of non-coding RNAs has been recently discovered, the long non-coding RNAs (lncRNAs), more than 200 nucleotides in length, a feature that sets them apart from the other small regulatory RNAs. Evidently, bipartite graphs provide a suitable model to study the structural roles of lncRNAs, as one layer of nodes can represent the lncRNAs and the second layer the proteins they interact. A method termed lncRNA–protein bipartite network inference (LPBNI) has been developed recently [134] which is proposed to be the first one which allows the construction of such networks. The method relies on the extraction of characterized lncRNAs-protein interactions from online databases and the usage of a propagation technique to assign each protein a score that is specific for each lncRNA, thereby providing a full set of ranked lists of interacting proteins for every lncRNA.

The bipartite metabolite-reaction representation of the metabolism is a reliable model to represent *metabolic networks* and metabolomics data, where data can be assigned separately with one layer of nodes representing the metabolites and the second layer representing the reactions. This representation avoids most of the erroneous assignments of isozymes as well as multi-functional enzymes. A tool introducing active module analysis of metabolic bipartite networks (AMBIENT) has been proposed as an effective means to analyze high-throughput data in a metabolic context [135]. Moreover, in a biomedical study [136] bipartite KEGG *pathways-gene networks* have been investigated together with the detection of differentially expressed genes (DEGs) from microarray experiments. The approach comprised a machine learning method that combines classification from both DEGs derived networks and bipartite KEGG pathways. The generated model was then applied to a series of cancer datasets and was able to robustly reduce the frequently high number of false positives occuring in single DEG experiments.

To summarize, bipartite networks are invaluable in modeling and studying biomolecular networks for two major reasons. Firstly, they provide a straightforward abstraction as the two different layers of nodes correspond directly to two different sets of biomolecular entities with distinct properties. Secondly, there are several powerful analytical methods from graph theory and linear algebra which -by taking into account particular types of bipartite network connectivity-provide solutions to the data representation and complexity reconstruction problems of the mutli-omics, high dimensional, high throughput biological data. This section reviewed the most commonly used methods and examples but it remains important to highlight a unifying method applicable to all bipartite biomolecular graphs, the power graph analysis. Power graphs are topological transformations of biomolecular networks into less redundant representations. This is achieved by exploiting the abundance of bicliques as topological motifs which are elementary, essential and embedded in the structure of biological networks. Power graph analysis is an analytical tool that can easily be generalized and applied to directed, undirected and bipartite networks [137] but always returns a bipartite graph that describes a complex, “hairy ball” like network by bipartite structures. Power graph analysis for the identification of protein complexes has notable application in the analysis of bipartite GRNs. Moreover, power graph analysis allows the decomposition of a bipartite network into a union of significant motifs such as the star motif, the clique motifs and bicliques [125]. This decomposition was used to discover a hierarchy of clusters of transcription factors linked to a hierarchy of clusters of target genes, thereby permitting

the reproduction of the results of a laborious combined experimental and computational previous study [138] where only the bipartite network structure of the transcription regulatory network in yeast was used as input.

Epidemiological Networks

Another distinct type of bipartite networks, as far as the type of data analyzed and the goals of the analysis are concerned, are those that are directly related to epidemiology (Figure 5D). These networks share some features with the biomedical networks, with the focus on human diseases being the most important, but the main difference lies in the fact that the data are collected and analyzed in an individual patient's basis. In general, network analysis in public health and epidemiology resembles the classical approach of social networks analysis, and has been used mainly to study disease transmission, especially for HIV infections/AIDS and other sexually transmitted diseases (STDs) [139]. Bipartite structures can be built based on individuals that are classified by gender, location, infectious agent or comorbidities.

In one case, the *sexual contact network* can be represented as a bipartite graph, in which males form one part of the graph and females the other [140]. Such approaches can be valuable in the understanding of sexual behavior and the evolution of intimate relationships over time [141], as well as the modeling and simulation of STDs, especially HIV infections/AIDS [142-144]. Other theoretical studies have shown that, apart from the dependence between the epidemic threshold and the average and variance of the degree distribution of the network, there is a cut-off value for the infectivity of each population, below which no epidemic outbreak can occur, regardless of the value of the infectivity of the other population [145].

Vector-borne diseases, for which transmission occurs exclusively between vectors and hosts, can also be modeled as bipartite networks. In such models, theoretical work suggests that the spreading of the disease strongly depends on the degree distribution of the two sets of nodes and it is sufficient one of the sets to have a scale-free degree distribution with a slow enough decay for the network to have an asymptotically vanishing epidemic threshold [146].

Another case in which the bipartite network can model the spreading of a disease is when the one set of nodes consists of geographic locations (clusters) in which the epidemic occurred, and the second set of the infected cases within a given time period. In this network, which is analyzed by projection, two locations are associated if they are both connected to common infected cases in the same period, and the number of infected cases is considered as the weight of the links [147].

Finally, *comorbidities network* is a prominent example of epidemiological networks. Comorbidities, that is, the co-occurrence of diseases, can provide valuable information regarding the underlying biological mechanisms of multifactorial diseases and help to elucidate the effects of environmental exposures, such as diet, lifestyle and medication on diseases. By linking network dynamics to real-life data, patient data could provide a valuable basis for generating hypotheses concerning the mechanisms of disease and prove useful in drug repurposing and the development of targeted therapeutic strategies [148]. However, this type of information is conceptually different from the one encountered previously in biomedical networks, since it needs individual patients' data (IPD) in order to be compiled. In particular, detailed information from each patient is needed and the adjacency matrix of the generated bipartite network resembles closely

the traditional epidemiological datasets (the rows represent the patients and the columns the diseases). Projection of this bipartite network can also result to a unipartite network with the correlations of various comorbidities, the so-called *Phenotypic Disease Network* (PDN) [149]. Large datasets of this type, which could be useful for network analysis, are difficult to be found in general. However, since the worldwide health transaction data are now often collected electronically, disease co-occurrences are currently analyzed quantitatively [150] and in some cases these data cover even entire nations [151]. In the most notable example of PDN, more than 30 million patients' electronic health records compiled from Medicare claims were used. By analyzing the co-occurrence of diseases and the mortality, they found that disease progression can be studied using network methods, offering in this way the opportunity to enhance our understanding of the origin and evolution of human diseases. Additionally, the dataset that was made publicly available represents the largest relational phenotypic resource that is publicly available to the research community [149]. Such data [152] can be used in other analytical techniques which are in use in traditional epidemiology (e.g., in meta-analysis of summary data). Other network analyses, such as analyses of co-morbidities of hip-fracture (HFx) elderly patients, provided unexpected results that would be difficult to obtain otherwise, since patients with more serious comorbidities seem to have better follow-up that reduces the risk of readmission, whereas those with relatively less-serious specific comorbidities may have less stringent follow-up, leading to unanticipated incidents that precipitate readmission [153].

Models and algorithms for bipartite graphs

In this section, some general related problems in bipartite graphs and the problem-solution algorithms are first described. Then, some important properties of bipartite graphs that arise from viewing them as dynamic systems, such as percolation and controllability, are discussed.

Odd cycle transversal

A graph $G = (V, E)$ and a number k are given. Does there exist a set of at most k vertices the removal of which from G would cause the resulting graph to be bipartite? The problem is NP-complete [154], i.e., there is no algorithm that can solve it within a polynomial time with respect to the size of the input, unless $P=NP$. The problem is fixed-parameter tractable, that is, there is an algorithm the running time of which can be bounded by a polynomial function of the size of the graph multiplied by an exponential function of k [155]. More specifically, the time for this algorithm is $O(3^k |E||V|)$ [156]. The name *odd cycle transversal* is attributed to the fact that a graph is considered as bipartite if and only if it has no odd cycles. Hence, deleting vertices from a graph in order to obtain a bipartite graph, one needs to "hit all odd cycle" or find a so-called odd cycle transversal set.

Edge bipartization

In a given graph $G = (V, E)$, it is possible to delete at most k edges so that the graph remains bipartite. This problem is also NP-complete and fixed-parameter tractable, and it can be solved in time $O(2^k |E|^2)$ [157].

Matching

A *matching* in a graph is a subset of its edges, where no two edges share an endpoint. In many cases, it is simpler to find a specific matching in bipartite graphs than in arbitrary graphs. A matching in a bipartite graph is called *perfect* if for every node of the graph there is an edge in the matching. Given a matching *M*, if *M*+*e* is not a match for any edge *e*, then *M* is called *maximal matching*. A matching consisting of a maximum number of edges is called *maximum matching*. While a maximum matching is maximal, a maximal matching is not necessarily maximum. A maximal matching can be easily found by a greedy algorithm in any graph, while a maximum matching in a bipartite graph can be found in $O(\sqrt{(|V|)}|E|)$ time using the Hopcroft-Karp algorithm [158]. In weighted bipartite graphs, a *Maximum Weight Matching* can be found within $O(|V|^2|E|)$ time using the *Hungarian* algorithm [159]. While the largest cardinality maximal matching (i.e., a maximum matching) can be found within polynomial time, a *minimum maximal matching* cannot be found in polynomial time unless *P*=*NP*. However, the number of edges in any maximal matching is at most twice the number of edges of the minimum maximal matching, and therefore the minimum maximal matching can be approximated within a factor of two in polynomial time. Although a perfect matching can be easily found in bipartite graphs by finding a maximum matching, counting the number of different perfect matchings in a bipartite graph appears to be very difficult. In fact, this problem is #*P*-complete, that is, if there is a polynomial algorithm which solves it, then *P*=*NP* [160].*Stable Marriage*

The *Stable Marriage problem* refers to an interesting problem related to bipartite graphs, which may have applications in biology. Let *M* and *W* be two sets of men and women, respectively, with $|M|=|W|=n$. Each man *m* in *M* has a preference $p_m(m, w)$ for each woman *w* in *W* and, conversely, each woman *w* in *W* has a preference $p_w(w, m)$ for each man *m* in *M*, so that:

- for all *m*,*w*: $1 \leq p_m(m, w) \leq n$
- for all *m* and any $w_1 \neq w_2$: $p_m(m, w_1) \neq p_m(m, w_2)$
- for all *w*,*m*: $1 \leq p_w(w, m) \leq n$
- for all *w* and any $m_1 \neq m_2$: $p_w(w, m_1) \neq p_w(\mathrm{w}, m_2)$

In other words, each man (or woman) has a list of distinct preferences for each woman (or man). If $p_m(m, w) =1$, then *m* first prefers *w*, while if $p_m(m, w) =2$, then *w* is the second choice of *m*, and so on. The goal of the problem is to find *n* marriages between men and women so that every marriage is stable. A marriage (*m*, *w*) is not stable if and only if there is another married couple (*m*', *w*') so that $p_m(m, w) > p_m(m, w')$ and $p_w(w', m') > p_w(w', m)$. In other words, a marriage (*m*, *w*) is not stable if there is another married couple (*m'*, *w'*), where *m* prefers *w'* than his wife and *w'* prefers *m* than her husband.

More formally, in a stable marriage problem, given a complete bipartite graph *G*(*V*, *U*, *E*), where $|V|=|U|=n$ and each edge (v,*u*), where *v* (resp. *u*) belongs to *V* (resp. *U*), have been assigned two values: a value $p_m(v, u)$ and a value $p_w(u, v)$, defined as above. The question is whether there is a stable perfect matching in *G* that represents *n* stable marriages (defined as above) between the sets *V*, *U*. Of note, there are *n*! different perfect matchings in *G*. The answer is that there is a fast algorithm [161] which always returns a stable perfect matching. The algorithm is as follows:

- In the case there is an unmarried woman
    - Each unmarried woman proposes to the man that prefers that most among those that have not already rejected her

- Each man selects the woman who prefers the best among the women that proposed to him and rejects the rest proposals

It has been proven that the above algorithm always returns stable marriages for all men and women within a O(n^2) number of proposals, where *n* is the number of men (or women). A variant of the problem where the order of preferences is not strict, that is, there are men (or women) that equally prefer other women (or men), has also been studied [162]. For more information regarding the problem and its variants refer to a survey conducted by Iwama and Miyazaki [163].

Other general problems

Finding *the longest path* (i.e., finding a simple path of a maximum length) is NP-complete in bipartite graphs, in contrast to the shortest path which can be solved in polynomial time on any arbitrary graph. Moreover, the *girth of a graph* is defined as the length of the shortest cycle contained in the graph. Since bipartite graphs may contain only even cycles, the girth of a bipartite graph is an even number (or 0). Given a bipartite graph $G(V, U, E)$ of girth g, there is an algorithm for counting the number of cycles of length g, $g+2$, $g+4$, within $O(gn^3)$ time, where $n=max(|U|, |V|)$ [164]. In the *k-path partition* problem the task is to partition a given graph *G* into the minimum number of paths, each of which has a length at most *k*. In bipartite graphs, the *k-path partition* problem is usually NP-complete in, while polynomial-time algorithms are known for specific families of bipartite graphs [165]. Furthermore, given a bipartite graph *G*, a *biclique* of *G* is a subgraph of *G* which is also a complete bipartite graph. Finding a *biclique* of a maximum number of vertices can be done in polynomial time [166], while finding a *biclique* of a maximum number of edges is NP-complete [167]. There is also a large body of literature on the methods for the optimal drawing of bipartite graphs [168-174]. An important algorithmic problem that arises in this respect is drawing a bipartite graph in a way that minimizes crossing edges [175-177].

Percolation

Recent work on network theory has addressed the problem of resilience of networks by the random or targeted deletions of nodes or edges. From the perspective of Statistical Physics, "*percolation* is the simplest process showing a continuous phase transition." Percolation models on random bipartite graphs offer a simple illustration of this process. Percolation has been examined on graphs with a general degree distribution and has given accurate solutions to various cases, including bond percolation, site percolation, and models in which occupation probabilities depend on the degrees of the vertices [178]. From this point of view, the failure of a biomedical network could be considered as a percolation process and the determination of the cut-off number of failed nodes/edges required to break down the whole network could be a particularly useful criterion for network failure. [179]. Percolation has been studied mainly in unipartite graphs, but recently the process has been described also in bipartite graphs [180]. In the particular model, throughout the percolation process, the links between nodes with degrees *k* and *q* are preserved with a probability proportional to $(kq)^{-\alpha}$, where α is positive so edges between hubs have greater probability to fail. The entire node/edge removal process was studied by using a theory of generating functions, and equations for the macroscopic description of the system were deduced.

Link Prediction

The problem of *link prediction* refers to seeking a function of two vertices that denotes the similarity or proximity of the vertices. Link prediction enhances our understanding on the associations between nodes in bipartite networks. In general, there are several algorithms that can be used to extract missing information, identify spurious interactions, evaluate network evolving mechanisms, and so on [181]. However, common link prediction functions for general (e.g., unipartite) graphs are defined using paths of length two between two nodes. Since in a bipartite graph, adjacency vertices can only be connected by paths of odd lengths, these functions are not applicable. Instead, a certain class of graph kernels (spectral transformation kernels) can be generalized to bipartite graphs, where the positive-semidefinite kernel constraint is relaxed by using the odd component of the underlying spectral transformation [182]. Other methods have also been developed, including those based on machine learning [183] or those that make use of the concept of internal links [184].

Graph Ranking

Consider an edge-weighted graph G=(V, E) where each edge of G has been assigned a positive real number. A set of preferences or order relationships among nodes of G is also given. This set is usually represented as a possibly directed weighted graph G'=(V, E'), where $E' \subseteq E$. Each edge of E' has been assigned a positive real number with the following interpretation: If (u, v) $\in E'$, then u must be ranked higher than v, and the penalty for mis-ordering such a pair is given by the weight of edge (u, v) of G'. The goal is to rank the nodes of G so that to minimize the ranking error [185]. Ranking is a general problem in graphs of arbitrary structure, but the special structure imposed by the bipartite nature triggered the development of specialized algorithms [186]. Moreover, regularization-based algorithms have appeared, which find ranking functions that minimize regularized versions of the ranking error [187]

k-partite graphs

As we have already mentioned in the beginning of Section ‘Bipartite Graphs’, a bipartite graph is a special case of a k-partite (or multipartite) graph for k=2. More formally, a k-partite graph consists of k non-empty and disjoint sets of nodes U_1, …, U_k where a node $u \in U_i$ can share an edge with a node $v \in U_j$ only if $i \neq j$. In other words any edge of the graph can only connect nodes in different sets (i.e., node sets U_i are independent). Bipartite and tripartite (i.e., for k=3) graphs are probably the most studied families of k-partite graphs. Let us now list some interesting problems related to k-partite graphs. The problem of recognizing whether an arbitrary graph is k-partite is equivalent to the problem of deciding whether the nodes of the graph can be colored using at most k colors so that each node has been assigned one color and any two adjacent nodes have been assigned different colors. While recognizing a bipartite graph can be easily done in polynomial time, recognizing a k-partite graph for any $k > 2$ is NP-complete. However recognizing a complete k-partite graph (i.e., a k-partite graph where any two nodes in different node sets share an edge) can be done within polynomial time for any k. In fact, many problems which are NP-complete in arbitrary graphs and sometimes even in k-partite graphs can be solved within polynomial time in complete k-partite graphs, as for example the Maximum Clique problem, the Maximum Independent Set problem, the Graph Isomorphism problem, the Hamiltonian Cycle problem, etc. A nice recent work on multipartite graphs with devoted sections to the applications of such graphs in biology, can be found in [188, 189]

Community Detection

A network is considered to have *community structure*, or clustering, if the nodes of the network can be grouped into (potentially overlapping) sets of nodes in a way that each set of nodes is densely connected internally, that is, having many edges joining nodes of the same cluster and comparatively few edges joining nodes of different clusters. In the case of *non-overlapping* community detection, the network is divided naturally into groups of nodes with dense connections internally and sparser connections between groups. There are plenty of methods available for detecting communities, ranging from traditional clustering methods (hierarchical, spectral etc.) to divisive algorithms and methods that maximize the criterion of modularity [190]. In the case of bipartite networks, community detection has also received considerable attention [191]. It is well understood that community detection is related to the modularity of a network, which quantifies the extent to which vertices cluster into community groups, relatively to a null model network [192]. Moreover, research on the community structure in bipartite graphs has yielded new metrics for the clustering coefficient [44, 193] and several specialized methods have been proposed for community detection [194-196] including algorithms for overlapping communities [197] as well as for quantitative biadjacency matrices [198]. A conceptually similar condition encountered mainly in gene expression studies is biclustering (also referred to as coclustering in the literature). Biclustering consists of simultaneous partitioning of the set of samples and the set of their attributes (usually gene expression) into classes. Samples and genes classified together are supposed to have a high relevance to each other. The goal is to find submatrices, where the genes exhibit highly correlated activities for every condition. The various biclustering methods for gene expression data are reviewed in Busygin *et al*. [199, 200]. In general, biclustering methods is thought/presumed to have several advantages over conventional hierarchical clustering approaches and there are also considerable performance differences between the two methods. Thus, it would be interesting to test the application of biclustering methods in the task of community detection in bipartite graphs.

Controllability

Controllability describes the ability to drive a dynamic system (e.g., a network) from an initial state to a desired final state in finite time, with a suitable choice of inputs. The controllability of general directed and weighted complex networks has recently been the subject of intense study by several research groups. Investigation of the controllability of complex networks has led to the identification of the set of driver nodes with time-dependent control that can guide/drive the system's entire dynamics. Applications in several real networks revealed that the number of driver nodes is determined mainly by the network's degree distribution. Sparse heterogeneous networks are the most difficult to control, but dense and homogeneous networks can be controlled by using only a few driver nodes. Counterintuitively, the driver nodes tend to avoid the high-degree nodes [201]. Furthermore, an analytical framework to address the controllability of bipartite networks is based on the dominating set (DS)-based approach which identifies the topologies that are relatively easy to control with the minimum number of driver nodes. Such approaches offer a promising framework to control bipartite networks and study their undesired behavior [202].

Examples of bipartite network analysis

In previous sections, we discussed some of the topological features of bipartite graphs and we presented the main categories of biological bipartite networks. Here, we present some examples of bipartite network analysis, using both artificial data and real ones.

Detection of patterns using topological features
We used NAP application [36] in order to give numeric calculations of several of the topological features and metrics described earlier. In Figure 6 we visualize a small bipartite graph with its two projected networks and show numeric calculations about their density, the average path length, the clustering coefficient, the modularity yja betweenness centrality, the closeness centrality and the average connectivity degree. Furthermore, we show an automatically calculated ranking of the nodes related to their connectivity and betweenness centrality; nodes with higher rank appear first. In Figure 7, we show how some of these features of the two projected networks change in relation to the bipartite graph's topology. For example, in Figure 7A, it is shown that the more nested a bipartite graph is, the lower the clustering coefficient is as it does not tend to form clusters. Similarly, the higher the nestedness of the bipartite graph, the higher the betweenness centrality of its projected networks. A fully nested bipartite graph which generates two fully connected networks (cliques) is shown in Figure 7B. In addition, we observe how nestedness affects the betweenness centrality as well as the centralization degree (hubs). Both are zero since there are no hubs and no nodes bridging communities. In Figure 8 the extent to which the modularity of a bipartite graph can affect the topology of the two projections is shown, which also allows us to infer similarity conclusion.

Analysis of the Gene-Disease Network
As we already mentioned, network-based approaches for the discovery of gene-disease associations have enabled biomedical researchers to investigate the genetic complexity of a particular disease, and the relatedness among apparently discrete disease phenotypes. In order to illustrate the steps need to be followed in the analysis of such a network, we used as a test case the bipartite networks that contained the associations between the human diseases and the genes that confer susceptibility to these disease, from the study conducted by Kontou et al. [15]. The particular analysis was performed by combining data from OMIM and from two other primary resources containing information of gene-disease associations, the NIH's Genetic Association Database (GAD) and the National Human Genome Research Institute (NHGRI) Catalog of Published Genome-Wide Association Studies (GWAS). In the original publication the datasets were combined but here for purposes of illustration we used only the GAD dataset, in order to avoid confusion to the readers.

The original data can be found in Kontou et al. [203] and the user can directly upload the biadjacency matrix to a network analysis tool in order to calculate topological features of bipartite networks and visualize the bipartite structure. For the analysis, the visual representation and the projection, we used NAP [36], igraph [204] and bigraph [205], as well as BiLayout [206] and PowerClust [207]. For the detailed presentation of the tools, see the corresponding section. Figure 9A shows numeric calculations regarding the density, diameter, clustering coefficient, modularity,

betweenness and closeness centrality, connectance, generality and vulnerability of the GAD bipartite network. The mean number of genes per disease is 14.82, whereas the mean number of diseases per gene is 1.21. The statistical properties of the network are captured by the proposed metrics, that reveal a moderately dense, asymmetric network (few genes, many diseases), with modular architecture, having a moderate degree of betweeness centralization and low closeness centralization. These properties, dictate further the properties of the projected unipartite networks. The disease-disease network is denser, with smaller diameter and clustering coefficient equal to 0.44, whereas the gene-gene network is wider with smaller density, but larger tendency to form clusters (coefficient equal to 0.75). Betweeness and closeness centralities are comparable for the two projected networks. In Figure 9C-9G we also present various visualizations of the bipartite structure as well as of the projected networks.

Bipartite graphs, the BioMedical Big-Data era and some hints for the future

Today, we live in the big data era, where the exponential growth of information in the Biosphere is evident. The protein and genome landscapes change continuously as new and hypothetical proteins and genome fragments appear every day. IMG [11] today includes ~60,00 Bacterial, 1,500 Archaeal, ~300 Eukaryotic, ~8,000 Viral isolate genomes, ~1,200 genome fragments, 6,500 metagenomes and ~2,000 metatranscriptomes. Based on a very approximate estimate, this corresponds to ~70 Million proteins coming from the isolate genomes and ~4-10 billion proteins coming from the metagenomes and metatranscriptomes. In addition, UniProtKB/TrEMBL release 15-Feb-2017 [208] contains ~77,500,00 sequence entries. Moreover, Uniparc contains ~150,000,000 protein entries. PFAM [209] version 31.0, a database of a large collection of protein families, organizes proteins in families by similar domains and consists of ~17,000 entries. NCBI today hosts one billion sequences corresponding to 2.2 trillion bases and RefSeq alone hosts more than 100 million complete accessions. Moreover, PubMed hosts more than ~27 Million articles today. Also, other databases which host results from high-throughput experiments increase in size every day. Thus, comparative genomics and integrative biology are areas that already have and are expected to experience a blast over the next years and dominate other areas within the broader big-data spectrum (e.g. internet of things (IoT)).

Another important attribute of the biomedical bipartite networks, which reflect the abstract nature of the entities that they contain, is that they make extensive use of data integration techniques and rely on incorporating data from multiple sources (diseases, SNPs, gene expression, PPIs, clinical symptoms, pharmaceutical drugs etc.), contrary to the ecological and molecular networks. This highlights the need for the creation of publicly available biological databases containing high quality data. Biological databases, in general, play a central role in bioinformatics, since they offer scientists the opportunity to access a wide variety of biologically relevant data [210]. Furthermore, they are indispensable in the context of network medicine and systems biology and medicine, since the primary data of several databases need to be integrated in order to achieve the desired result [211, 212]. The biological databases continue to grow and the need for data integration techniques, as well as the potential applications in network medicine

and systems biology, also increases [213, 214]. Initiatives for standardization and construction of ontologies is also of paramount importance in this respect. Currently, available and up-to-date databases exist for a large variety of data, including SNPs [215], RNAs [216], PPIs [217, 218], biomolecular pathways [219], drugs [220-222] and diseases [223]. However, the gene-disease relationships, which form the basis of biomedical networks, are considered especially problematic, since genetic association studies are characterized by non-replicability [224, 225] and most approaches to collecting data for gene-disease analysis are based on the clearest gene-disease associations derived from the literature. In this respect, OMIM and GWAS Catalog are indispensable resources, but the recent discontinuation of GAD signifies the need for a more sophisticated resource that will contain replicated and unbiased genetic association data.

Today's high-performance computing capabilities allow for analysis of massive networks but scalability, analysis and visualization still remains a bottleneck [226]. In terms of visualization for example, layouting a bi- or an n-partite network still remains a challenge. While efficient layout algorithms such as the OpenOrd [227] and Yifan-Hu [228] can be applied on generic networks, limited efforts have been implemented to layout large scale n-partite networks, thus rendering the visualization of such networks with current methods unattractive. Therefore, the need of efficient visualization and layouting emerges.

In terms of network analysis, clustering is one of the most active research fields. While a great plethora of generic clustering algorithms exist, great efforts have been made in the BioMedical area in order to incorporate such algorithms within established network visualization tools. Cytoscape's ClusterMaker plugin [229], for example, includes attribute cluster algorithms such as AutoSOME Clustering [230], Eisen's hierarchical and k-Means clustering [231], as well as topology-based clustering algorithms such as affinity propagation [232], community clustering (GLay) [233], MCODE [234], MCL [235], SCPS (Spectral Clustering of Protein Sequences) [236] and transitivity clustering [237]. While these efforts have been proven very fruitful, often users misuse these algorithms without taking into consideration the topological characteristics of the network. As bipartite graphs come with their own properties, the implementation of scalable clustering algorithms taking advantage of their topology would be very powerful.

Overall, analysis, layout and visualization adjusted to bipartite, and further extended to n-partite graphs, is still in its infancy and constitutes a big gap in the BioMedical field. Therefore, we believe that, efficient and scalable tools covering these needs would become protagonists in the field in the future.

Software and tools for bipartite graphs

In this section, we discuss software applications and libraries that are available for the analysis and visualization of bipartite and n-partite networks. While, tools for analysis and visualization of unipartite biological networks of general use are presented and analyzed elsewhere [238-241], Table 1 summarizes their functionalities whereas Figure 10 shows how they can be used for visualizing bi- and n-partite graphs. However, in most cases, specialized software is needed either in the form of a plugin for an existing tool or as a completely different package.

Cytoscape [242] is an open source, bioinformatics-oriented software platform mainly implemented to analyze and visualize generic interaction networks. Although it does not specialize in bipartite graphs, some functionality for visualizing and processing such graphs is available through several plugins [243]. Nevertheless, it comes with a plethora of simple and more sophisticated layout algorithms. Therefore, given a bipartite graph G=(U, V, E), vertices of the disjoint sets U and V can be selected, placed separately and organized by using local simple grid, hierarchical or circular layouts. In addition, in order to easily follow the nodes of each layer, vertices of different groups can be colored accordingly.

DisGeNET [244] is a Cytoscape plugin especially designed to analyze human gene–disease association networks. DisGeNET allows users to access a gene–disease database containing integrated data from diverse public resources. DisGeNET presents the gene–disease networks (diseasome) as bipartite graphs and provides the option to view gene-gene and disease-disease networks derived from the diseasome. Advanced search options permit the generation of subnetworks and the analysis of sets of diseases associated through common genes.

BiLayout is a Java plugin, able to compute a bipartite network layout for two groups of nodes. BiLayout allows some simple actions such as selecting one of the groups, showing and hiding unconnected nodes, exporting groups of nodes and resetting the network. The mouse-over effect allows the user-friendly and customized visualization of all neighbors of a certain node.

Pajek [245] is a free, noncommercial Windows (32 bit) program package for analysis and visualization of large networks (networks containing up to one billion of vertices and an unlimited number of edges). Pajek implements several methods for the visualization of bipartite graphs and for the analysis of the unipartite projections of the bipartite graph.

NetworkX [246] is a software package for the generation, processing and analysis of several types of graphs including bipartite graphs. A node attribute named “bipartite” with values 0 or 1 enables the identification of the corresponding set of each node. The user, also, has to make sure that there are no links between nodes that belong to the same set. NetworkX, although it requires user intervention for creating bipartite networks, it provides several options for bipartite network drawing, projection and data analysis.

UCINET [247] is a commercial software package for Windows designed primarily for the analysis of social network data. It is accompanied by the NetDraw network tool that can handle and visualization of bipartite networks. The tool contains several options to calculate network metrics that, however, are optimized for the analysis of unipartite graphs. Nevertheless, UCINET also contains modules for projecting the bipartite networks.

Gephi [248]: It is one of the best open-source visualization and exploration software for all kinds of graphs and networks. It can easily render networks that consist of up to 100,000 nodes and 1,000,000 edges.

FALCON [249] is a software package devoted to the analysis of ecological networks which allows user-friendly and efficient calculations of network metrics such as nestedness scores using state-of-the-art measures and models. The FALCON code is available in three programming languages (R, MATLAB, Octave) and allows users to install further measures and null models easily.

Arena3D [250, 251] is an interactive and freely available 3D generic tool, mainly intended to visualize multi-layered graphs. It uses a layered display to separate different levels of information while emphasizing on the connections between them. Among other functionalities (i.e. great variety of clustering algorithms), Arena3D can be utilized to visualize intra- and inter-network connections, show gene expressions levels and handle time course data in a phenotypic context. Arena's concept can be easily adjusted to visualize bipartite graphs as vertices of the disjoint sets U and V of a bipartite graph $G=(U, V, E)$ can be separated onto different layers and colored accordingly. Connections across the different layers can easily be loaded and visualized simultaneously. While nodes can be placed anywhere manually, clustering across layers can place the vertices of each layer in a way that crossovers between lines can be minimized. Although Arena3D might be too advanced for the visualization of simple bipartite graphs, it is highly recommended for n-partite graphs, where n layers can be placed anywhere and in various orientations in 3D space, thereby offering very sophisticated visualizations. An example is shown in **Figure 10E**.

The ***Biclustering Analysis Toolbox*** **(BicAT)** [252] is a software platform for the analysis of gene interconnection networks, as well other types of data (e.g., proteomics data), based on biclustering techniques in a single graphical interface. Furthermore, BicAT offers a variety of facilities (e.g., filtering of biclusters) for data preparation, review, processing and post analysis. The user is able to choose the optimal/their preferred biclustering algorithm among different algorithms. The program allows the users to install further extensions or algorithms.

GeneWeaver [253] is an online software for the integration of functional genomics experiments. *GeneWeaver* contains a set of interactive tools for analysis and visualization of gene sets, gene set descriptions and gene set association scores from multiple species. It differs from conventional gene set over-representation analysis tools in that it allows users to evaluate intersections among all combinations of a collection of gene sets, including, but not limited to annotations to controlled vocabularies. Gene sets can come from many different sources, (microarray experiments, Gene Ontology annotations, Text Mining tools, list of specific genes etc).

ONEMODE [254] is a Stata module capable of producing one-mode projections of a bipartite network. This package offers the most complete collection of algorithms for projection, such as methods for unconditional (global) threshold, methods with thresholds conditioned on the U-nodes' degree, methods for controlling U-nodes' differing numbers of interacting V-nodes, the Fixed Degree Sequence Model (FDSM) and the Stochastic Degree Sequence Model (SDSM).

Circos [255] is a tool widely used in comparative genomics to visualize structural variations and direct comparisons between genomes. It uses a circular ideogram layout to facilitate the display

of relationships between pairs of positions by the use of ribbons, which encode the position, size, and orientation of related genomic elements. A potential usage of Circos in terms of bipartite network visualization is shown in **Figure 10C**.

Hiveplots [256] is a rational method for drawing and visualizing networks. Nodes are mapped to and positioned on radially distributed linear axes. While the purpose of the hive plot is to establish a new baseline for visualization of large networks, we believe that it is a very suitable tool to visualize large scale *n*-partite and especially tripartite graphs. An example of the application of Hiveplots is shown in **Figure 10D**.

Other generic visualization tools that could potentially adjust to efficiently visualize bipartite graphs are the 2D standalone applications like graphVizdb [257], Ondex [258], Proviz [259], VizANT [260], GUESS [261], UCINET [262], MAPMAN [263], PATIKA [264], Medusa [265] or Osprey [266], as well as 3D visualization tools such as BioLayout Express [267].

R packages

R is a software environment and a programming language for statistical analysis supported by the R Foundation for Statistical Computing. The R language is widely used among researchers for developing statistical software and data analysis. R is freely available under the GNU General Public License. R contains several packages that can handle bipartite networks. Some of them are oriented towards the analysis of ecological networks (Networksis, enaR, Bipartite), whereas other tools were designed for more general network analyses.

Networksis [268] is a package for R, built for the analysis of ecological networks, as well as the generation of seed graphs for Markov chain Monte Carlo simulations. The tool provides several methods and many options to visualize and analyze bipartite networks. It offers also the option to calculate a series of indices summarizing the bipartite network topology. Finally, given that the ability to simulate graphs with given properties is important for the analysis of networks, the package can be used to compare results to null models. Networksis uses sequential importance sampling that has been shown to be particularly effective in estimating the number of graphs adhering to fixed marginals and in estimating the null distribution of graph statistics.

enaR [269] is an R package for Ecosystem Network Analysis (ENA). ENA is a suite of analytical tools for studying the structure and dynamics of energy and matter fluxes through distinct ecological compartments.

BipartiteR [270] is an R package containing utilities to visualize bipartite networks and compute a set of indices that are often used to describe different aspects of food webs, for instance, pollination webs or predator-prey-webs.

Netpredictor [271] is an R package (available also as an R Shiny web application) especially designed for the prediction of missing links in bipartite networks. The package provides a set of tools for calculating missing links in both bipartite and unipartite networks. Also, Netpredictor

allows computation of several bipartite network properties, calculation of significant interactions between two sets of nodes using permutation-based testing and visualization of communities for two different sets of nodes.

biGRAPH [205] is an R package, extension to the well-known *i*graph package (which is the method of choice for handling unipartite graphs), which provides a set of methods specifically designed for the analysis of bipartite graphs, including the projection of bipartite graphs handling the problem of information loss. In addition, clustering and community detection among vertex subsets is supported by providing metric distance calculations based on flexible (weighted) neighborhoods. The latest version of the software package contains also some of the metrics for bipartite graphs proposed by Borgatti and Everett [34], including measures for density, vertex centrality, and centralization with respect to each vertex subset.

tnet [272] is an R package that among others can handle the analysis of bipartite networks. Although this tool contains several projection methods, it is optimally designed to handle bipartite weighted networks.

BiRewire [273] is an R package in Bioconductor that implements the Switching Algorithm for the randomisation of bipartite graphs retaining their node degrees (i.e. Network Rewiring). BiRewire can be also used for the randomization of general presence (1)-absence (0) matrices, where the presence distributions must be preserved. Specifically, BiRewire enables users to generate bipartite graphs from any ‘0-1’ matrix, as well as rewired versions of these graphs.

DEsubs [274] is R package especially designed to extract differentially expressed, disease-associated subpathways from a pathway network generated from RNA-seq experiments. It comes with advanced visualization and enrichment analysis with regard to various biological and pharmacological features. Its circular representation could be potentially useful for the visualization of bipartite networks.

Dataset Collections

Lastly, we present here some repositories (databases) that hold numerous biological network datasets, including bipartite ones. Even though some of the datasets mentioned earlier are highly curated and biologically important, here we restrict our attention only to collections of datasets and thus we do not list specific datasets. Some of these databases contain various datasets, even of non-biological origin (such as the SLND, ICON and KONECT), whereas there are several databases specialized to the ecological networks, highlighting the importance of such data in current network research.

The **Stanford Large Network Dataset (SLND)** accompanies the Stanford Network Analysis Platform (SNAP) library [275], which is being actively developed since 2004 and is organically growing as a result of the Leskovec group's research in analysis of large social and information networks. The datasets available on the website were mostly collected for the research performed by the team and the website is active from 2009. [276]. The **Colorado Index of Complex Networks (ICON)** [277] is a comprehensive index of network datasets from all domains of network science, including social, web, biological, ecological, transportation, and technological networks. Each network record is annotated with its graph properties, description, size, etc., and many records include links to multiple networks. The contents of ICON are curated by volunteer experts from Prof. Aaron Clauset's research group at the University of Colorado Boulder. **The Koblenz Network Collection (KONECT)** [278] is a project to collect large network datasets of all types in order to perform research in network science, collected by the Institute of Web Science and Technologies at the University of Koblenz–Landau. KONECT contains several hundred network datasets of various types, including directed, undirected, bipartite, weighted, unweighted, signed and rating networks. The networks of KONECT cover many diverse areas such as social networks, hyperlink networks, authorship networks, physical networks, interaction networks, and communication networks [279].

The ecological databases include the Web of Life, the Interaction Web Database and the Kelpforest Database. The **Web of Life** [280] provides a graphical user interface, based on Google Maps, for visualization and download of data on ecological networks regarding species interactions. It is designed and implemented in a relational database, allowing sophisticated and user-friendly searches. Data can be downloaded in several common formats and a web-service for data transmission in JavaScript Object Notation is also provided. The **Interaction Web Database** [281] contains datasets on species interactions from several communities in different parts of the world. Data currently available cover a variety of interaction types, including plant-pollinator, plant-frugivore, plant-herbivore, plant-ant mutualist and predator-prey interactions. The developers' goal is to expand the database to make it a repository of data on any kind of interactions. The **Kelpforest Database** [282] serves as a repository for the knowledge of identities, life-histories, and interactions between the species present in the nearshore kelp forest ecosystems of the eastern Pacific, focusing in particular on central and southern California. The information that contains could aid in the interpretation of species' spatial and temporal patterns, and serve as the basis on which to construct and parameterize mathematical models of these species rich communities [283].

Conclusions

Network-based approaches have been used routinely during the last decade in order to analyze the massive amount of biological/biomedical data produced from the modern high-throughput experiments. Bipartite networks constitute an important, but usually overlooked and difficult to analyze class of networks. However, given that natively bipartite structures have many applications in systems biology and medicine, there is an emerging need for specialized methods and software in order to analyze such networks. Based on a review of the literature, ecological networks, which are traditionally constructed by collecting large samples of individuals from the field, are usually analyzed as bipartite networks using the native structure. On top of that, research on ecological networks has produced many network metrics especially designed for bipartite graphs. Several studies have introduced new indices to describe network properties and consequently dozens of indices are currently available to address similar questions[35].

On the other hand, the biomedical networks are usually analyzed through projection and analysis of the projected unipartite networks. This is of no surprise, since most of the times the biomedical networks connect abstract entities, such as “diseases”, “genes” or “symptoms”, and in most cases the primary goal of the analysis is the direct interactions between members of the same group. Nevertheless, projection of a bipartite network into its unipartite counterparts results in loss of information. Another issue that needs to be investigated is whether and to what extend the different methods of projection proposed in the literature affect the overall results of such analysis. Additionally, it could be particularly useful to examine if any of the natively bipartite methods or metrics that have been developed for ecological analysis (e.g., nestedness, modularity, community detection, flow etc.) can also be applied in the case of molecular or biomedical networks, such as the diseasome.

Convergence of ecology and bioinformatics is expected in the near future. Such convergence has been achieved in the past, with the most prominent applications in phylogenetics, which is considered a vital part of bioinformatics, in microbial ecology and metagenomics [284], as well as in other areas of ecology [285]. Of note, in the past decade molecular methods (sequencing, metagenomics, barcoding etc,) were used extensively in studies of host–parasitoid webs to clarify species concepts [286]. Therefore, network science constitutes an interdisciplinary field, where ecologists and molecular biologists are brought together [287]. We have already noted that indices applied to ecological networks could have potential application in the analysis of biomedical and molecular networks as well. On the opposite direction, methods for identifying modules in ecological networks have stimulated much interest and several robust module-detecting algorithms that have been applied in other disciplines have also been applied in large pollination networks, showing that these networks were modular and that modularity co-occurred with nestedness [84]. In a similar manner, the large arsenal of biclustering methods described in the pertinent machine learning literature can be applied in the study of ecological or other biological networks.

Lastly, it is worth mentioning that in several cases, at least in the context of biomedical networks, researchers try to compile tripartite networks in order to model the complex interactions associated with diseases [107, 116]. This is of no surprise since most diseases are multifactorial, and affected by various genetic, environmental and lifestyle factors. Thus, due to data accumulation, additional knowledge is expected to be integrated into gene-disease networks.

Taking into account the above, future studies, at least those on the biomedical networks, should focus on the development of analytical methods and software tools capable of handling tripartite and multipartite graphs that would enable the simultaneous analysis of information from multiple sources. For instance, instead of the bipartite gene-disease network, it might be more useful to perform network data analysis without projections and analyze, for instance, a multipartite graph that illustrates exposure-gene-symptoms-disease relationships. A potential way of representing such systems would be to extend the network into multiple layers (in a multipartite graph), or use a generalization of graphs known as hypergraphs. In a simple graph, a link connects only a pair of nodes, whereas the edges of the hypergraph (hyperedges) can connect groups of more than two nodes. Towards this direction, analytical methods have been developed in order to extend the application of clustering coefficient and subgraph centrality to complex hyper-networks [34].

Figure legends

Figure 1. Construction of unipartite networks from a bipartite network. A) The bipartite network, B) The biadjacency matrix of the bipartite network, C) The first unipartite network with its adjacency matrix and D) The second unipartite network with its adjacency matrix. The adjacency matrices are symmetrical across the diagonal line.

Figure 2. Network nestedness. Example of A) a bipartite network, B) the biadjacency matrix of the bipartite network and C) The projected unipartite networks.

Figure 3. Network modularity. Example of A) a bipartite network, B) the biadjacency matrix of the bipartite network and C) The corresponding unipartite networks.

Figure 4. Mixed network (nested+modular). Example of A) a bipartite network, B) the biadjacency matrix of the bipartite network and C) the projected unipartite networks.

Figure 5. Overview and examples of various types of networks: A) Ecological networks. An example of a predator-prey (left) and host-parasite example (right) network. B) Biomedical networks. An example of a disease-gene (left) and a drug-target (right) network. C) Biomolecular networks. An example of a gene-transcription factor binding site (left) and a gene-pathway (right) network. D) Epidemiological networks. An example of a patient-location network.

Figure 6. Numerical Examples. A) a small bipartite network, its adjacency matrix, several calculated topological features for the whole graph, node ranking according to degree and betweenness centrality. B) And C) information relevant to projected unipartite networks.

Figure 7. Two examples of the way some topological features of the projected unipartite networks are affected by the bipartite graph’s nestedness. A) Nested bipartite graph. B) Fully nested bipartite graph. The higher the nestedness of the bipartite graph, the more connected the projected networks. Maximum nestedness leads to fully connected unipartite networks (cliques).

Figure 8. Example of the extent to which bipartite graph’s modularity affect the unipartite projected networks.

Figure 9. A test case of bipartite Gene-Disease network from Genetic Association Database (GAD). A) Topological Features of the whole bipartite network B) Data example of bipartite network (gene-disease). C) Circular Visualization of the bipartite network (Genes-Red, Diseases-Blue) using PowerClust. D) Random Visualization of the bipartite network showing the directed connections between the two disjoint sets of nodes using PoweCclust. E) Random Visualization of the bipartite network showing the indirect connections between the two disjoint sets of nodes

using PowerClust. F) Topological Features of the projected Disease-Disease network and an example of the monopartite network and different types of visualization. G) Topological Features of the projected Gene-Gene network and example of the monopartite network and different types of visualization.

Figure 10. Various types of visualizations of *n*-partite networks. A) Visualization using a generic network tool such as Cytoscape. Nodes from each group are colored accordingly. B) Vertical bipartite visualization. C) Circular visualization using a Circos-like view often used in genomics. D) A hive plot view visualizing a tripartite graph. E) Visualization of a multilayered network using Arena3D. F) Visualization of a bipartite network over a word-map.

Tables

Table 1. A summary of the tools dedicated to bipartite graph analysis and their properties.

Tool	Software	Library	Usage	URL
Cytoscape	**X**		**Generic network analysis tool**	**http://www.cytoscape.org/**
DisGeNET	**X**		**Cytoscape's Pluging to analyse disease-gene interactions**	**http://www.disgenet.org/web/DisGeNET**
BiLayout	**X**		**Bipartite layout**	**http://bilayout.bioinf.mpi-inf.mpg.de**
Pajek	**X**		**Generic analysis and visualization tool**	**http://vlado.fmf.uni-lj.si/pub/networks/pajek/**
NetworkX	**X**		**Analysis of several types of graphs including bipartite graphs**	**https://networkx.github.io/**
UCINET	**X**		**Social networks. NetDraw is specialized for bipartite graphs**	**https://sites.google.com/site/ucinetsoftware/home**
Gephi	**X**		**Generic network analysis tool**	**https://gephi.org/**
FALCON	**X**		**Analysis of ecological networks**	**https://github.com/sjbeckett/FALCON**
Arena3D	**X**		**Visualization of multi-layered graphs**	**http://arena3d.org/**
**BicAT**	**X**		**Analysis of networks based on biclustering techniques**	**http://www.tik.ee.ethz.ch/sop/bicat/**
GeneWeaver	**X**		**Integration of functional genomics experiments**	**https://geneweaver.org/**

ONEMODE	**X**		**Stata module for producing one-mode projections of a bipartite network**	**http://fmwww.bc.edu/repec/bocode/o/onemode.ado**
Circos	**X**		**Data visualization using a circular layout**	**http://circos.ca/**
Hiveplots	**X**		**Data visualization using radially distributed linear axes**	**http://www.hiveplot.com/**
Networksis		**X**	**Tool to simulate bipartite networks**	**https://cran.r-project.org/web/packages/networksis/index.html**
enaR		**X**	**Provides algorithms for the analysis of ecological networks**	**https://cran.r-project.org/web/packages/enaR/**
Netpredictor		***X***	**Prediction of missing links in any given bipartite network**	**https://github.com/abhik1368/Shiny_NetPredictor**
biGRAPH		***X***	**Extension of the igraph library fo bipartite graphs**	**https://cran.r-project.org/src/contrib/Archive/biGraph/**
BiRewire		***X***	**Bipartite network rewiring through N consecutive switching steps**	**https://bioconductor.org/packages/release/bioc/html/BiRewire.html**
**DEsubs**		**X**	**Visualization of disease-perturbed subpathways**	**http://bioconductor.org/packages/release/bioc/html/DEsubs.html**

Declarations

Ethics approval and consent to participate

Not applicable

Consent for publication

Not applicable

Availability of data and materials

The original data for the Analysis of the Gene-Disease Network can be found in the publications and the supplements of Kontou et al. [15, 203]. The data for generating the example networks of Figures 1-4 can be found in the Supplementary Material.

Competing interests

The authors declare that they have no competing interests.

Funding

This work was supported by the US Department of Energy Joint Genome Institute, a DOE Office of Science User Facility, under contract number DE-AC02-05CH11231 and used resources of the National Energy Research Scientific Computing Center, supported by the Office of Science of the US Department of Energy.

Authors' contributions

PGB and GP conceived the project and organized the work. All authors wrote parts of the manuscript and all authors have read and approved the final manuscript.

Acknowledgements

The authors would like to thank the associate editor and the reviewers whose comments and constructive criticism helped in improving the quality of the manuscript. All images of Figure 5A have been taken from Pixabay repository and are subject to CC0 creative commons license. According to CC0, users can freely copy, modify and redistribute the images, even for commercial purposes.

References

1. Fields S, Song O: **A novel genetic system to detect protein-protein interactions**. *Nature* 1989, **340**(6230):245-246.
2. Zhu H, Bilgin M, Bangham R, Hall D, Casamayor A, Bertone P, Lan N, Jansen R, Bidlingmaier S, Houfek T *et al*: **Global analysis of protein activities using proteome chips**. *Science* 2001, **293**(5537):2101-2105.
3. Ito T, Chiba T, Ozawa R, Yoshida M, Hattori M, Sakaki Y: **A comprehensive two-hybrid analysis to explore the yeast protein interactome**. *Proceedings of the National Academy of Sciences of the United States of America* 2001, **98**(8):4569-4574.
4. Uetz P, Giot L, Cagney G, Mansfield TA, Judson RS, Knight JR, Lockshon D, Narayan V, Srinivasan M, Pochart P *et al*: **A comprehensive analysis of protein-protein interactions in Saccharomyces cerevisiae**. *Nature* 2000, **403**(6770):623-627.
5. McCraith S, Holtzman T, Moss B, Fields S: **Genome-wide analysis of vaccinia virus protein-protein interactions**. *Proceedings of the National Academy of Sciences of the United States of America* 2000, **97**(9):4879-4884.
6. Davy A, Bello P, Thierry-Mieg N, Vaglio P, Hitti J, Doucette-Stamm L, Thierry-Mieg D, Reboul J, Boulton S, Walhout AJ *et al*: **A protein-protein interaction map of the Caenorhabditis elegans 26S proteasome**. *EMBO reports* 2001, **2**(9):821-828.
7. Rain JC, Selig L, De Reuse H, Battaglia V, Reverdy C, Simon S, Lenzen G, Petel F, Wojcik J, Schachter V *et al*: **The protein-protein interaction map of Helicobacter pylori**. *Nature* 2001, **409**(6817):211-215.
8. Wang Z, Gerstein M, Snyder M: **RNA-Seq: a revolutionary tool for transcriptomics**. *Nature reviews Genetics* 2009, **10**(1):57-63.
9. Goodwin S, McPherson JD, McCombie WR: **Coming of age: ten years of next-generation sequencing technologies**. *Nature reviews Genetics* 2016, **17**(6):333-351.
10. Pavlopoulos GA, Oulas A, Iacucci E, Sifrim A, Moreau Y, Schneider R, Aerts J, Iliopoulos I: **Unraveling genomic variation from next generation sequencing data**. *BioData mining* 2013, **6**(1):13.

11. Chen IA, Markowitz VM, Chu K, Palaniappan K, Szeto E, Pillay M, Ratner A, Huang J, Andersen E, Huntemann M *et al*: **IMG/M: integrated genome and metagenome comparative data analysis system**. *Nucleic acids research* 2017, **45**(D1):D507-D516.
12. Pavlopoulos GA, Secrier M, Moschopoulos CN, Soldatos TG, Kossida S, Aerts J, Schneider R, Bagos PG: **Using graph theory to analyze biological networks**. *BioData mining* 2011, **4**:10.
13. Yu D, Kim M, Xiao G, Hwang TH: **Review of biological network data and its applications**. *Genomics & informatics* 2013, **11**(4):200-210.
14. Burgos E, Ceva H, Hernandez L, Perazzo RP, Devoto M, Medan D: **Two classes of bipartite networks: nested biological and social systems**. *Physical review E, Statistical, nonlinear, and soft matter physics* 2008, **78**(4 Pt 2):046113.
15. Kontou PI, Pavlopoulou A, Dimou NL, Pavlopoulos GA, Bagos PG: **Network analysis of genes and their association with diseases**. *Gene* 2016, **590**(1):68-78.
16. Davis A, Gardner BB, Gardner MR: **Deep South: A social anthropological study of caste and class**: Univ of South Carolina Press; 2009.
17. Watts DJ, Strogatz SH: **Collective dynamics of 'small-world'networks**. *Nature* 1998, **393**(6684):440-442.
18. Newman ME: **Scientific collaboration networks. I. Network construction and fundamental results**. *Physical review E* 2001, **64**(1):016131.
19. Van Noorden R: **Online collaboration: Scientists and the social network**. *Nature* 2014, **512**(7513):126-129.
20. Newman ME: **The structure of scientific collaboration networks**. *Proceedings of the National Academy of Sciences* 2001, **98**(2):404-409.
21. Conyon MJ, Muldoon MR: **The small world of corporate boards**. *Journal of Business Finance & Accounting* 2006, **33**(9-10):1321-1343.
22. Ramasco JJ, Dorogovtsev SN, Pastor-Satorras R: **Self-organization of collaboration networks**. *Physical review E* 2004, **70**(3):036106.
23. Guillaume J-L, Latapy M, Le Blond S: **Statistical Analysis of a P2P Query Graph Based on Degrees and Their Time-Evolution**. In: *IWDC: 2004*. Springer: 126-137.
24. Taylor PJ: **The new geography of global civil society: NGOs in the world city network**. *Globalizations* 2004, **1**(2):265-277.
25. Doreian P, Batagelj V, Ferligoj A: **Generalized blockmodeling of two-mode network data**. *Social networks* 2004, **26**(1):29-53.
26. Fowler JH: **Legislative cosponsorship networks in the US House and Senate**. *Social Networks* 2006, **28**(4):454-465.
27. Deng H, Lyu MR, King I: **A generalized co-hits algorithm and its application to bipartite graphs**. In: *Proceedings of the 15th ACM SIGKDD international conference on Knowledge discovery and data mining: 2009*. ACM: 239-248.
28. YANAI K: **VisualTextualRank: an extension of visualrank to large-scale video shot extraction exploiting tag co-occurrence**. *IEICE TRANSACTIONS on Information and Systems* 2015, **98**(1):166-172.
29. Cao L, Guo J, Cheng X: **Bipartite graph based entity ranking for related entity finding**. In: *Web Intelligence and Intelligent Agent Technology (WI-IAT), 2011 IEEE/WIC/ACM International Conference on: 2011*. IEEE: 130-137.
30. Li X, Chen H: **Recommendation as link prediction in bipartite graphs: A graph kernel-based machine learning approach**. *Decision Support Systems* 2013, **54**(2):880-890.
31. Xu K, Wang F, Gu L: **Behavior analysis of internet traffic via bipartite graphs and one-mode projections**. *IEEE/ACM Transactions on Networking (TON)* 2014, **22**(3):931-942.
32. Cai J, Liu WX: **A new Method of detecting network traffic anomalies**. In: *Applied Mechanics and Materials: 2013*. Trans Tech Publ: 912-916.

33. Latapy M, Magnien C, Del Vecchio N: **Basic notions for the analysis of large two-mode networks**. *Social networks* 2008, **30**(1):31-48.
34. Borgatti SP, Everett MG: **Network analysis of 2-mode data**. *Social networks* 1997, **19**(3):243-269.
35. Dormann CF, Fründ J, Blüthgen N, Gruber B: **Indices, graphs and null models: analyzing bipartite ecological networks**. 2009.
36. Theodosiou T, Efstathiou G, Papanikolaou N, Kyrpides NC, Bagos PG, Iliopoulos I, Pavlopoulos GA: **NAP: The Network Analysis Profiler, a web tool for easier topological analysis and comparison of medium-scale biological networks**. *BMC Res Notes* 2017, **10**(1):278.
37. Doncheva NT, Assenov Y, Domingues FS, Albrecht M: **Topological analysis and interactive visualization of biological networks and protein structures**. *Nat Protoc* 2012, **7**(4):670-685.
38. Leskovec J, Sosic R: **SNAP: A General Purpose Network Analysis and Graph Mining Library**. *ACM Trans Intell Syst Technol* 2016, **8**(1).
39. Daugulis P: **A note on a generalization of eigenvector centrality for bipartite graphs and applications**. *networks* 2012, **59**(2):261-264.
40. Wasserman S, Faust K: **Social network analysis: Methods and applications**, vol. 8: Cambridge university press; 1994.
41. Lind PG, González MC, Herrmann HJ: **Cycles and clustering in bipartite networks**. *Physical review E* 2005, **72**(5):056127.
42. Opsahl T: **Triadic closure in two-mode networks: Redefining the global and local clustering coefficients**. *Social Networks* 2013, **35**(2):159-167.
43. Robins G, Alexander M: **Small worlds among interlocking directors: Network structure and distance in bipartite graphs**. *Computational & Mathematical Organization Theory* 2004, **10**(1):69-94.
44. Zhang P, Wang J, Li X, Li M, Di Z, Fan Y: **Clustering coefficient and community structure of bipartite networks**. *Physica A: Statistical Mechanics and its Applications* 2008, **387**(27):6869-6875.
45. Staniczenko PP, Kopp JC, Allesina S: **The ghost of nestedness in ecological networks**. *Nature communications* 2013, **4**:1391.
46. Ulrich W, Almeida-Neto M, Gotelli NJ: **A consumer's guide to nestedness analysis**. *Oikos* 2009, **118**(1):3-17.
47. Atmar W, Patterson BD: **The measure of order and disorder in the distribution of species in fragmented habitat**. *Oecologia* 1993, **96**(3):373-382.
48. Araujo AI, Corso G, Almeida AM, Lewinsohn TM: **An analytic approach to the measurement of nestedness in bipartite networks**. *Physica A: Statistical Mechanics and its Applications* 2010, **389**(7):1405-1411.
49. Guimarães PR, Guimaraes P: **Improving the analyses of nestedness for large sets of matrices**. *Environmental Modelling & Software* 2006, **21**(10):1512-1513.
50. Wright DH, Reeves JH: **On the meaning and measurement of nestedness of species assemblages**. *Oecologia* 1992, **92**(3):416-428.
51. Bustos S, Gomez C, Hausmann R, Hidalgo CA: **The dynamics of nestedness predicts the evolution of industrial ecosystems**. *PloS one* 2012, **7**(11):e49393.
52. Almeida-Neto M, Ulrich W: **A straightforward computational approach for measuring nestedness using quantitative matrices**. *Environmental Modelling & Software* 2011, **26**(2):173-178.
53. Galeano J, Pastor JM, Iriondo JM: **Weighted-interaction nestedness estimator (WINE): a new estimator to calculate over frequency matrices**. *Environmental Modelling & Software* 2009, **24**(11):1342-1346.

54. Newman ME, Girvan M: **Finding and evaluating community structure in networks**. *Physical review E* 2004, **69**(2):026113.
55. Fortuna MA, Stouffer DB, Olesen JM, Jordano P, Mouillot D, Krasnov BR, Poulin R, Bascompte J: **Nestedness versus modularity in ecological networks: two sides of the same coin?** *Journal of Animal Ecology* 2010, **79**(4):811-817.
56. Allali O, Tabourier L, Magnien C, Latapy M: **Internal links and pairs as a new tool for the analysis of bipartite complex networks**. *Social Network Analysis and Mining* 2013, **3**(1):85-91.
57. Holme P, Liljeros F, Edling CR, Kim BJ: **Network bipartivity**. *Physical Review E* 2003, **68**(5):056107.
58. Estrada E, Rodríguez-Velázquez JA: **Spectral measures of bipartivity in complex networks**. *Physical Review E* 2005, **72**(4):046105.
59. Pisanski T, Randić M: **Use of the Szeged index and the revised Szeged index for measuring network bipartivity**. *Discrete Applied Mathematics* 2010, **158**(17):1936-1944.
60. Blüthgen N, Fründ J, Vázquez DP, Menzel F: **What do interaction network metrics tell us about specialization and biological traits**. *Ecology* 2008, **89**(12):3387-3399.
61. Mukherjee A, Choudhury M, Ganguly N: **Understanding how both the partitions of a bipartite network affect its one-mode projection**. *Physica A: Statistical Mechanics and its Applications* 2011, **390**(20):3602-3607.
62. Guillaume J-L, Latapy M: **Bipartite graphs as models of complex networks**. *Physica A: Statistical Mechanics and its Applications* 2006, **371**(2):795-813.
63. Nacher J, Akutsu T: **On the degree distribution of projected networks mapped from bipartite networks**. *Physica A: Statistical Mechanics and its Applications* 2011, **390**(23):4636-4651.
64. Li M, Fan Y, Chen J, Gao L, Di Z, Wu J: **Weighted networks of scientific communication: the measurement and topological role of weight**. *Physica A: Statistical Mechanics and its Applications* 2005, **350**(2):643-656.
65. Neal Z: **Identifying statistically significant edges in one-mode projections**. *Social Network Analysis and Mining* 2013, **3**(4):915-924.
66. Neal Z: **The backbone of bipartite projections: Inferring relationships from co-authorship, co-sponsorship, co-attendance and other co-behaviors**. *Social Networks* 2014, **39**:84-97.
67. Newman ME: **Scientific collaboration networks. II. Shortest paths, weighted networks, and centrality**. *Physical review E* 2001, **64**(1):016132.
68. Zhou T, Ren J, Medo M, Zhang Y-C: **Bipartite network projection and personal recommendation**. *Physical Review E* 2007, **76**(4):046115.
69. Zweig KA, Kaufmann M: **A systematic approach to the one-mode projection of bipartite graphs**. *Social Network Analysis and Mining* 2011, **1**(3):187-218.
70. Puebla JG: **Spatial structures of network flows: A graph theoretical approach**. *Transportation Research Part B: Methodological* 1987, **21**(6):489-502.
71. Serrano MÁ, Boguná M, Vespignani A: **Extracting the multiscale backbone of complex weighted networks**. *Proceedings of the national academy of sciences* 2009, **106**(16):6483-6488.
72. Bonacich P: **Technique for analyzing overlapping memberships**. *Sociological methodology* 1972, **4**:176-185.
73. Borgatti SP, Halgin DS: **Analyzing affiliation networks**. *The SAGE handbook of social network analysis* 2011:417-433.
74. Scott J: **Social network analysis: developments, advances, and prospects**. *Social network analysis and mining* 2011, **1**(1):21-26.

75. Tumminello M, Miccichè S, Lillo F, Piilo J, Mantegna RN: **Statistically validated networks in bipartite complex systems**. *PloS one* 2011, **6**(3):e17994.
76. Klecka J: **The role of a water bug, Sigara striata, in freshwater food webs**. *PeerJ* 2014, **2**:e389.
77. Dunne JA, Williams RJ, Martinez ND: **Food-web structure and network theory: the role of connectance and size**. *Proceedings of the National Academy of Sciences* 2002, **99**(20):12917-12922.
78. Kondoh M, Kato S, Sakato Y: **Food webs are built up with nested subwebs**. *Ecology* 2010, **91**(11):3123-3130.
79. Hassell M, Waage J: **Host-parasitoid population interactions**. *Annual review of entomology* 1984, **29**(1):89-114.
80. Henri DC, Van Veen F: **Body size, life history and the structure of host-parasitoid networks**. *Advances in Ecological Research* 2011, **45**:135-180.
81. Bascompte J, Jordano P: **Plant-animal mutualistic networks: the architecture of biodiversity**. *Annual Review of Ecology, Evolution, and Systematics* 2007:567-593.
82. Dáttilo W, Guimarães PR, Izzo TJ: **Spatial structure of ant–plant mutualistic networks**. *Oikos* 2013, **122**(11):1643-1648.
83. Bascompte J, Jordano P, Melián CJ, Olesen JM: **The nested assembly of plant–animal mutualistic networks**. *Proceedings of the National Academy of Sciences* 2003, **100**(16):9383-9387.
84. Olesen JM, Bascompte J, Dupont YL, Jordano P: **The modularity of pollination networks**. *Proceedings of the National Academy of Sciences* 2007, **104**(50):19891-19896.
85. James A, Pitchford JW, Plank MJ: **Disentangling nestedness from models of ecological complexity**. *Nature* 2012, **487**(7406):227-230.
86. Montoya JM, Solé RV: **Small world patterns in food webs**. *Journal of theoretical biology* 2002, **214**(3):405-412.
87. Jordano P, Bascompte J, Olesen JM: **Invariant properties in coevolutionary networks of plant–animal interactions**. *Ecology letters* 2003, **6**(1):69-81.
88. Vázquez DP: **Degree distribution in plant–animal mutualistic networks: forbidden links or random interactions?** *Oikos* 2005, **108**(2):421-426.
89. Williams RJ: **Biology, methodology or chance? The degree distributions of bipartite ecological networks**. *PLoS One* 2011, **6**(3):e17645.
90. Corel E, Lopez P, Méheust R, Bapteste E: **Network-Thinking: Graphs to Analyze Microbial Complexity and Evolution**. *Trends in Microbiology* 2016, **24**(3):224-237.
91. Barabasi AL, Gulbahce N, Loscalzo J: **Network medicine: a network-based approach to human disease**. *Nature reviews Genetics* 2011, **12**(1):56-68.
92. Goh K-I, Choi I-G: **Exploring the human diseasome: the human disease network**. *Briefings in functional genomics* 2012:els032.
93. Goh KI, Cusick ME, Valle D, Childs B, Vidal M, Barabasi AL: **The human disease network**. *Proc Natl Acad Sci U S A* 2007, **104**(21):8685-8690.
94. Amberger JS, Bocchini CA, Schiettecatte F, Scott AF, Hamosh A: **OMIM.org: Online Mendelian Inheritance in Man (OMIM(R)), an online catalog of human genes and genetic disorders**. *Nucleic acids research* 2015, **43**(Database issue):D789-798.
95. Barrenas F, Chavali S, Holme P, Mobini R, Benson M: **Network properties of complex human disease genes identified through genome-wide association studies**. *PLoS One* 2009, **4**(11):e8090.
96. Liu CC, Tseng YT, Li W, Wu CY, Mayzus I, Rzhetsky A, Sun F, Waterman M, Chen JJ, Chaudhary PM *et al*: **DiseaseConnect: a comprehensive web server for mechanism-based disease-disease connections**. *Nucleic acids research* 2014, **42**(Web Server issue):W137-146.

97. Darabos C, Harmon SH, Moore JH: **Using the bipartite human phenotype network to reveal pleiotropy and epistasis beyond the gene**. *Pacific Symposium on Biocomputing Pacific Symposium on Biocomputing* 2014:188-199.
98. Becker KG, Barnes KC, Bright TJ, Wang SA: **The genetic association database**. *Nature genetics* 2004, **36**(5):431-432.
99. Welter D, MacArthur J, Morales J, Burdett T, Hall P, Junkins H, Klemm A, Flicek P, Manolio T, Hindorff L *et al*: **The NHGRI GWAS Catalog, a curated resource of SNP-trait associations**. *Nucleic acids research* 2014, **42**(Database issue):D1001-1006.
100. Gray KA, Yates B, Seal RL, Wright MW, Bruford EA: **Genenames.org: the HGNC resources in 2015**. *Nucleic acids research* 2015, **43**(Database issue):D1079-1085.
101. Pawson T, Linding R: **Network medicine**. *FEBS letters* 2008, **582**(8):1266-1270.
102. Hartwell LH, Hopfield JJ, Leibler S, Murray AW: **From molecular to modular cell biology**. *Nature* 1999, **402**(6761 Suppl):C47-52.
103. Oti M, Brunner HG: **The modular nature of genetic diseases**. *Clinical genetics* 2007, **71**(1):1-11.
104. Baranzini SE: **The genetics of autoimmune diseases: a networked perspective**. *Current opinion in immunology* 2009, **21**(6):596-605.
105. Ahmed SS, Ahameethunisa AR, Santosh W, Chakravarthy S, Kumar S: **Systems biological approach on neurological disorders: a novel molecular connectivity to aging and psychiatric diseases**. *BMC systems biology* 2011, **5**:6.
106. Chan SY, White K, Loscalzo J: **Deciphering the molecular basis of human cardiovascular disease through network biology**. *Current opinion in cardiology* 2012, **27**(3):202-209.
107. Hayasaka S, Hugenschmidt CE, Laurienti PJ: **A Network of Genes, Genetic Disorders, and Brain Areas**. *PLOS ONE* 2011, **6**(6):e20907.
108. Kitsak M, Sharma A, Menche J, Guney E, Ghiassian SD, Loscalzo J, Barabási A-L: **Tissue Specificity of Human Disease Module**. *Scientific Reports* 2016, **6**:35241.
109. Zhou X, Menche J, Barabási A-L, Sharma A: **Human symptoms–disease network**. *Nature communications* 2014, **5**:4212.
110. Andrieu N, Goldstein AM: **Epidemiologic and genetic approaches in the study of gene-environment interaction: an overview of available methods**. *Epidemiol Rev* 1998, **20**(2):137-147.
111. Darabos C, Grussing ED, Cricco ME, Clark KA, Moore JH: **A bipartite network approach to inferring interactions between environmental exposures and human diseases**. *Pacific Symposium on Biocomputing Pacific Symposium on Biocomputing* 2015:171-182.
112. Yildirim MA, Goh KI, Cusick ME, Barabasi AL, Vidal M: **Drug-target network**. *Nature biotechnology* 2007, **25**(10):1119-1126.
113. Cheng F, Liu C, Jiang J, Lu W, Li W, Liu G, Zhou W, Huang J, Tang Y: **Prediction of Drug-Target Interactions and Drug Repositioning via Network-Based Inference**. *PLOS Computational Biology* 2012, **8**(5):e1002503.
114. Zhang Y, Tao C, He Y, Kanjamala P, Liu H: **Network-based analysis of vaccine-related associations reveals consistent knowledge with the vaccine ontology**. *Journal of Biomedical Semantics* 2013, **4**(1):33.
115. Campillos M, Kuhn M, Gavin A-C, Jensen LJ, Bork P: **Drug Target Identification Using Side-Effect Similarity**. *Science* 2008, **321**(5886):263.
116. Chavan V, Penev L: **The data paper: a mechanism to incentivize data publishing in biodiversity science**. *BMC Bioinformatics* 2011, **12**(15):S2.
117. Cai B, Wang H, Zheng H, Wang H: **Integrating domain similarity to improve protein complexes identification in TAP-MS data**. *Proteome science* 2013, **11**(Suppl 1):S2.
118. Wu M, Li XL, Kwoh CK, Ng SK, Wong L: **Discovery of protein complexes with core-attachment structures from Tandem Affinity Purification (TAP) data**. *Journal of*

computational biology : a journal of computational molecular cell biology 2012, **19**(9):1027-1042.

119. Li Y, Thai MT, Liu Z, Wu W: **Protein-protein interaction and group testing in bipartite graphs**. *International journal of bioinformatics research and applications* 2005, **1**(4):414-419.
120. Ma B: **Challenges in Computational Analysis of Mass Spectrometry Data for Proteomics**. *Journal of Computer Science and Technology* 2010, **25**(1):107-123.
121. Junqueira M, Spirin V, Santana Balbuena T, Waridel P, Surendranath V, Kryukov G, Adzhubei I, Thomas H, Sunyaev S, Shevchenko A: **Separating the wheat from the chaff: unbiased filtering of background tandem mass spectra improves protein identification**. *Journal of proteome research* 2008, **7**(8):3382-3395.
122. Nesvizhskii AI: **Protein Identification by Tandem Mass Spectrometry and Sequence Database Searching**. In: *Mass Spectrometry Data Analysis in Proteomics.* Edited by Matthiesen R. Totowa, NJ: Humana Press; 2007: 87-119.
123. Rao VS, Srinivas K, Sujini GN, Kumar GNS: **Protein-Protein Interaction Detection: Methods and Analysis**. *International Journal of Proteomics* 2014, **2014**:12.
124. Lee J, Lee J: **Hidden Information Revealed by Optimal Community Structure from a Protein-Complex Bipartite Network Improves Protein Function Prediction**. *PLOS ONE* 2013, **8**(4):e60372.
125. Royer L, Reimann M, Andreopoulos B, Schroeder M: **Unraveling Protein Networks with Power Graph Analysis**. *PLOS Computational Biology* 2008, **4**(7):e1000108.
126. Brynildsen MP, Tran LM, Liao JC: **Versatility and connectivity efficiency of bipartite transcription networks**. *Biophysical journal* 2006, **91**(8):2749-2759.
127. Liao JC, Boscolo R, Yang Y-L, Tran LM, Sabatti C, Roychowdhury VP: **Network component analysis: Reconstruction of regulatory signals in biological systems**. *Proceedings of the National Academy of Sciences* 2003, **100**(26):15522-15527.
128. Wang X, Alshawaqfeh M, Dang X, Wajid B, Noor A, Qaraqe M, Serpedin E: **An Overview of NCA-Based Algorithms for Transcriptional Regulatory Network Inference**. *Microarrays* 2015, **4**(4).
129. Ye C, Galbraith SJ, Liao JC, Eskin E: **Using Network Component Analysis to Dissect Regulatory Networks Mediated by Transcription Factors in Yeast**. *PLOS Computational Biology* 2009, **5**(3):e1000311.
130. Platig J, Castaldi PJ, DeMeo D, Quackenbush J: **Bipartite Community Structure of eQTLs**. *PLOS Computational Biology* 2016, **12**(9):e1005033.
131. Guzmán-Vargas L, Santillán M: **Comparative analysis of the transcription-factor gene regulatory networks of E. coli and S. cerevisiae**. *BMC systems biology* 2008, **2**(1):13.
132. Horvát E-Á, Zhang JD, Uhlmann S, Sahin Ö, Zweig KA: **A Network-Based Method to Assess the Statistical Significance of Mild Co-Regulation Effects**. *PLOS ONE* 2013, **8**(9):e73413.
133. Chen L, Wang H, Zhang L, Li W, Wang Q, Shang Y, He Y, He W, Li X, Tai J *et al*: **Uncovering packaging features of co-regulated modules based on human protein interaction and transcriptional regulatory networks**. *BMC Bioinformatics* 2010, **11**(1):392.
134. Ge M, Li A, Wang M: **A Bipartite Network-based Method for Prediction of Long Non-coding RNA-protein Interactions**. *Genomics, proteomics & bioinformatics* 2016, **14**(1):62-71.
135. Bryant WA, Sternberg MJ, Pinney JW: **AMBIENT: Active Modules for Bipartite Networks - using high-throughput transcriptomic data to dissect metabolic response**. *BMC systems biology* 2013, **7**(1):26.

136. He L, Wang Y, Yang Y, Huang L, Wen Z: **Identifying the Gene Signatures from Gene-Pathway Bipartite Network Guarantees the Robust Model Performance on Predicting the Cancer Prognosis**. *BioMed research international* 2014, **2014**:10.
137. Ahnert SE: **Generalised power graph compression reveals dominant relationship patterns in complex networks**. *Scientific Reports* 2014, **4**:4385.
138. Beyer A, Workman C, Hollunder J, Radke D, Möller U, Wilhelm T, Ideker T: **Integrated Assessment and Prediction of Transcription Factor Binding**. *PLOS Computational Biology* 2006, **2**(6):e70.
139. Luke DA, Harris JK: **Network Analysis in Public Health: History, Methods, and Applications**. *Annual Review of Public Health* 2007, **28**(1):69-93.
140. Ergün G: **Human sexual contact network as a bipartite graph**. *Physica A: Statistical Mechanics and its Applications* 2002, **308**(1–4):483-488.
141. Palchykov V, Kaski K, Kertész J, Barabási A-L, Dunbar RIM: **Sex differences in intimate relationships**. *Scientific Reports* 2012, **2**:370.
142. Robinson K, Cohen T, Colijn C: **The dynamics of sexual contact networks: effects on disease spread and control**. *Theoretical population biology* 2012, **81**(2):89-96.
143. Rocha LEC, Liljeros F, Holme P: **Simulated Epidemics in an Empirical Spatiotemporal Network of 50,185 Sexual Contacts**. *PLOS Computational Biology* 2011, **7**(3):e1001109.
144. Niekamp A-M, Mercken LA, Hoebe CJ, Dukers-Muijrers NH: **A sexual affiliation network of swingers, heterosexuals practicing risk behaviours that potentiate the spread of sexually transmitted infections: A two-mode approach**. *Social Networks* 2013, **35**(2):223-236.
145. Hernández DG, Risau-Gusman S: **Epidemic thresholds for bipartite networks**. *Physical Review E* 2013, **88**(5):052801.
146. Bisanzio D, Bertolotti L, Tomassone L, Amore G, Ragagli C, Mannelli A, Giacobini M, Provero P: **Modeling the Spread of Vector-Borne Diseases on Bipartite Networks**. *PLOS ONE* 2010, **5**(11):e13796.
147. Malik HAM, Mahesar AW, Abid F, Waqas A, Wahiddin MR: **Two-mode network modeling and analysis of dengue epidemic behavior in Gombak, Malaysia**. *Applied Mathematical Modelling* 2017, **43**:207-220.
148. Hu JX, Thomas CE, Brunak S: **Network biology concepts in complex disease comorbidities**. *Nature reviews Genetics* 2016, **17**(10):615-629.
149. Hidalgo CA, Blumm N, Barabási A-L, Christakis NA: **A Dynamic Network Approach for the Study of Human Phenotypes**. *PLOS Computational Biology* 2009, **5**(4):e1000353.
150. Jensen PB, Jensen LJ, Brunak S: **Mining electronic health records: towards better research applications and clinical care**. *Nature reviews Genetics* 2012, **13**(6):395-405.
151. Hsing AW, Ioannidis JP: **Nationwide Population Science: Lessons From the Taiwan National Health Insurance Research Database**. *JAMA internal medicine* 2015, **175**(9):1527-1529.
152. **HuDiNe Dataset. http://hudine.neu.edu. Accessed 15 Jan 2018**.
153. Bhavnani SK, Dang B, Visweswaran S, Divekar R, Tan A, Karmarkar A, Ottenbacher K: **How Comorbidities Co-Occur in Readmitted Hip Fracture Patients: From Bipartite Networks to Insights for Post-Discharge Planning**. *AMIA Joint Summits on Translational Science proceedings AMIA Joint Summits on Translational Science* 2015, **2015**:36-40.
154. Yannakakis M: **Node-and edge-deletion NP-complete problems**. In: *Proceedings of the tenth annual ACM symposium on Theory of computing; San Diego, California, USA*. 804355: ACM 1978: 253-264.
155. Reed B, Smith K, Vetta A: **Finding odd cycle transversals**. *Operations Research Letters* 2004, **32**(4):299-301.

156. Hüffner F: **Algorithm Engineering for Optimal Graph Bipartization**. In: *Experimental and Efficient Algorithms: 4th International Workshop, WEA 2005, Santorini Island, Greece, May 10-13, 2005 Proceedings.* Edited by Nikoletseas SE. Berlin, Heidelberg: Springer Berlin Heidelberg; 2005: 240-252.

157. Guo J, Gramm J, Hüffner F, Niedermeier R, Wernicke S: **Compression-based fixed-parameter algorithms for feedback vertex set and edge bipartization**. *Journal of Computer and System Sciences* 2006, **72**(8):1386-1396.

158. Hopcroft JE, Karp RM: **An $n^{5/2} $ Algorithm for Maximum Matchings in Bipartite Graphs**. *SIAM Journal on Computing* 1973, **2**(4):225-231.

159. Fredman ML, Tarjan RE: **Fibonacci heaps and their uses in improved network optimization algorithms**. *J ACM* 1987, **34**(3):596-615.

160. Valiant LG: **The complexity of enumeration and reliability problems**. *SIAM Journal on Computing* 1979, **8**(3):410-421.

161. Gale D, Shapley LS: **College Admissions and the Stability of Marriage**. *The American Mathematical Monthly* 1962, **69**(1):9-15.

162. Irving RW: **Stable marriage and indifference**. *Discrete Applied Mathematics* 1994, **48**(3):261-272.

163. Iwama K, Miyazaki S: **A Survey of the Stable Marriage Problem and Its Variants**. In: *Proceedings of the International Conference on Informatics Education and Research for Knowledge-Circulating Society (icks 2008)*. 1345763: IEEE Computer Society 2008: 131-136.

164. Halford TR, Chugg KM: **An algorithm for counting short cycles in bipartite graphs**. *IEEE Transactions on Information Theory* 2006, **52**(1):287-292.

165. Steiner G: **On the k-path partition of graphs**. *Theoretical Computer Science* 2003, **290**(3):2147-2155.

166. Garey MR, Johnson DS: **Computers and Intractability: A Guide to the Theory of NP-Completeness**: W. H. Freeman \& Co.; 1979.

167. Peeters R: **The maximum edge biclique problem is NP-complete**. *Discrete Appl Math* 2003, **131**(3):651-654.

168. Di Giacomo E, Grilli L, Liotta G: **Drawing bipartite graphs on two curves**. In: *International Symposium on Graph Drawing: 2006*. Springer: 380-385.

169. Di Battista G, Eades P, Tamassia R, Tollis IG: **Algorithms for drawing graphs: an annotated bibliography**. *Computational Geometry* 1994, **4**(5):235-282.

170. Fößmeier U, Kaufmann M: **Nice drawings for planar bipartite graphs**. In: *Italian Conference on Algorithms and Complexity: 1997*. Springer: 122-134.

171. Eades P, Whitesides S: **Drawing graphs in two layers**. *Theoretical Computer Science* 1994, **131**(2):361-374.

172. Misue K: **Drawing bipartite graphs as anchored maps**. In: *Proceedings of the 2006 Asia-Pacific Symposium on Information Visualisation-Volume 60: 2006*. Australian Computer Society, Inc.: 169-177.

173. Misue K: **Anchored maps: Visualization techniques for drawing bipartite graphs**. In: *International Conference on Human-Computer Interaction: 2007*. Springer: 106-114.

174. Shahrokhi F, Sýkora O, Székely LA, Vrto I: **On bipartite drawings and the linear arrangement problem**. *SIAM Journal on Computing* 2001, **30**(6):1773-1789.

175. Eades P, Wormald NC: **Edge crossings in drawings of bipartite graphs**. *Algorithmica* 1994, **11**(4):379-403.

176. Zheng L, Song L, Eades P: **Crossing minimization problems of drawing bipartite graphs in two clusters**. In: *proceedings of the 2005 Asia-Pacific symposium on Information visualisation-Volume 45: 2005*. Australian Computer Society, Inc.: 33-37.

177. Valls V, Martí R, Lino P: **A branch and bound algorithm for minimizing the number of crossing arcs in bipartite graphs**. *European journal of operational research* 1996, **90**(2):303-319.
178. Callaway DS, Newman MEJ, Strogatz SH, Watts DJ: **Network Robustness and Fragility: Percolation on Random Graphs**. *Physical Review Letters* 2000, **85**(25):5468-5471.
179. Li D, Zhang Q, Zio E, Havlin S, Kang R: **Network reliability analysis based on percolation theory**. *Reliability Engineering & System Safety* 2015, **142**:556-562.
180. Hooyberghs H, Van Schaeybroeck B, Indekeu JO: **Percolation on bipartite scale-free networks**. *Physica A: Statistical Mechanics and its Applications* 2010, **389**(15):2920-2929.
181. Lü L, Zhou T: **Link prediction in complex networks: A survey**. *Physica A: Statistical Mechanics and its Applications* 2011, **390**(6):1150-1170.
182. Kunegis J, De Luca EW, Albayrak S: **The Link Prediction Problem in Bipartite Networks**. In: *Computational Intelligence for Knowledge-Based Systems Design: 13th International Conference on Information Processing and Management of Uncertainty, IPMU 2010, Dortmund, Germany, June 28 - July 2, 2010 Proceedings.* Edited by Hüllermeier E, Kruse R, Hoffmann F. Berlin, Heidelberg: Springer Berlin Heidelberg; 2010: 380-389.
183. Benchettara N, Kanawati R, Rouveirol C: **Supervised Machine Learning Applied to Link Prediction in Bipartite Social Networks**. In: *2010 International Conference on Advances in Social Networks Analysis and Mining: 9-11 Aug. 2010 2010*. 326-330.
184. Allali O, Magnien C, Latapy M: **Link prediction in bipartite graphs using internal links and weighted projection**. In: *2011 IEEE Conference on Computer Communications Workshops (INFOCOM WKSHPS): 10-15 April 2011 2011*. 936-941.
185. Agarwal S: **Learning to rank on graphs**. *Machine learning* 2010, **81**(3):333-357.
186. He X, Gao M, Kan M-Y, Wang D: **Birank: Towards ranking on bipartite graphs**. *IEEE Transactions on Knowledge and Data Engineering* 2017, **29**(1):57-71.
187. Wang JJ-Y, Bensmail H, Gao X: **Multiple graph regularized protein domain ranking**. *BMC bioinformatics* 2012, **13**(1):307.
188. Phillips CA: **Multipartite Graph Algorithms for the Analysis of Heterogeneous Data**. 2015.
189. Phillips CA, Wang K, Bubier J, Baker EJ, Chesler EJ, Langston MA: **Scalable multipartite subgraph enumeration for integrative analysis of heterogeneous experimental functional genomics data**. In: *Proceedings of the 6th ACM Conference on Bioinformatics, Computational Biology and Health Informatics: 2015*. ACM: 626-633.
190. Fortunato S: **Community detection in graphs**. *Physics Reports* 2010, **486**(3–5):75-174.
191. Sawardecker EN, Amundsen CA, Sales-Pardo M, Amaral LAN: **Comparison of methods for the detection of node group membership in bipartite networks**. *Eur Phys J B* 2009, **72**(4):671-677.
192. Barber MJ: **Modularity and community detection in bipartite networks**. *Physical Review E* 2007, **76**(6):066102.
193. Girvan M, Newman MEJ: **Community structure in social and biological networks**. *Proceedings of the National Academy of Sciences* 2002, **99**(12):7821-7826.
194. Larremore DB, Clauset A, Jacobs AZ: **Efficiently inferring community structure in bipartite networks**. *Physical Review E* 2014, **90**(1):012805.
195. Suzuki K, Wakita K: **Extracting Multi-facet Community Structure from Bipartite Networks**. In: *2009 International Conference on Computational Science and Engineering: 29-31 Aug. 2009 2009*. 312-319.

196. Liu X, Murata T: **Community Detection in Large-Scale Bipartite Networks**. In: *2009 IEEE/WIC/ACM International Joint Conference on Web Intelligence and Intelligent Agent Technology: 15-18 Sept. 2009 2009*. 50-57.
197. Du N, Wang B, Wu B, Wang Y: **Overlapping Community Detection in Bipartite Networks**. In: *2008 IEEE/WIC/ACM International Conference on Web Intelligence and Intelligent Agent Technology: 9-12 Dec. 2008 2008*. 176-179.
198. Dormann CF, Strauss R: **A method for detecting modules in quantitative bipartite networks**. *Methods in Ecology and Evolution* 2014, **5**(1):90-98.
199. Busygin S, Prokopyev O, Pardalos PM: **Biclustering in data mining**. *Comput Oper Res* 2008, **35**(9):2964-2987.
200. Prelić A, Bleuler S, Zimmermann P, Wille A, Bühlmann P, Gruissem W, Hennig L, Thiele L, Zitzler E: **A systematic comparison and evaluation of biclustering methods for gene expression data**. *Bioinformatics* 2006, **22**(9):1122-1129.
201. Liu Y-Y, Slotine J-J, Barabasi A-L: **Controllability of complex networks**. *Nature* 2011, **473**(7346):167-173.
202. Nacher JC, Akutsu T: **Structural controllability of unidirectional bipartite networks**. *Scientific Reports* 2013, **3**:1647.
203. Kontou PI, Pavlopoulou A, Dimou NL, Pavlopoulos GA, Bagos PG: **Data and programs in support of network analysis of genes and their association with diseases**. *Data in Brief* 2016, **8**:1036-1039.
204. Csardi G, Nepusz T: **The igraph software package for complex network research. InterJournal Complex Systems 1695**. *Available at igraph org/ Accessed November* 2006, **30**:2015.
205. Csardi G, Nepusz T: **The igraph software package for complex network research**. *InterJournal, Complex Systems* 2006, **1695**(5):1-9.
206. **BiLayout - Cytoscape plugin. http://bilayout.bioinf.mpi-inf.mpg.de/index.php. Accesed 15 Jan 2018**.
207. **PowerClust. http://www.compgen.org/tools/powerclust. Accessed 15 Jan 2018**.
208. Pundir S, Martin MJ, O'Donovan C: **UniProt Protein Knowledgebase**. *Methods in molecular biology* 2017, **1558**:41-55.
209. Finn RD, Coggill P, Eberhardt RY, Eddy SR, Mistry J, Mitchell AL, Potter SC, Punta M, Qureshi M, Sangrador-Vegas A *et al*: **The Pfam protein families database: towards a more sustainable future**. *Nucleic acids research* 2016, **44**(D1):D279-285.
210. Baxevanis AD: **The importance of biological databases in biological discovery**. *Current protocols in bioinformatics* 2011, **Chapter 1**:Unit 1 1.
211. Ng A, Bursteinas B, Gao Q, Mollison E, Zvelebil M: **Resources for integrative systems biology: from data through databases to networks and dynamic system models**. *Briefings in bioinformatics* 2006, **7**(4):318-330.
212. van Gend C, Snoep JL: **Systems biology model databases and resources**. *Essays in biochemistry* 2008, **45**:223-236.
213. Bebek G, Koyuturk M, Price ND, Chance MR: **Network biology methods integrating biological data for translational science**. *Briefings in bioinformatics* 2012, **13**(4):446-459.
214. Altaf-Ul-Amin M, Afendi FM, Kiboi SK, Kanaya S: **Systems biology in the context of big data and networks**. *BioMed research international* 2014, **2014**:428570.
215. Wang J, Pang GS, Chong SS, Lee CG: **SNP web resources and their potential applications in personalized medicine**. *Current drug metabolism* 2012, **13**(7):978-990.
216. Hoeppner MP, Barquist LE, Gardner PP: **An introduction to RNA databases**. *Methods in molecular biology* 2014, **1097**:107-123.

217. Bastone A, Diomede L, Parini R, Carnevale F, Salmona M: **Determination of argininosuccinate lyase and arginase activities with an amino acid analyzer**. *Analytical biochemistry* 1990, **191**(2):384-389.

218. Ooi HS, Schneider G, Chan YL, Lim TT, Eisenhaber B, Eisenhaber F: **Databases of protein-protein interactions and complexes**. *Methods in molecular biology* 2010, **609**:145-159.

219. Ooi HS, Schneider G, Lim TT, Chan YL, Eisenhaber B, Eisenhaber F: **Biomolecular pathway databases**. *Methods in molecular biology* 2010, **609**:129-144.

220. He Y, Xiang Z: **Databases and in silico tools for vaccine design**. *Methods in molecular biology* 2013, **993**:115-127.

221. Timmers LF, Pauli I, Caceres RA, de Azevedo WF, Jr.: **Drug-binding databases**. *Current drug targets* 2008, **9**(12):1092-1099.

222. Gozalbes R, Pineda-Lucena A: **Small molecule databases and chemical descriptors useful in chemoinformatics: an overview**. *Combinatorial chemistry & high throughput screening* 2011, **14**(6):548-458.

223. Pavlopoulou A, Spandidos DA, Michalopoulos I: **Human cancer databases (review)**. *Oncol Rep* 2015, **33**(1):3-18.

224. Ioannidis JP: **Why most published research findings are false**. *PLoS Med* 2005, **2**(8):e124.

225. Ioannidis JP, Ntzani EE, Trikalinos TA, Contopoulos-Ioannidis DG: **Replication validity of genetic association studies**. *Nat Genet* 2001, **29**(3):306-309.

226. Pavlopoulos GA, Paez-Espino D, Kyrpides NC, Iliopoulos I: **Empirical Comparison of Visualization Tools for Larger-Scale Network Analysis**. *Adv Bioinformatics* 2017, **2017**:1278932.

227. Martin S, Brown WM, Klavans R, Boyack KW: **OpenOrd: an open-source toolbox for large graph layout**. In*: 2011*. 786806-786806-786811.

228. Yifan H: **Efficient, High-Quality Force-Directed Graph Drawing**. *The Mathematica Journal* 2006, **10**(1).

229. Morris JH, Apeltsin L, Newman AM, Baumbach J, Wittkop T, Su G, Bader GD, Ferrin TE: **clusterMaker: a multi-algorithm clustering plugin for Cytoscape**. *BMC Bioinformatics* 2011, **12**:436.

230. Newman AM, Cooper JB: **AutoSOME: a clustering method for identifying gene expression modules without prior knowledge of cluster number**. *BMC Bioinformatics* 2010, **11**:117.

231. Eisen MB, Spellman PT, Brown PO, Botstein D: **Cluster analysis and display of genome-wide expression patterns**. *Proceedings of the National Academy of Sciences of the United States of America* 1998, **95**(25):14863-14868.

232. Frey BJ, Dueck D: **Clustering by passing messages between data points**. *Science* 2007, **315**(5814):972-976.

233. Newman ME, Girvan M: **Finding and evaluating community structure in networks**. *Physical review E, Statistical, nonlinear, and soft matter physics* 2004, **69**(2 Pt 2):026113.

234. Bader GD, Hogue CW: **An automated method for finding molecular complexes in large protein interaction networks**. *BMC Bioinformatics* 2003, **4**:2.

235. Enright AJ, Van Dongen S, Ouzounis CA: **An efficient algorithm for large-scale detection of protein families**. *Nucleic acids research* 2002, **30**(7):1575-1584.

236. Nepusz T, Sasidharan R, Paccanaro A: **SCPS: a fast implementation of a spectral method for detecting protein families on a genome-wide scale**. *BMC Bioinformatics* 2010, **11**:120.

237. Wittkop T, Emig D, Lange S, Rahmann S, Albrecht M, Morris JH, Bocker S, Stoye J, Baumbach J: **Partitioning biological data with transitivity clustering**. *Nature methods* 2010, **7**(6):419-420.

238. Pavlopoulos GA, Wegener AL, Schneider R: **A survey of visualization tools for biological network analysis**. *BioData mining* 2008, **1**:12.

239. Pavlopoulos GA, Malliarakis D, Papanikolaou N, Theodosiou T, Enright AJ, Iliopoulos I: **Visualizing genome and systems biology: technologies, tools, implementation techniques and trends, past, present and future**. *GigaScience* 2015, **4**:38.

240. Gehlenborg N, O'Donoghue SI, Baliga NS, Goesmann A, Hibbs MA, Kitano H, Kohlbacher O, Neuweger H, Schneider R, Tenenbaum D *et al*: **Visualization of omics data for systems biology**. *Nature methods* 2010, **7**(3 Suppl):S56-68.

241. Pavlopoulos GA, Iacucci E, Iliopoulos I, Bagos P: **Interpreting the Omics 'era' Data**. In: *Multimedia Services in Intelligent Environments: Recommendation Services.* Edited by Tsihrintzis GA, Virvou M, Jain LC. Heidelberg: Springer International Publishing; 2013: 79-100.

242. Shannon P, Markiel A, Ozier O, Baliga NS, Wang JT, Ramage D, Amin N, Schwikowski B, Ideker T: **Cytoscape: a software environment for integrated models of biomolecular interaction networks**. *Genome Res* 2003, **13**(11):2498-2504.

243. Saito R, Smoot ME, Ono K, Ruscheinski J, Wang PL, Lotia S, Pico AR, Bader GD, Ideker T: **A travel guide to Cytoscape plugins**. *Nature methods* 2012, **9**(11):1069-1076.

244. Piñero J, Bravo À, Queralt-Rosinach N, Gutiérrez-Sacristán A, Deu-Pons J, Centeno E, García-García J, Sanz F, Furlong LI: **DisGeNET: a comprehensive platform integrating information on human disease-associated genes and variants**. *Nucleic acids research* 2017, **45**(D1):D833-D839.

245. Batagelj V, Mrvar A: **Pajek-program for large network analysis**. *Connections* 1998, **21**(2):47-57.

246. Hagberg A, Schult D, Swart P: **{Exploring network structure, dynamics, and function using NetworkX}**. In: *{SciPy 2008: Proceedings of the 7th Python in Science Conference}: 2008*. 11-15.

247. Borgatti SP, Everett MG, Freeman LC: **Ucinet for Windows: Software for social network analysis**. 2002.

248. Bastian M, Heymann S, Jacomy M: **Gephi: An Open Source Software for Exploring and Manipulating Networks**; 2009.

249. Beckett S, Boulton C, Williams H: **FALCON: a software package for analysis of nestedness in bipartite networks [version 1; referees: 2 approved]**, vol. 3; 2014.

250. Secrier M, Pavlopoulos GA, Aerts J, Schneider R: **Arena3D: visualizing time-driven phenotypic differences in biological systems**. *BMC Bioinformatics* 2012, **13**:45.

251. Pavlopoulos GA, O'Donoghue SI, Satagopam VP, Soldatos TG, Pafilis E, Schneider R: **Arena3D: visualization of biological networks in 3D**. *BMC systems biology* 2008, **2**:104.

252. Barkow S, Bleuler S, Preli A, #263, Zimmermann P, Zitzler E: **BicAT: a biclustering analysis toolbox**. *Bioinformatics* 2006, **22**(10):1282-1283.

253. Baker EJ, Jay JJ, Bubier JA, Langston MA, Chesler EJ: **GeneWeaver: a web-based system for integrative functional genomics**. *Nucleic acids research* 2012, **40**(Database issue):D1067-D1076.

254. Neal Z: **ONEMODE: Stata module to produce one-mode projections of a bipartite network**. In.; 2015.

255. Krzywinski M, Schein J, Birol I, Connors J, Gascoyne R, Horsman D, Jones SJ, Marra MA: **Circos: an information aesthetic for comparative genomics**. *Genome research* 2009, **19**(9):1639-1645.

256. Krzywinski M, Birol I, Jones SJ, Marra MA: **Hive plots--rational approach to visualizing networks**. *Briefings in bioinformatics* 2012, **13**(5):627-644.

257. Bikakis N, Liagouris J, Krommyda M, Papastefanatos G, Sellis T: **graphVizdb: A Scalable Platform for Interactive Large Graph Visualization**. *32nd IEEE International Conference on Data Engineering (ICDE '16)* 2016.
258. Kohler J, Baumbach J, Taubert J, Specht M, Skusa A, Ruegg A, Rawlings C, Verrier P, Philippi S: **Graph-based analysis and visualization of experimental results with ONDEX**. *Bioinformatics* 2006, **22**(11):1383-1390.
259. Iragne F, Nikolski M, Mathieu B, Auber D, Sherman D: **ProViz: protein interaction visualization and exploration**. *Bioinformatics* 2005, **21**(2):272-274.
260. Hu Z, Hung JH, Wang Y, Chang YC, Huang CL, Huyck M, DeLisi C: **VisANT 3.5: multi-scale network visualization, analysis and inference based on the gene ontology**. *Nucleic acids research* 2009, **37**(Web Server issue):W115-121.
261. Adar E: **GUESS: a language and interface for graph exploration**. In: *Proceedings of the SIGCHI Conference on Human Factors in Computing Systems; Montréal, Québec, Canada*. 1124889: ACM 2006: 791-800.
262. Borgatti SP, Everett MG, Freeman LC: **Ucinet for Windows: Software for Social Network Analysis**. *Harvard, MA: Analytic Technologies* 2002.
263. Thimm O, Blasing O, Gibon Y, Nagel A, Meyer S, Kruger P, Selbig J, Muller LA, Rhee SY, Stitt M: **MAPMAN: a user-driven tool to display genomics data sets onto diagrams of metabolic pathways and other biological processes**. *The Plant journal : for cell and molecular biology* 2004, **37**(6):914-939.
264. Demir E, Babur O, Dogrusoz U, Gursoy A, Nisanci G, Cetin-Atalay R, Ozturk M: **PATIKA: an integrated visual environment for collaborative construction and analysis of cellular pathways**. *Bioinformatics* 2002, **18**(7):996-1003.
265. Pavlopoulos GA, Hooper SD, Sifrim A, Schneider R, Aerts J: **Medusa: A tool for exploring and clustering biological networks**. *BMC research notes* 2011, **4**:384.
266. Breitkreutz BJ, Stark C, Tyers M: **Osprey: a network visualization system**. *Genome biology* 2003, **4**(3):R22.
267. Theocharidis A, van Dongen S, Enright AJ, Freeman TC: **Network visualization and analysis of gene expression data using BioLayout Express(3D)**. *Nature protocols* 2009, **4**(10):1535-1550.
268. Admiraal R, Handcock MS: **Networksis: a package to simulate bipartite graphs with fixed marginals through sequential importance sampling**. *Journal of Statistical Software* 2008, **24**(8).
269. Borrett SR, Lau MK: **enaR: An r package for Ecosystem Network Analysis**. *Methods in Ecology and Evolution* 2014, **5**(11):1206-1213.
270. Dormann CF, Gruber B, Fründ J: **Introducing the bipartite package: analysing ecological networks**. *interaction* 2008, **1**:0.2413793.
271. SEAL A, Wild DJ: **Netpredictor: R and Shiny package to perform Drug-Target Bipartite network analysis and prediction of missing links**. *bioRxiv* 2016.
272. Opsahl T: **Structure and evolution of weighted networks**. Queen Mary, University of London; 2009.
273. Gobbi A, Albanese D, Iorio F: **Package 'BiRewire'**. 2013.
274. Vrahatis AG, Balomenos P, Tsakalidis AK, Bezerianos A: **DEsubs: an R package for flexible identification of differentially expressed subpathways using RNA-seq experiments**. *Bioinformatics* 2016, **32**(24):3844-3846.
275. **SNAP library. https://snap.stanford.edu/data/index.html. Accessed Jan 2018**.
276. Leskovec J, Sosič R: **Snap: A general-purpose network analysis and graph-mining library**. *ACM Transactions on Intelligent Systems and Technology (TIST)* 2016, **8**(1):1.
277. **Colorado Index of Complex Networks (ICON). https://icon.colorado.edu/#!/. Accessed 15 Jan 2018**.

278. **Koblenz Network Collection (KONECT). http://konect.uni-koblenz.de/. Accessed 15 Jan 2018**.

279. Kunegis J: **Konect: the koblenz network collection**. In: *Proceedings of the 22nd International Conference on World Wide Web: 2013*. ACM: 1343-1350.

280. **Web of Life. http://www.web-of-life.es/. Accessed 15 Jan 2018**.

281. **Interaction Web Database. https://www.nceas.ucsb.edu/interactionweb/index.html. Accessed 15 Jan 2018**.

282. **Kelpforest Database. http://kelpforest.ucsc.edu/visualizations/. Accessed 15 Jan 2018**.

283. Beas-Luna R, Novak M, Carr MH, Tinker MT, Black A, Caselle JE, Hoban M, Malone D, Iles A: **An online database for informing ecological network models: http://kelpforest. ucsc. edu**. *PloS one* 2014, **9**(10):e109356.

284. Xu J: **INVITED REVIEW: Microbial ecology in the age of genomics and metagenomics: concepts, tools, and recent advances**. *Molecular Ecology* 2006, **15**(7):1713-1731.

285. Jones MB, Schildhauer MP, Reichman OJ, Bowers S: **The New Bioinformatics: Integrating Ecological Data from the Gene to the Biosphere**. *Annual Review of Ecology, Evolution, and Systematics* 2006, **37**(1):519-544.

286. Hrček J, Godfray HCJ: **What do molecular methods bring to host–parasitoid food webs?** *Trends in Parasitology*, **31**(1):30-35.

287. Proulx SR, Promislow DEL, Phillips PC: **Network thinking in ecology and evolution**. *Trends in Ecology & Evolution*, **20**(6):345-353.

Figure 1 Click here to download Figure Figure1.tif

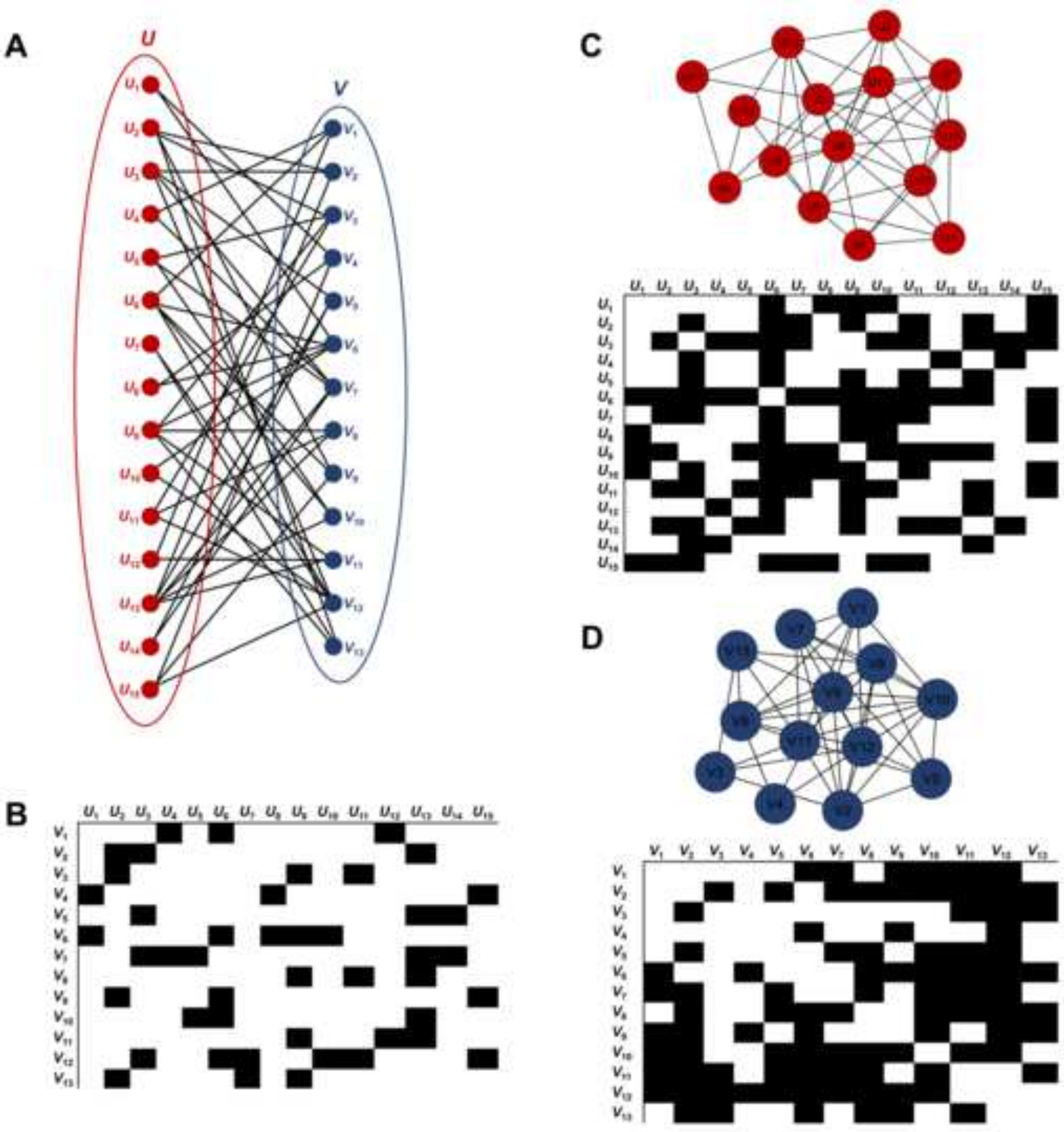

Figure 2

Click here to download Figure Figure2.tif

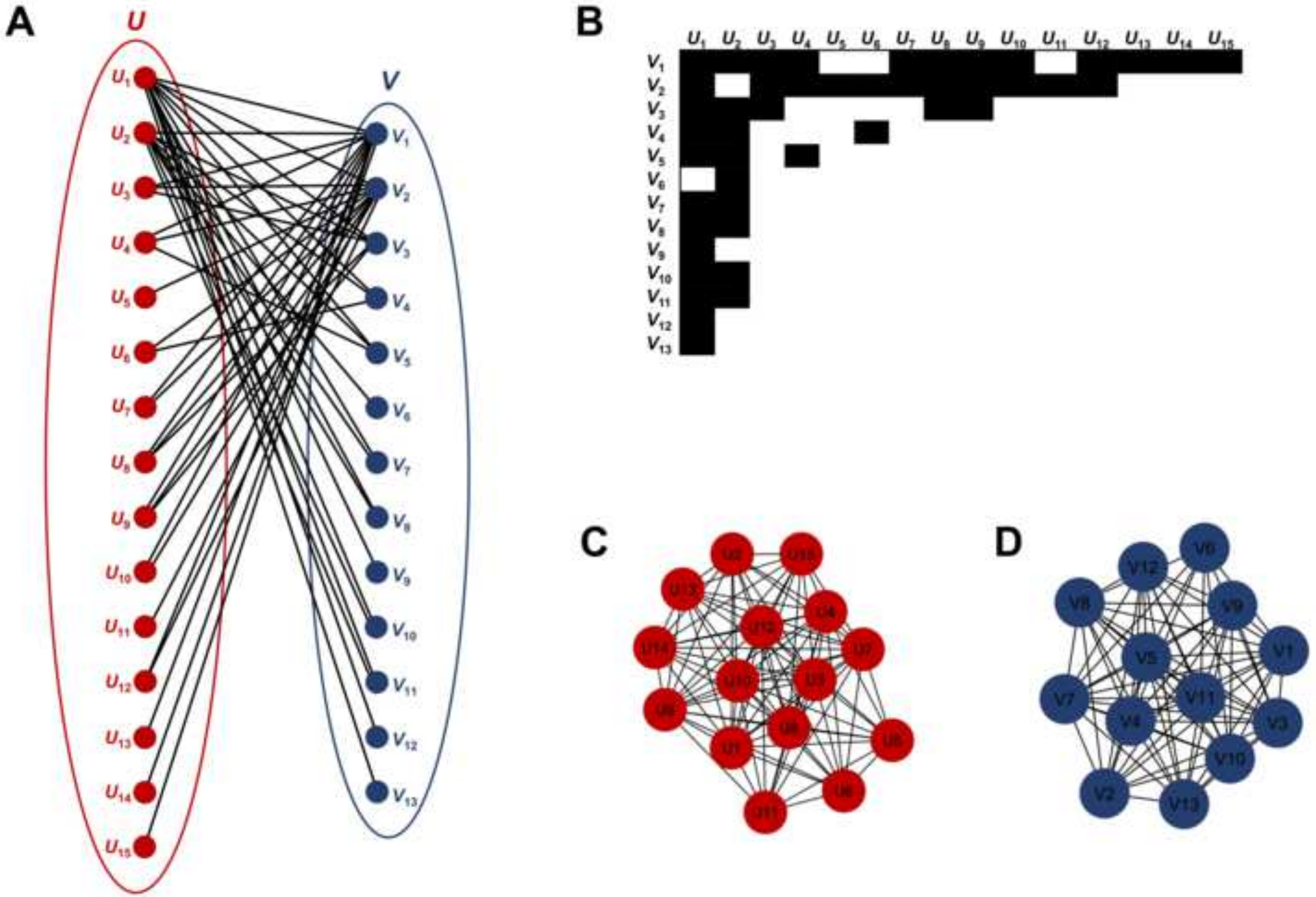

Figure 3 Click here to download Figure Figure3.tif

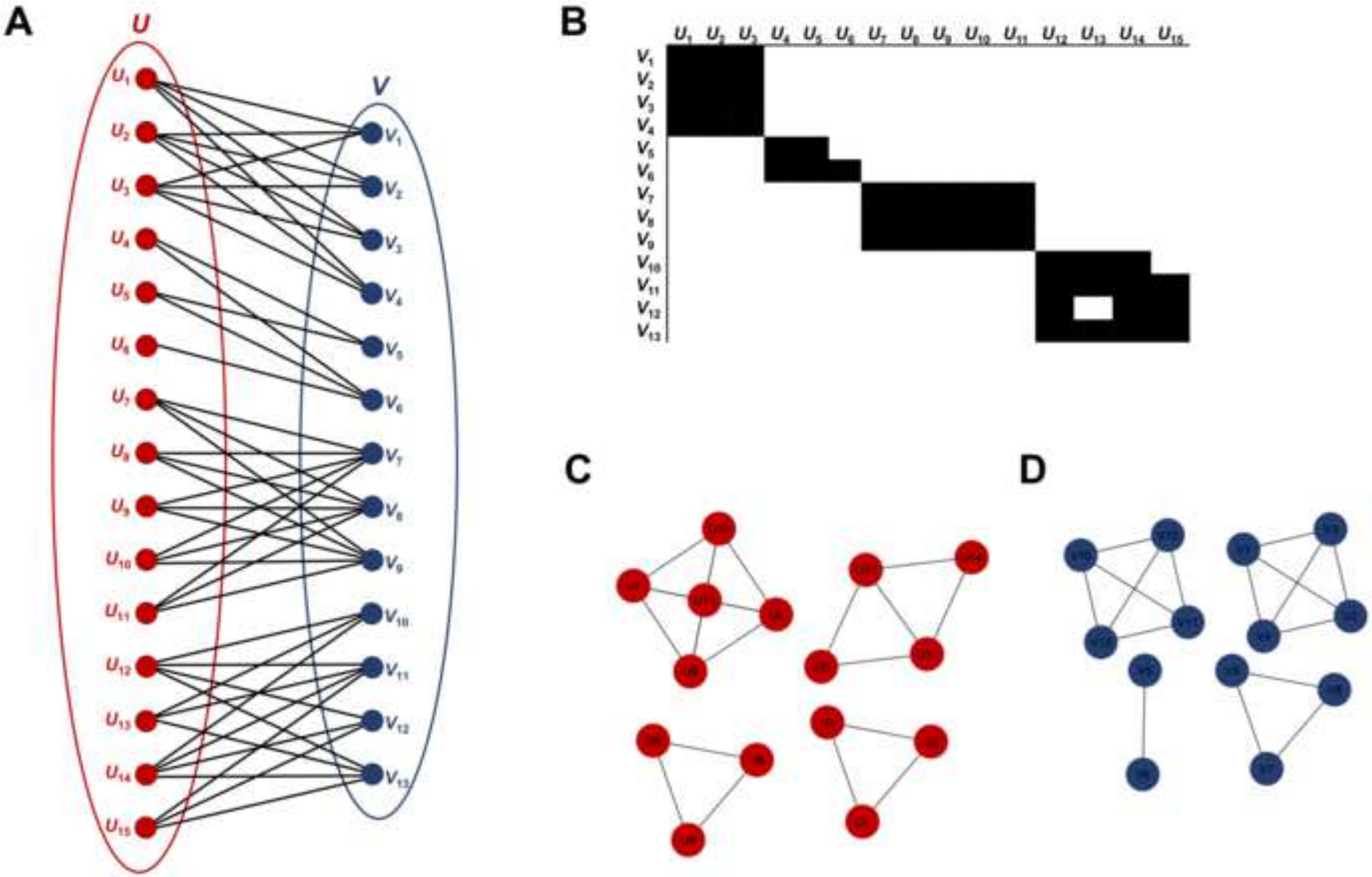

Figure 4

Click here to download Figure Figure4.tif

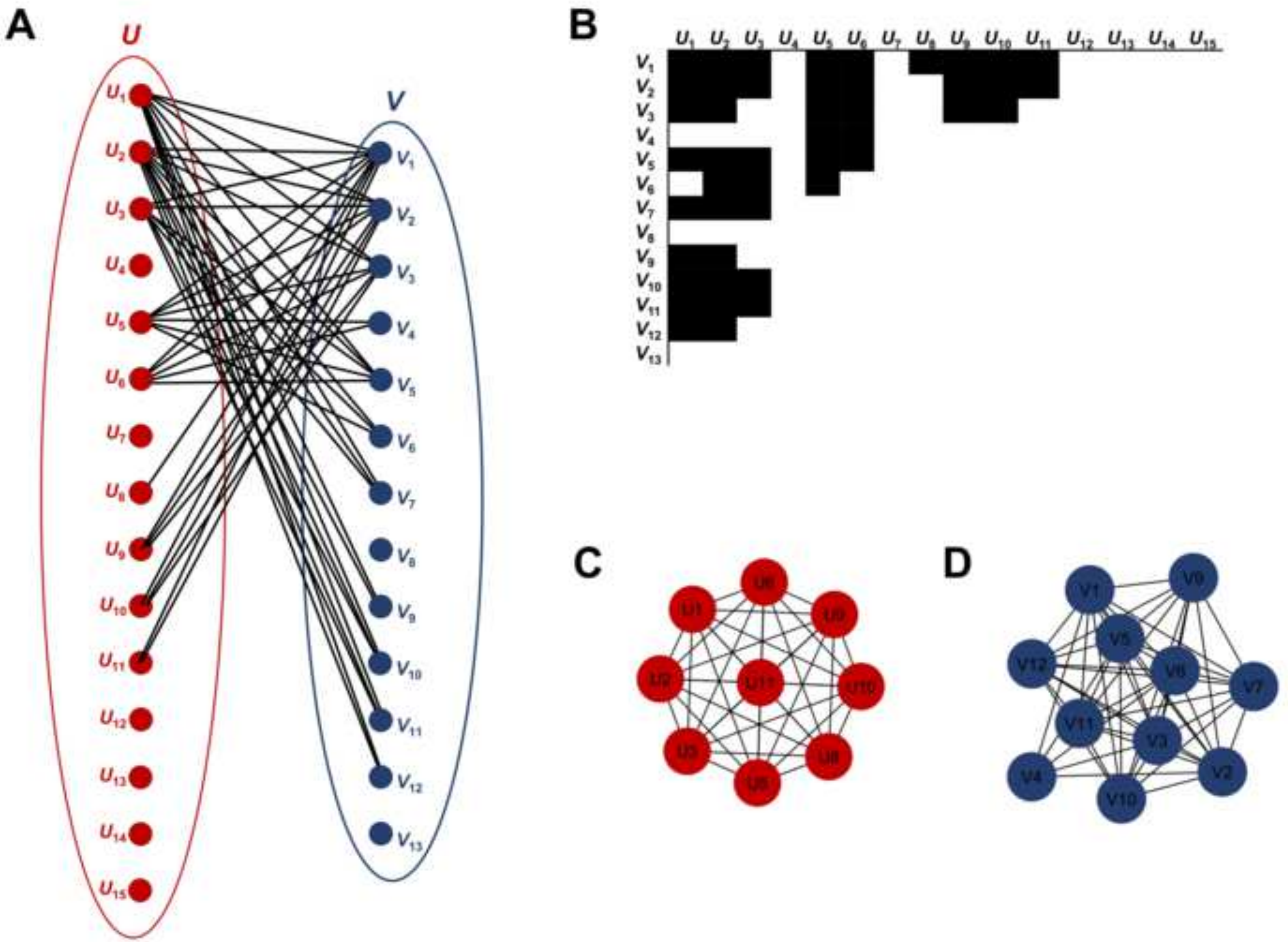

Figure 5

Click here to download Figure Fig_5_license_free.jpg

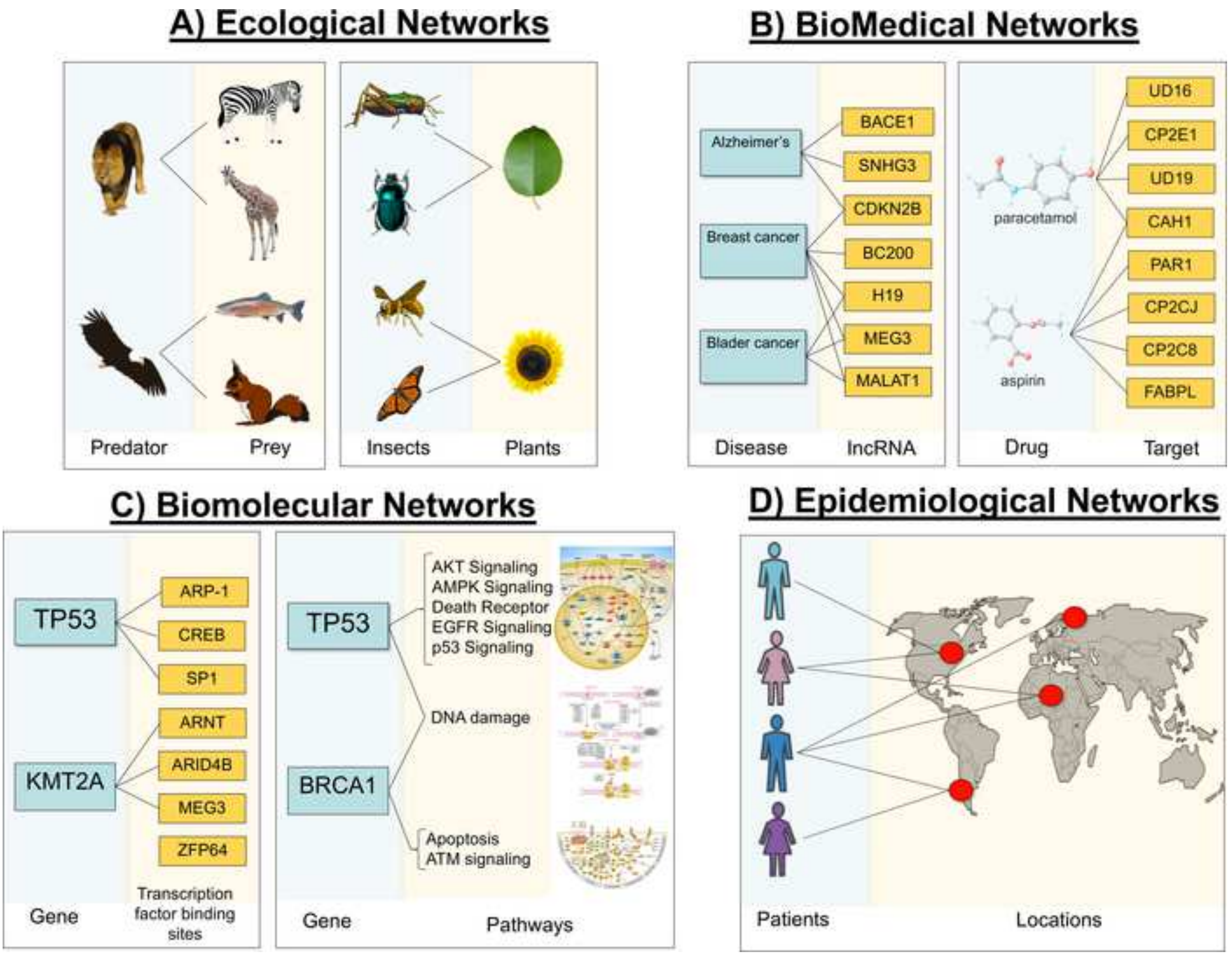

Figure 6 Click here to download Figure Figure6.jpg

A

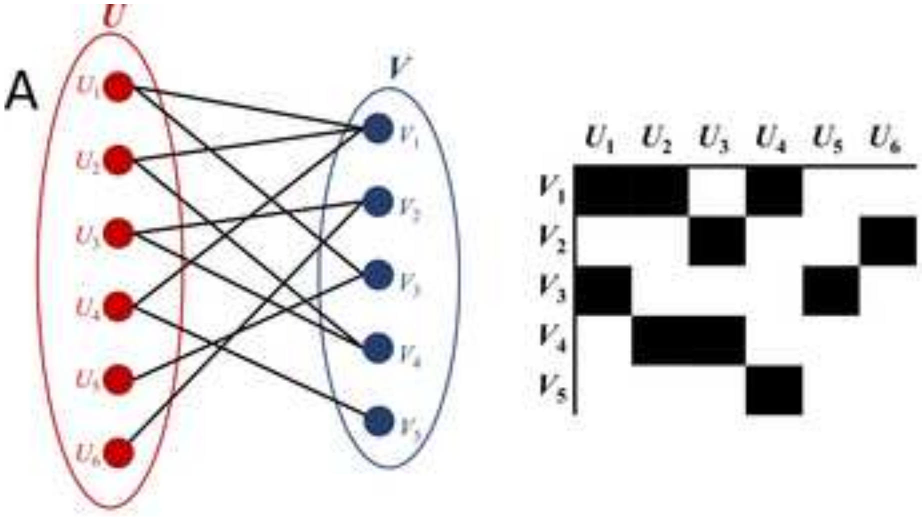

Number of Edges	10.00
Number of Nodes	11.00
Density	0.18
Average path length	3.45
Clustering Coefficient	0.00
Modularity	0.39
Centralization.betweenness	0.46
Centralization.closeness	0.26
Centralization.degree	0.12

	Dgr	BC
V1	3.00	31.00
U2	2.00	24.00
V4	2.00	21.00
U1	2.00	16.00
U3	2.00	16.00
U4	2.00	9.00
V3	2.00	9.00
V2	2.00	9.00
U5	1.00	0.00
U6	1.00	0.00

B

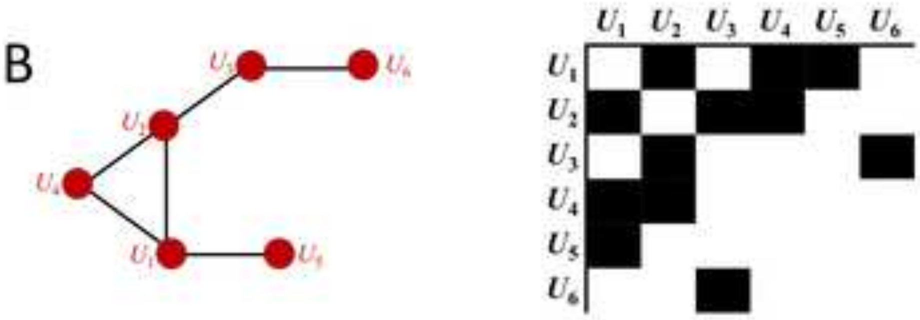

Number of Edges	6.00
Number of Nodes	6.00
Density	0.40
Average path length	1.93
Clustering Coefficient	0.38
Modularity	0.21
Centralization.betweenness	0.44
Centralization.closeness	0.47
Centralization.degree	0.20

	Dgr	BC
U2	3.00	6.00
U3	3.00	4.00
U1	2.00	4.00
U6	2.00	0.00
U4	1.00	0.00
U5	1.00	0.00

C

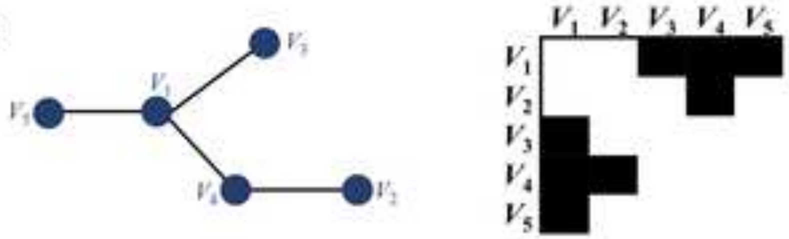

Number of Edges	4.00
Number of Nodes	5.00
Density	0.40
Average path length	1.80
Clustering Coefficient	0.00
Modularity	0.22
Centralization.betweenness	0.71
Centralization.closeness	0.64
Centralization.degree	0.35

	Dgr	BC
V1	3.00	5.00
V4	2.00	3.00
V2	1.00	0.00
V3	1.00	0.00
V5	1.00	0.00

Figure 7

Click here to download Figure Figure7.jpg

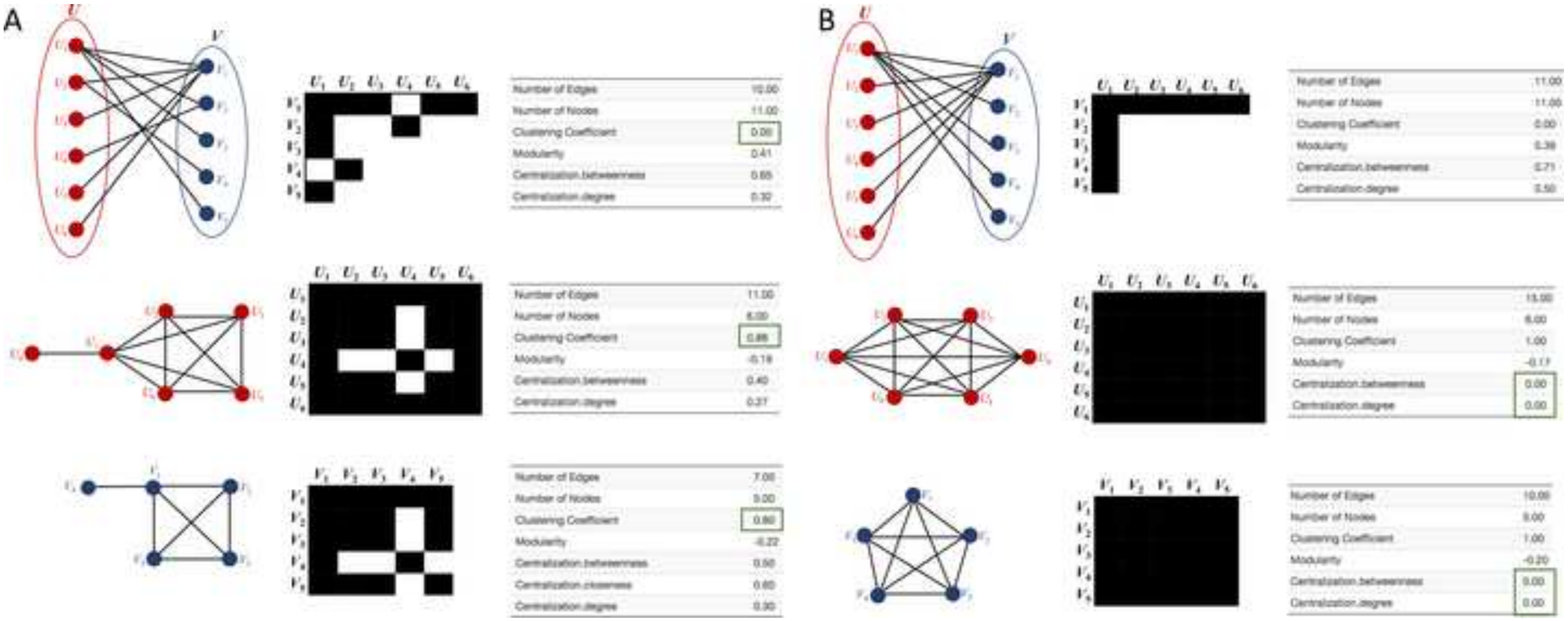

Figure 8 Click here to download Figure Figure8.jpg

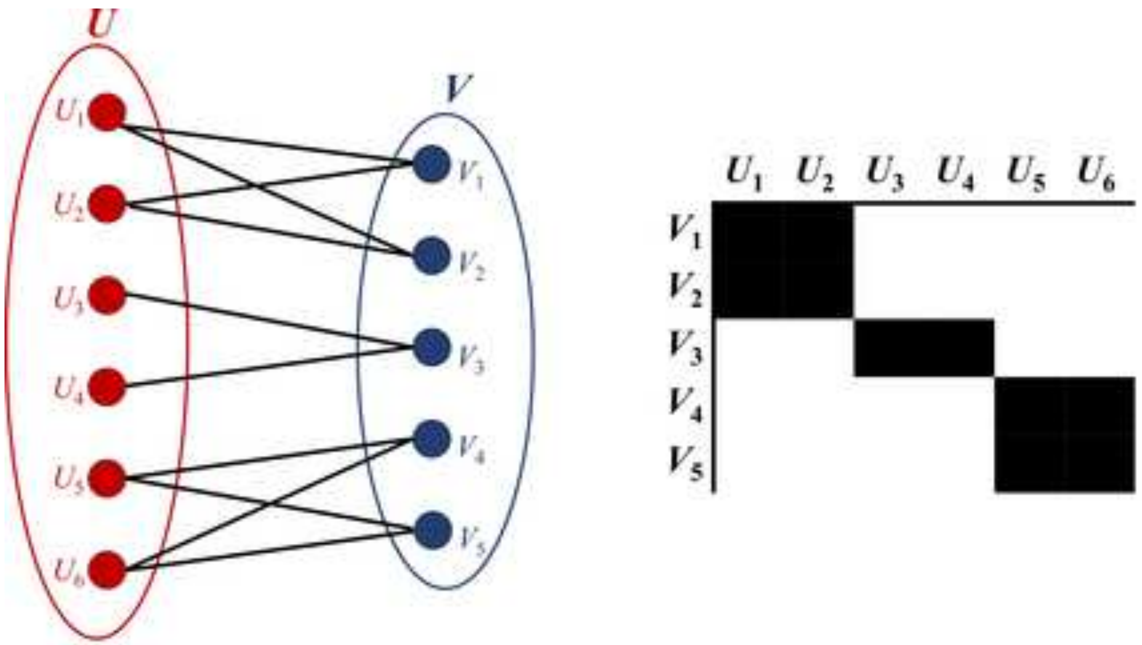

Number of Edges	10.00
Number of Nodes	11.00
Centralization.betweenness	0.01
Centralization.degree	0.02

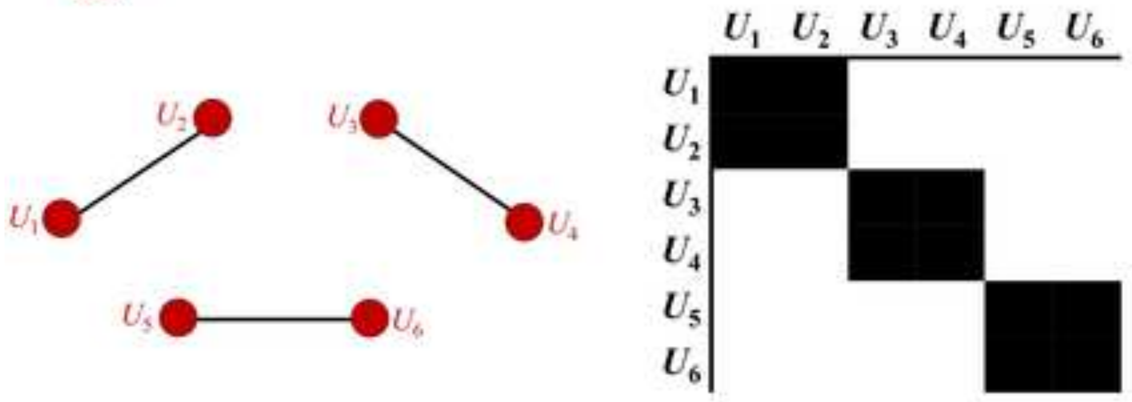

Number of Edges	3.00
Number of Nodes	6.00
Centralization.betweenness	0.00
Centralization.degree	0.00

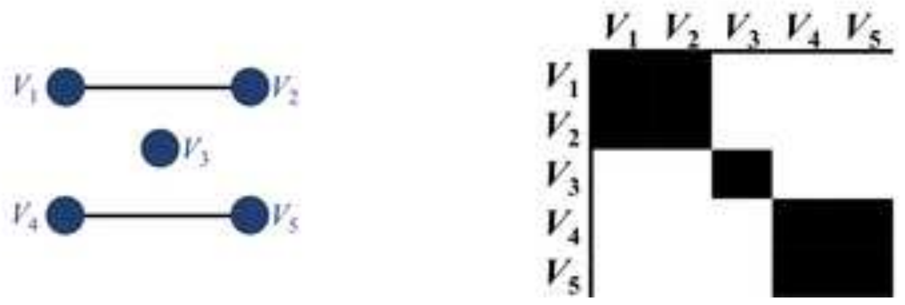

Number of Edges	2.00
Number of Nodes	4.00
Centralization.betweenness	0.00
Centralization.degree	0.00

Figure 9 Click here to download Figure Figure9.tif

A

Bipartite Network	Nodes	888 (791G, 98D)
	Edges	1023
	Radius	1.00000
	Density	0.00000
	Diameter	14.00000
	Modularity	0.86233
	Clustering Coefficient	0.00000
	Centralization Betweeness	0.25123
	Centralization Closeness	0.00000
	Linkage Density	1.21352
	Connectance	0.01915
	Generality	14.82609
	Vulnerability	1.32170

B

GENE	DISEASE
ABCD1	SCHIZOPHRENIA
ADAM30	NON-INSULIN DEPENDENT_DIABETES_MELLITUS
ADAMTS9	NON-INSULIN-DEPENDENT_DIABETES_MELLITUS
BIN1	ALZHEIMER'S_DISEASE
BMP4	MALIGNANT_NEOPLASM_OF_COLON
BOD1	CROHN'S_DISEASE
BPI	BIPOLAR_AFFECTIVE_DISORDER
BPI	SCHIZOPHRENIA
CCDC62	PARKINSON'S_DISEASE
FIGN	HYPERTENSION
RPL17P45	OBESITY
ST13P4	MULTIPLE_SCLEROSIS
ZNF646	PARKINSON'S_DISEASE
ZMIZ1	CROHN'S_DISEASE
ZMIZ1	MULTIPLE_SCLEROSIS
ST13P1	NON-INSULIN - DEPENDENT_DIABETES_MELLITUS
ST13P1	SCHIZOPHRENIA

C

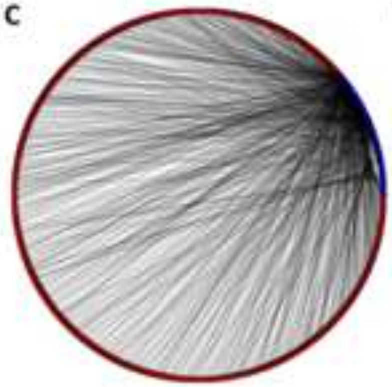

D

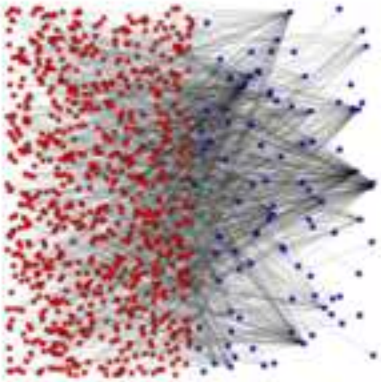

E

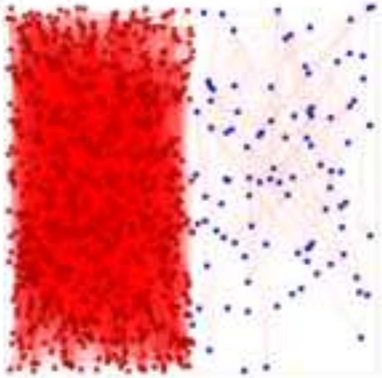

F

Disease-Disease network	Nodes	69
	Edges	168
	Radius	1.00000
	Density	0.07214
	Diameter	6.00000
	Clustering Coefficient	0.44321
	Centralization Betweeness	0.21000
	Centralization Closeness	0.00414

CROHN'S_DISEASE	MULTIPLE_SCLEROSIS
BIPOLAR_AFFECTIVE_DISORDER	SCHIZOPHRENIA
NON-INSULIN-DEPENDENT_DIABETES_MELLITUS	SCHIZOPHRENIA

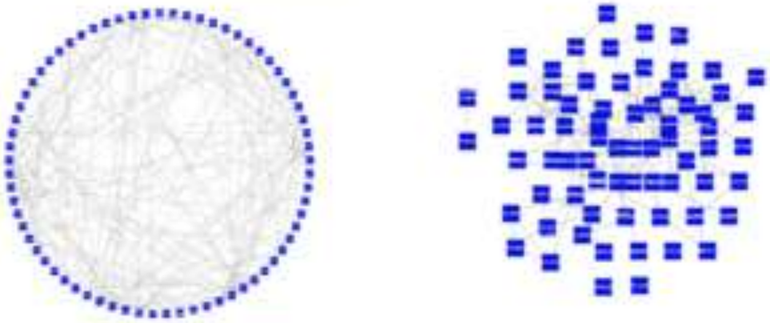

G

Gene-Gene network	Nodes	774
	Edges	14903
	Radius	1.00000
	Density	0.03843
	Diameter	10.00000
	Clustering Coefficient	0.73574
	Centralization Betweeness	0.16384
	Centralization Closeness	0.00000

ADAM30	ADAMTS9
BOD1	ZMIZ1
ABCD1	BPI
CCDC62	ZNF646
BPI	ST13P1
ABCD1	ST13P1
ADAM30	ST13P1

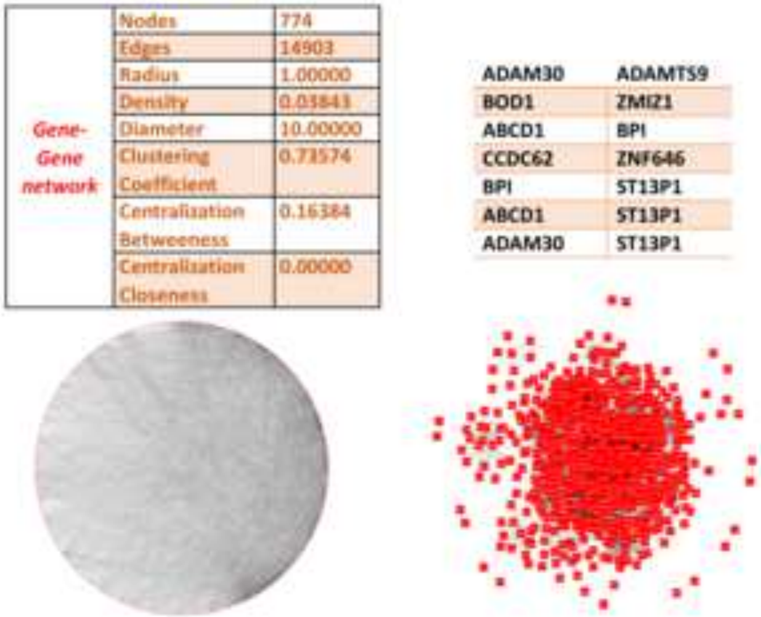

Figure 10

Click here to download Figure Figure10.jpg

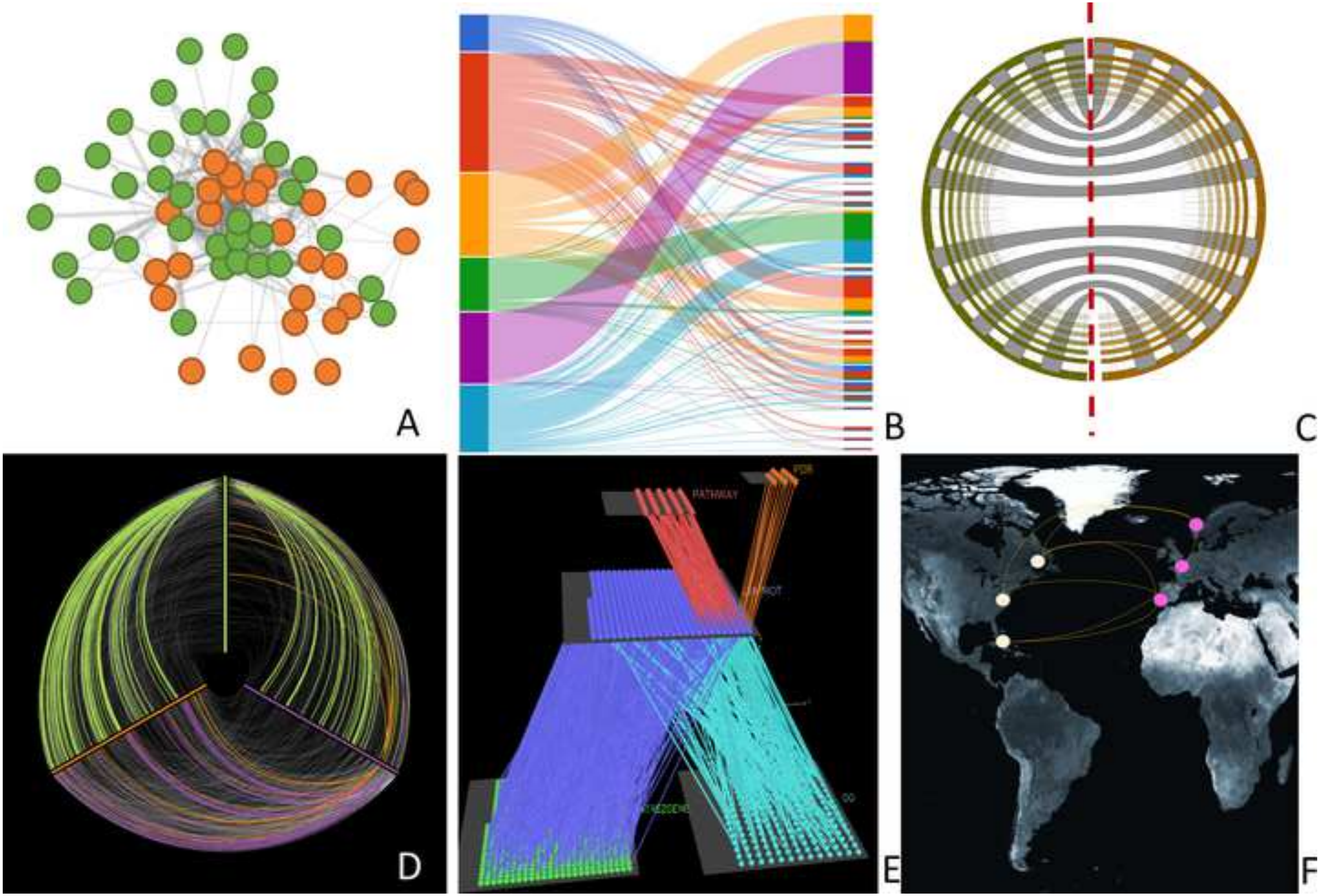

Click here to access/download
Supplementary Material
supplementary_material.xlsx
