# Supplementary material for: Bipartite graphs in systems biology and medicine: a survey of methods and applications
Source: Gigascience. 2018 Feb 19;7(4):giy014. doi: 10.1093/gigascience/giy014 (PMC6333914; doi:10.1093/gigascience/giy014)
Supplement: Response_to_Reviewer_Comments_Original_Submission.pdf [file giy014_response_to_reviewer_comments_original_submission.pdf]

Author's Response To Reviewer Comments

Dear Editor,

We would like to thank the reviewers for their useful feedback and constructive comments and the editor for offering us the opportunity to improve the overall quality of our manuscript.

Herein, we are submitting a revised version of the manuscript now entitled “Bipartite Graphs in Systems Biology and Medicine: a survey of methods and applications” to be considered for publication in GigaScience journal.

Below you can find our point-by-point responses addressing the reviewers’ comments. We hope that this version will satisfy all the concerns raised and we are looking forward to hearing from you.

Sincerely,

Pantelis Bagos, on behalf of the authors

Editor’s comments
Comment: I somewhat agree with reviewer 1 that you could think a bit more about the intended goals of your manuscript, and focus the text more with that in mind. That said, as your manuscript is submitted as a Review Article, a "survey paper of the applications of bipartite graphs in network biology" (reviewer 1) is actually fine with us, in principle, if it provides useful information for our readers.

Response: We agree with the Editor and Reviewer 1 and therefore we updated both the text content and the title of the manuscript to better communicate what the main purpose of this article is. In this way we may enable both network experts and non-experts to explore potential applications of bipartite graphs within the BioMed territory. To do that, we first make sure that readers get familiar with the main concepts of the bipartite graphs, potential applications and tools offering related analyses and visualizations. To this end, we have made several changes in the manuscript which you can see as you go through our response to the reviewers. Now the manuscript’s title has been changed from Bipartite graphs in Systems Biology and Medicine to Bipartite graphs in Systems Biology and Medicine: a survey of methods and applications. The backbone of the article remained the same and is structured as follows:
A theoretical overview of the graph theory and definitions behind bipartite graphs and their properties is given.
The potential applications in Biology and Medicine are discussed.
The tool, datasets and the contemporary state-of-the-art software are discussed.
Provide useful insights and future perspectives
In the revised version, in order to put the manuscript in a Big-Data context, we introduced several new sections, while we always focus on the potential of bipartite graphs in Biology and Medicine, something which has not been presented before. We try to keep the balance between

being technical but also showing the big picture, hoping to make this article more appealing to a broader readership.

Comment: The reviewers mention that actual case studies / data sets and "future directions" could be covered in more depth, and I agree that discussing these aspects more prominently may be an area for improvement.

Response: We thank the Editor and the Reviewers for this suggestion. We expanded the text significantly by giving useful insights and future perspectives related to bipartite graph along with real examples. In order to address both comments we added two new sections:
1.Bipartite graphs, the BioMedical Big-Data era and hints for the future
In this section we initially give some statistics to describe the Big-Data era in the Bioworld. Once we make clear what are the urgencies here, we discuss how advanced analysis and visualization specialized in bipartite graphs could help.
2. Examples of bipartite network analysis
As it is difficult to exhaust all possible examples related to bipartite graphs in BioMedicine, we provide one real example from previously published work pointing to the merits of both analysis using the bipartite structure as well as using the projections. We also include several examples from artificially generated networks (see next comment)

Comment: In our experience, figures and tables from Review Articles such as yours are often very helpful and reused for educational purposes, in talks etc (facilitated by our open CC-BY licence). You already provide some good figures to illustrate the concepts, but feel free to add some more, if you wish. For example, you may also add a Table or figure summarizing the available software tools and their use areas, as presented in the main text.

Response: While Figures 1-5 remained intact, now, the newly added Figures 6,7 and 8 provide numerical examples using smaller graphs. Their purpose is to show how the topology of the initial bipartite graph can affect the topology of the two projected unipartite networks. Additionally, we enriched the text in order to discuss and highlight such patterns. Figure 9 is related to the case study on gene-disease network (previous comment). As indeed such figures can be used for educational purposes, we decided to add a new Figure (#10) showing how bi- and n-partite graphs can be visualized with available visualization tools. Unfortunately, there is a great of lack of visualization tools dedicated to bipartite networks, but here, we tried to show how already existing generic tools can be used in this context. Finally, as suggested by the Editor, we incorporated a table summarizing all available tools, their URLs as well as their use areas (Table 1).

Comment: Regarding non-biological use examples of bipartite graphs (point #1 of reviewer 2), I think this may lead too far away from our mainly biomedical audience - but if you wish, please feel free to mention it briefly (but I'd leave this up to you).

Response: We believe that the manuscript is already too crowded and that there is limited space for redirecting the text towards non-biomedical paths. Meanwhile, we find the suggestion useful as such articles are interdisciplinary and attract readers from other areas of expertise. Therefore, we added a brief but concise paragraph in the Introduction dedicated to non-biological examples

known in the literature.

Comment: On a minor note, please also correct a typo in fig. 1 ("prey", not "pray").

Response: We corrected the figure and checked the whole manuscript for other typos using a spell checker as suggested by Reviewer 2 too.

Reviewer 1:
Reviewer 1:
Comment: My main concern is that I am not sure whether this manuscript is relevant to the readers in the GigaScience community since it is more like a survey paper of the applications of bipartite graphs in network biology, or a textbook chapter or the lecture notes, to introduce bipartite graphs, evaluation measures, real-life networks, software and tools in related literatures.

Response: We thank the reviewer for expressing this concern, also commented by the Editor. Indeed GigaScience is more Big-Data oriented but we believe that after the extensive revision and additions our manuscript falls within the scope of the journal. Machine learning, visualization, information retrieval, data integration, network analysis and others, are complementary fields which often share cross-discipline concepts. The aim of this manuscript is to find a common ground among these different communities and provide a “common code” that everyone understands. That is why we try to avoid being either too technical or too biological as this would not appeal to readers with a more biomedical or more technical background, respectively. Taking into account the reviewer’s and Editor’s comment we modified the text accordingly and to a significant extend. Our top highlighted changes, among others, are the following:
We added new Figures with numerical examples (mostly for education purposes) on artificial datasets, as well as on a real example (case study) to make the bipartite graph theory part appealing to non-experts.
We explained how the topological features of a bipartite graph affect the properties of its two projected unipartite networks
We put the text more into a big-data context (see new section: Bipartite graphs, the BioMedical Big-Data era and some hints for the future) explaining what is the state-of-the-art and the gaps in the areas of comparative genomics and integrative biology. With this addition, we hope that now we do not deviate from the journal’s philosophy.
We added some real examples so that Bio-experts can understand the usefulness of bipartite graphs in a more obvious way
We added a new Figure showing how bipartite or n-partite graphs can be visualized with existing tools. We mention the lack of tools dedicated to the analysis of these types of networks, but we provide insights of how existing tools can be employed for this purpose.
We summarized the advantages and the disadvantages of the state-of-the-art tools in a concrete table.

Comment: If this paper is a research / theoretical - oriented paper, then it is better to propose the novel technical solutions on evaluation measures and analytics algorithms of bipartite graphs. If this paper is a practice / system - oriented paper, the systems on large-scale real-life networks should be developed and implemented to improve the scalability and efficiency of bipartite graph

analysis. If this paper is an experiment / case study - oriented paper, the experiments on either synthetic or real datasets should be conducted to compare the state-of-the -art tools' effectiveness and efficiency.

Response: We understand the reviewer's concern but here, we have made an effort to present a review paper covering many aspects of bipartite analysis within the Biomedical spectrum of interest and, at the same time, to address the needs of very diverse audiences of different backgrounds. It is not the purpose of this article to provide a very technical documentation of the bipartite graphs, neither to evaluate algorithms or benchmark the efficiency of the tools using benchmarking datasets. Please keep in mind that despite the fact that many advances have been made in the field of computational bipartite graph analysis, their applications in the BioMedical field are very poor. We updated the text to highlight this point and we also comment on the fact that systems on large-scale real-life networks should be developed and implemented to improve the scalability and efficiency of bipartite graph analysis. Deeper evaluation of algorithms as well as better documentation related to this type of networks is covered in other studies which are referenced in our paper. To our knowledge, this is the first article that puts the analysis of bipartite graphs into a Biomedical context and we expect to be greatly endorsed by the biomedical community once this is published. To do that the article was structured as follows:
Simplified mathematical definition of a bipartite graph
Theoretical properties and the importance of the projections along with potential biological applications of them
Real-life network types and how these can be analyzed using the graph theory related to bipartite graphs
Models and algorithms for bipartite graphs
Advantages of available software dedicated to analyze such networks.
Real case studies
Future perspectives
Non-biomedical cases

Comment: Graph matching, link prediction, and community detection / graph partitioning have been well studied in many literatures in network biology, database, data mining, and machine learning. The authors failed to identify and discuss many recent references in these fields.

Response: The current version of the manuscript already comes with >220 citations and we consciously prefered to reference some broader reviews rather than specific algorithms and methods, as this could lead to information overload. Nevertheless, we understand the reviewer's objection and without exaggerations, we digged further into the literature and expanded our citation list by discussing some of these aspects into more detail. Again, we voided being too technical as we want this article to be balanced and appealing for readers coming from various BioMedical related fields.

Comment: Overall, I think the journal manuscript has very limited research contributions compared with related literatures. Therefore I would suggest that the authors should seriously consider new aspects of the problems, e.g. adding new algorithms, new systems, or new experiments, to further improve the manuscript.

Response: Trying to satisfy readers from various areas such as Medicine, Biology and Computer Science and simultaneously trying to keep the balance between going into too much detail but not losing the presentation of the big picture is challenging. Our aim here is to compose an article demonstrating the potential of bipartite graphs in Biomedical case studies and we are sure that it will be very well-received from the broader community, something that we already experienced with other articles of ours of the same style in the past. Nevertheless, we understand the Reviewer's concern and therefore, following her/his comment, we updated the text, the figures, the literature, the future perspectives, the discussion and many other parts of the manuscript.

Reviewer 2
Comment: Several points could be addressed to improve the current version of this paper.
My suggestions are shown below:
1. The bipartite graph has been extensively used to model the relationship such as Query and URL [A] in query logs, Video shots and Tags [B], Entity and Co-List in a Web page [C], Users and Items in recommendation [D, E]. Some of these citations could be included in the paper.
A. A Generalized Co-HITS Algorithm and Its Application to Bipartite Graphs, Deng et al. (2009)
B. "Visualtextualrank: An extension of visual rank to large-scale video shot extraction exploiting tag co-occurrence, K. YANAI et al. (2015)
C. Bipartite graph based entity ranking for related entity finding, Cao et al. (2011)
D. Recommendation as link prediction in bipartite graphs: A graph kernel-based machine learning approach, Xin Li and Hsinchun Chen (2013)
E. BiRank: Towards Ranking on Bipartite Graphs, He et al. (2017)

Response: We thank the reviewer for this suggestion. We believe that the current article is already long enough so as to expand it further and discuss the applicability of bipartite graphs in non biomedical areas. This is definitely something that could be covered independently in a separate article exclusively dedicated to non biomedical cases.Nevertheless, we agree that such articles have an interdisciplinary nature and are being studied by a broader audience and therefore, we added a section in the introduction discussing the usability of bipartite graphs in a non biomedical context. Simultaneously, we consciously kept this section short in order to avoid information overload and deviate from our initial aim.

Comment: Similar to suggestion (2), tripartite graph and n-partite graph representations would be briefly presented.
Response: A fair point. Presentation of tripartite as well as n-partite graphs has been added in a separate section. Now the text has been expanded to discuss some of their properties while the newly added Figure 10 captures potential uses of existing visualization tools for visualizing tri and n-partite graphs.
Comment: Ranking and graph regularization could also be discussed in the "Models and algorithms for bipartite graphs" section.

Response: We thank the reviewer for highlighting this gap. Ranking and graph regularization are now discussed further in the manuscript, in two new sections. However, we do not go into too much detail as we do not want the non graph theory experts to drop out of interest.
Comment: Though few datasets are mentioned in the conclusion section, however, high-quality

datasets of this domain should be focused after the "Models and algorithms for bipartite graphs" section.
Response: We have now addressed this comment and changed the structure of our text. More specifically we removed the relevant paragraph from the Conclusion section and merged it with our newly added section: Bipartite graphs, the BioMedical Big-Data era and some hints for the future. This part is now under the section: Models and algorithms for bipartite graphs as suggested. Additionally, some collections (databases and repositories) that hold numerous network data, including bipartite ones, are mentioned in a new section.

Comment: Future direction is very limited in the current version. Authors could emphasize on some potential applications of the bipartite graph in network biology.
Response: We fully agree on this. The Editor raised this point too (see our reply above). We expanded the text and tried to put everything in a Big-Data context as well as to discuss some potential applications in Biology and Medicine in more detail. This was missing and we now hope that this addition will significantly improve manuscript's overall quality. Please see section: Bipartite graphs, the BioMedical Big-Data era and hints for the future. More specifically, in this section we initially provide some statistics to describe the Big-Data era in the Bioworld. Once we make clear what are the urgencies here, we discuss how advanced analysis and visualization methods specialized in bipartite graphs could help.

Comment: Though there are many typos, some of which could be spotted with a spell checker.
Minor typos:
1. Tissue specificity is also considered in gene-sidease networks -> "gene-disease"
2. non-versatile ones require describe a limited set of data -> "require a limited set of data"
Response: We thank the reviewer for this comment. The revised version of the manuscript has now been corrected by word's spell checker.
