# Supplementary material for: Bipartite graphs in systems biology and medicine: a survey of methods and applications
Source: Gigascience. 2018 Feb 19;7(4):giy014. doi: 10.1093/gigascience/giy014 (PMC6333914; doi:10.1093/gigascience/giy014)
Supplement: Response_to_Reviewer_Comments_Revision_1.pdf [file giy014_response_to_reviewer_comments_revision_1.pdf]

Author's Response To Reviewer Comments

Dear Editor,
We have updated the manuscript in order to meet your comments. We added the URLs in the references, we tried to improve the language, and we added the "availability of data" along with the supplementary data.
Thank you in advance for your help and efforts
Regards
Pantelis Bagos
