# Supplementary material for: Bipartite graphs in systems biology and medicine: a survey of methods and applications
Source: Gigascience. 2018 Feb 19;7(4):giy014. doi: 10.1093/gigascience/giy014 (PMC6333914; doi:10.1093/gigascience/giy014)
Supplement: Reviewer_1_Report_(Original_Submission) -- Yang Zhou — 20 Aug 2017 Reviewed [file giy014_reviewer_1_report_(original_submission).pdf]

Reviewer Report

Title: Bipartite Graphs in Systems Biology and Medicine: a survey of methods and applications

Version: Original Submission **Date:** 20 Aug 2017

Reviewer name: Yang Zhou

Reviewer Comments to Author:

This paper discussed the topological properties of bipartite graphs, multiple types of real-life network, a couple of software and tools in network biology, database, data mining, and machine learning, and their potential applications in network biology.

My main concern is that I am not sure whether this manuscript is relevant to the readers in the GigaScience community since it is more like a survey paper of the applications of bipartite graphs in network biology, or a textbook chapter or the lecture notes, to introduce bipartite graphs, evaluation measures, real-life networks, software and tools in related literatures.

If this paper is a research / theoretical - oriented paper, then it is better to propose the novel technical solutions on evaluation measures and analytics algorithms of bipartite graphs. If this paper is a practice / system - oriented paper, the systems on large-scale real-life networks should be developed and implemented to improve the scalability and efficiency of bipartite graph analysis. If this paper is an experiment / case study - oriented paper, the experiments on either synthetic or real datasets should be conducted to compare the state-of-the -art tools' effectiveness and efficiency.

Graph matching, link prediction, and community detection / graph partitioning have been well studied in many literatures in network biology, database, data mining, and machine learning. The authors failed to identify and discuss many recent references in these fields.

Overall, I think the journal manuscript has very limited research contributions compared with related literatures. Therefore I would suggest that the authors should seriously consider new aspects of the problems, e.g. adding new algorithms, new systems, or new experiments, to further improve the manuscript.

Level of Interest

Please indicate how interesting you found the manuscript: An article of importance in its field

Quality of Written English

Please indicate the quality of language in the manuscript: Needs some language corrections before being published

Declaration of Competing Interests

Please complete a declaration of competing interests, considering the following questions:

- Have you in the past five years received reimbursements, fees, funding, or salary from an organisation that may in any way gain or lose financially from the publication of this manuscript, either now or in the future?
- Do you hold any stocks or shares in an organisation that may in any way gain or lose financially from the publication of this manuscript, either now or in the future?
- Do you hold or are you currently applying for any patents relating to the content of the manuscript?
- Have you received reimbursements, fees, funding, or salary from an organization that holds or has applied for patents relating to the content of the manuscript?
- Do you have any other financial competing interests?
- Do you have any non-financial competing interests in relation to this paper?

If you can answer no to all of the above, write 'I declare that I have no competing interests' below. If your reply is yes to any, please give details below.

I declare that I have no competing interests.

I agree to the open peer review policy of the journal. I understand that my name will be included on my report to the authors and, if the manuscript is accepted for publication, my named report including any attachments I upload will be posted on the website along with the authors' responses. I agree for my report to be made available under an Open Access Creative Commons CC-BY license (http://creativecommons.org/licenses/by/4.0/). I understand that any comments which I do not wish to be included in my named report can be included as confidential comments to the editors, which will not be published.

I agree to the open peer review policy of the journal

To further support our reviewers, we have joined with Publons, where you can gain additional credit to further highlight your hard work (see: https://publons.com/journal/530/gigascience). On publication of this paper, your review will be automatically added to Publons, you can then choose whether or not to claim your Publons credit. I understand this statement.

Yes
