# Supplementary material for: Bipartite graphs in systems biology and medicine: a survey of methods and applications
Source: Gigascience. 2018 Feb 19;7(4):giy014. doi: 10.1093/gigascience/giy014 (PMC6333914; doi:10.1093/gigascience/giy014)
Supplement: Reviewer_2_Report_(Original_Submission) -- Md Zia Ullah, Ph.D. — 04 Oct 2017 Reviewed [file giy014_reviewer_2_report_(original_submission).pdf]

Reviewer Report

Title: Bipartite Graphs in Systems Biology and Medicine: a survey of methods and applications

Version: Original Submission **Date:** 04 Oct 2017

Reviewer name: Md Zia Ullah, Ph.D.

Reviewer Comments to Author:

This paper systematically presents the usability of a bipartite graph in the field of network biology. Topological properties of the bipartite graph and bipartite biological networks, including ecological network, bio-molecular network, biomedical network, and epidemiological network are also reviewed. Moreover, some algorithms and software packages related to the bipartite graph are presented.

The paper is well written and easy to follow. Relevance work and relevant researchers are cited.

However, several points could be addressed to improve the current version of this paper.
My suggestions are shown below:
1. The bipartite graph has been extensively used to model the relationship such as Query and URL [A] in query logs, Video shots and Tags [B], Entity and Co-List in a Web page [C], Users and Items in recommendation [D, E]. Some of these citations could be included in the paper.
A. A Generalized Co-HITS Algorithm and Its Application to Bipartite Graphs, Deng et al. (2009)
B. "Visualtextualrank: An extension of visual rank to large-scale video shot extraction exploiting tag co-occurrence, K. YANAI et al. (2015)
C. Bipartite graph based entity ranking for related entity finding, Cao et al. (2011)
D. Recommendation as link prediction in bipartite graphs: A graph kernel-based machine learning approach, Xin Li and Hsinchun Chen (2013)
E. BiRank: Towards Ranking on Bipartite Graphs, He et al. (2017)

2. Similar to suggestion (2), tripartite graph and n-partite graph representations would be briefly presented.

3. Ranking and graph regularization could also be discussed in the "Models and algorithms for bipartite graphs" section.

4. Though few datasets are mentioned in the conclusion section, however, high-quality datasets of this domain should be focused after the "Models and algorithms for bipartite graphs" section.

5. Future direction is very limited in the current version. Authors could emphasize on some potential applications of the bipartite graph in network biology.

Though there are many typos, some of which could be spotted with a spell checker.

Minor typos:
1. Tissue specificity is also considered in gene-sidease networks -> "gene-disease"
2. non-versatile ones require describe a limited set of data -> "require a limited set of data"

Level of Interest

Please indicate how interesting you found the manuscript: An article of importance in its field

Quality of Written English

Please indicate the quality of language in the manuscript: Needs some language corrections before being published

Declaration of Competing Interests

Please complete a declaration of competing interests, considering the following questions:

- Have you in the past five years received reimbursements, fees, funding, or salary from an organisation that may in any way gain or lose financially from the publication of this manuscript, either now or in the future?
- Do you hold any stocks or shares in an organisation that may in any way gain or lose financially from the publication of this manuscript, either now or in the future?
- Do you hold or are you currently applying for any patents relating to the content of the manuscript?
- Have you received reimbursements, fees, funding, or salary from an organization that holds or has applied for patents relating to the content of the manuscript?
- Do you have any other financial competing interests?
- Do you have any non-financial competing interests in relation to this paper?

If you can answer no to all of the above, write 'I declare that I have no competing interests' below. If your reply is yes to any, please give details below.

I declare that I have no competing interests

I agree to the open peer review policy of the journal. I understand that my name will be included on my report to the authors and, if the manuscript is accepted for publication, my named report including any attachments I upload will be posted on the website along with the authors' responses. I agree for my report to be made available under an Open Access Creative Commons CC-BY license

(http://creativecommons.org/licenses/by/4.0/). I understand that any comments which I do not wish to be included in my named report can be included as confidential comments to the editors, which will not be published.

I agree to the open peer review policy of the journal

To further support our reviewers, we have joined with Publons, where you can gain additional credit to further highlight your hard work (see: https://publons.com/journal/530/gigascience). On publication of this paper, your review will be automatically added to Publons, you can then choose whether or not to claim your Publons credit. I understand this statement.

Yes
